# You Win Some, You Lose Some: Modifying the Molecular Periphery of Nitrofuran-Tagged Diazaspirooctane Reshapes Its Antibacterial Activity Profile

**DOI:** 10.3390/ijms26010207

**Published:** 2024-12-29

**Authors:** Lyubov Vinogradova, Kristina Komarova, Alexey Lukin, Maxim Zhuravlev, Dmitry Deniskin, Anastasia Poliakova, Mikhail Chudinov, Maxim Gureev, Marine Dogonadze, Tatiana Vinogradova, Elizaveta Rogacheva, Lyudmila Kraeva, Yuri Porozov, Viktor Korzhikov-Vlakh

**Affiliations:** 1 Lomonosov Institute of Fine Chemical Technologies, MIREA—Russian Technological University, Moscow 119454, Russia; vlv010599@yandex.ru (L.V.); kristinka-komarova.1999@mail.ru (K.K.); alex-look@yandex.ru (A.L.); max.2903@mail.ru (M.Z.); deniskin.02d@mail.ru (D.D.); polyakova.a.e@edu.mirea.ru (A.P.); 2 Institute of Cytology, Russian Academy of Sciences, Saint Petersburg 194064, Russia; max_technik@mail.ru; 3 Saint-Petersburg State Research Institute of Phthisiopulmonology of the Ministry of Healthcare of the Russian Federation, Saint Petersburg 191036, Russia; marine-md@mail.ru (M.D.); vinogradova@spbniif.ru (T.V.); 4 Pasteur Institute of Epidemiology and Microbiology, Saint Petersburg 197101, Russia; elizvla@yandex.ru (E.R.); lykraeva@yandex.ru (L.K.); 5 Laboratory of Angiopathology, The Institute of General Pathology and Pathophysiology, Moscow 125315, Russia; yporozov@hse.ru; 6 Advitam Laboratory, 11108 Belgrade, Serbia; 7 Department of Medical Chemistry, Institute of Chemistry, Saint Petersburg State University, Saint Petersburg 199034, Russia

**Keywords:** nitrofurans, diazaspirooctane, privileged structures, antibacterial activity, *M. tuberculosis*, *S. aureus*, induced-fit docking

## Abstract

The use of the concept of privileged structures significantly accelerates the search for new leads and their optimization. 6-(methylsulfonyl)-8-(4-methyl-4*H*-1,2,4-triazol-3-yl)-2-(5-nitro-2-furoyl)-2,6-diazaspiro[3.4]octane **1** has been identified as a lead, with MICs of 0.0124–0.0441 μg/mL against *MTb* multiresistant strains. Several series of structural analogues have been synthesized, including variations in the periphery and simplifications of their scaffolds. All synthesized compounds were tested against the *MTb* H37Rv strain and ESKAPE panel of pathogens using serial broth dilutions. However, an attempt to optimize structure of **1** did not lead to the development of more active compounds which can work against *MTb*, but to substances with high activity against *S. aureus*. Induced-fit docking and MM-GBSA calculations determined a change in the likely biotarget from deazaflavin-dependent nitroreductase to azoreductases. The privileged nature of the scaffold was demonstrated by the detection of a different type of activity.

## 1. Introduction

It is a generally accepted definition of the privileged structure in medicinal chemistry that it exhibits biological activity towards certain types of targets more often than other structures [1,2,3,4]. The only criteria here is the frequency of activity detection among molecules containing certain moieties. Privileged scaffolds can be found in different types of organic molecules [5,6,7,8]. The privileged fragment itself is not necessarily a pharmacophore, but its introduction into the molecule makes it pharmacologically active [9,10,11,12].

Is it possible to predict the “privilege” of a scaffold based on its molecular structure? The basic principles of induced fit theory say that the ligand structure should provide the maximum number of binding interactions and be sufficiently rigid. Spirocyclic compounds with heteroatoms are well suited here [13]. Indeed, there are many privileged structures among small spiroheterocycles [14,15,16].

The use of the concept of privileged structures significantly accelerates the search for new leads and their optimization. This is especially important for the development of new antibacterials due to the widespread prevalence of pathogenic bacteria-resistant strains. The emergence of resistance to new drugs is outpacing their development, a problem that could lead to millions of deaths in the coming years [17].

One of the important stages of drug development is the establishment of the mechanism of action of a new substance. As for antibacterial agents containing nitrofuran moieties, this mechanism is known. The nitrofuran group reduces, by reductases, to highly reactive intermediates that inhibit the citric acid cycle as well as the synthesis of DNA, RNA, and proteins. Reductases are the main targets of this type of antibiotics [18,19]. However, allosteric inhibition of other protein targets by the new substance is always possible. So, phosphoglycerate kinase is not usually considered as a target for nitrofurans, but there is an example of enzyme inhibition by a nitrofuran compound [20]. This expands the range of possible targets and mechanisms.

The 2,6-diazaspiro[3.4]octane scaffold is included in many pharmacologically active structures. It is a peripheral moiety of the latest generation fluoroquinolone zabofloxacin (DW-224a) molecule, which is proposed to combat multidrug-resistant Gram-positive infections [21]. Its mechanism of action is DNA gyrase and topoisomerase IV inhibition, common with all fluoroquinolones [22]. Some diazaspirooctane derivatives have been proposed as fusion inhibitors for the treatment of RSV infection [23,24] as well as antimalarial agents [25,26]. The scaffold is a component of certain human enzymes inhibitors and is used as part of the latest protein targeting degradation systems (PROTAC) against prostate cancer [27,28]. Moreover, the diazaspirooctane skeleton is a part of the opioid sigma-1 receptor [29] and tryptophan hydroxylase 1 (TPH1) [30] enzyme inhibitors.

**Figure 1:**
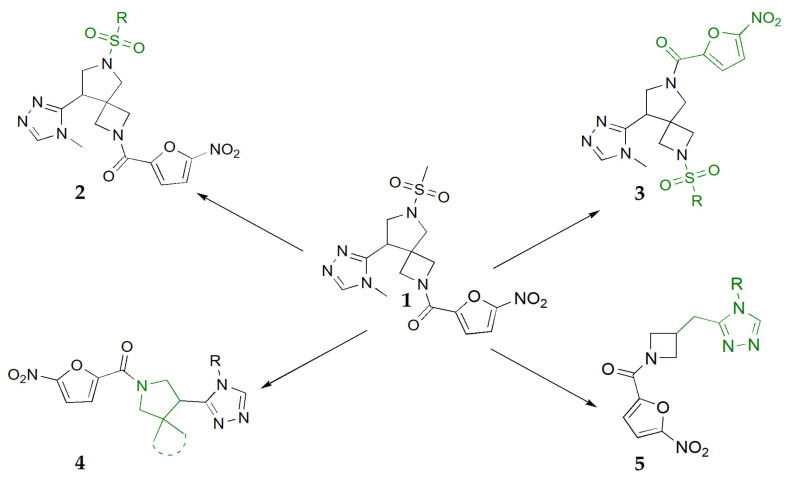
Lead substance **1** and its modification directions. Modifiable fragments are highlighted in green: changing the sulfonyl amide group substituents (series **2**); changing the substituent order and sulfonyl amide group substituents (series **3**); changing the spirocycle structure (series **4**) and simplification (series **5**).

Recent studies have shown that combining a 2,6-diazaspiro[3.4]octane scaffold with a 5-nitrofuranoyl “warhead” results in molecules with superior activity against *M.Tuberculosis* (*MTb*) [31,32]. Among them, 6-(methylsulfonyl)-8- (4-methyl-4*H*-1,2,4-triazol-3-yl)-2-(5-nitro-2-furoyl)-2,6-diazaspiro[3.4]octane **1** (Figure 1) was identified as the lead, with MICs of 0.0124–0.0441 μg/mL against *MTb* multiresistant strains [33]. The next step was to optimize the structure of **1**, which led to unexpected results. In this paper, we present the part of this study that was of greatest interest.

## 2. Results

### 2.1. Chemistry

Lead optimization involves improving the performance of a designed molecule by structural modification. Typically, several series of compounds with different substituents at the periphery are synthesized to understand the role of each fragment in lead performance. Series **2**–**4** (Figure 1) are examples of this strategy.

**Scheme 1:**
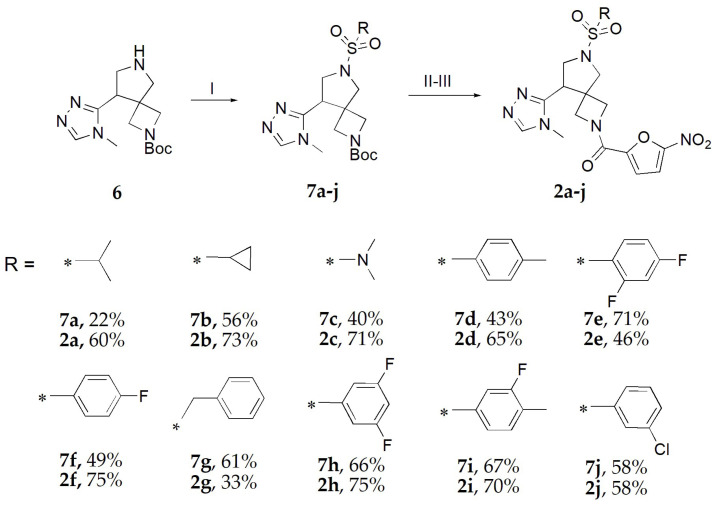
Synthesis of compounds **2a**–**j.** Reagents and conditions: (I) RSO_2_Cl, Et_3_N, CH_2_Cl_2_, 0 °C→ rt, 18 h; (II) CF_3_COOH, CH_2_Cl_2_, 0 °C→ rt, 1 h; (III) 5-nitro-2-furoic acid, CDI, DMF, rt, 1 h.

Compounds of the **2a**–**j** series are complete structural analogues of compound **1** with a modified sulfonyl amide substituent. The starting compound for their synthesis was amine **6**, obtained by method [31] (Scheme 1). By acylation of **6** with various sulfonyl chlorides following deprotection, compounds **7a**–**j** were obtained. Then, **7a**–**j** were reacted with 5-nitro-2-furoic acid and CDI to give **2a**–**j**.

**Scheme 2:**
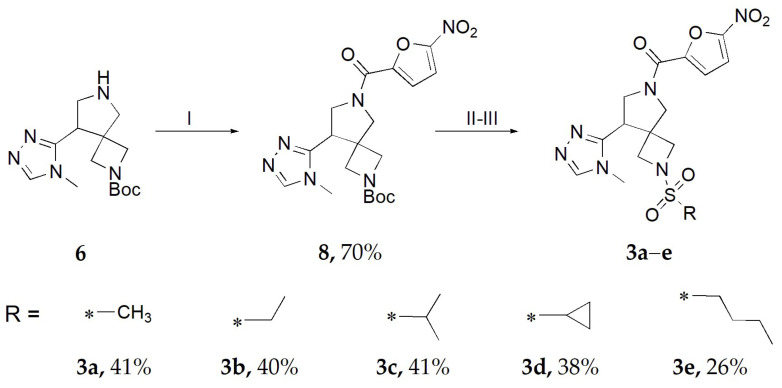
Synthesis of compounds **3a**–**e.** Reagents and conditions: (I) 5-nitro-2-furoic acid, CDI, DMF, rt, 1 h; (II) CF_3_COOH, CH_2_Cl_2_, 0 °C → rt, 1 h; (III) RSO_2_Cl, Et_3_N, CH_2_Cl_2_, 0 °C → rt, 18 h.

The starting compound for the synthesis of **3a**–**e** series was also amine **6**. The synthesis was carried out according to the same scheme, but in reverse order: first acylation with 5-nitro-2-furoic acid, and then with sulfonyl chlorides to give **3a**–**e** (Scheme 2).

**Scheme 3:**
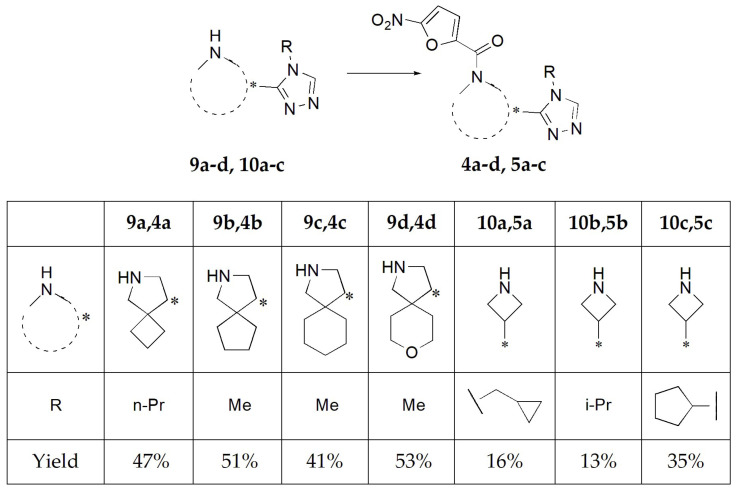
Synthesis of compounds **4a**–**d** and **5a**–**c**. Reagents and conditions: 5-nitro-2-furoic acid, CDI, DMF, rt, 1 h.

Four compounds of series **4** have scaffolds without an azetidine ring. The previously synthesized [34] starting compounds **9a**–**d** were acylated by 5-nitro-2-furoic acid to give **4a**–**e** (Scheme 3).

Finally, the last series of compounds was obtained by simplifying the original scaffold. The pyrrolidine ring was removed from the molecule, resulting in compounds **5a**–**c** (Scheme 3). These compounds were prepared in the same way as series **4** from commercially available azetidines **10a**–**c**.

### 2.2. Antibacterial Activity

All series **2**–**5** were tested against *MTb* (H37Rv strain) and ESKAPE pathogens by serial dilution. The clinically-used antibiotics isoniazid and ethambutol were used as positive controls and comparators against *MTb*. Since testing was performed on the entire ESKAPE panel and classical nitrofurans are ineffective against some pathogens in the panel, the more active ciprofloxacin was used as a control compound. The results shown in Table 1 seemed somewhat disappointing to us: apart from the lead compound, only a few compounds showed weak antitubercular activity. This completely disappeared, even in the closest structural analogues of compound **1**. However, a number of tested compounds showed unexpectedly high activity against *S. aureus* only. None of the new substances showed activity at concentrations below 100 µg/mL against the remaining ESKAPE pathogens. This turned out to be a very interesting result considering the privileged nature of the scaffold.

### 2.3. Molecular Modeling

Molecular modeling of ligand–protein complexes with the main biological targets of *MTb* (H37Rv strain) and *S. aureus* was carried out to determine the reasons for the dramatic activity change. Induced-fit docking (IFD) was used in calculations in combination with MM-GBSA (Molecular Mechanics with generalized Born and Surface Area solvation). IFD is a modeling method that allows for the prediction of ligand-induced changes in a target structure. It is a variation of the molecular docking method that considers the possible protein flexibility induced by interactions with small molecules within a given radius (in this case, 6 Å from the ligand). This approach allows for more accurate calculation of the resulting systems’ energy parameters and identification of the potential causes of activity changes.

The following proteins were selected as *Mtb* targets: deazaflavin-dependent nitroreductase (Ddn), arylamine-N-acetyltransferase (TBNAT) and transcriptional regulatory repressor protein (EthR). For *S. aureus*, the targets were the following: azoreductase (AzoR) and oxygen-insensitive NADPH nitroreductases (NfsA and NfsB) as main targets, and phosphoglycerate kinase (Pgk) and dihydrofolate reductase (DHFR) as side targets. The calculation results are shown in Table 2.

## 3. Discussion

There is a good correlation between anti-tuberculosis activity and calculation results. Compound **1** selectively interacts with the Ddn protein. With other *MTb* proteins (EthR and TBNAT), the binding is weaker and the folding quality is worse. Weakly active compounds **4a** and **5a**–**c** also demonstrated affinity for Ddn, in contrast to inactive **2b** and **4c**. The DDN protein is clearly a priority target for compound **1**. EthR and TBNAT proteins can be excluded from the pool of potential targets since all, without exception, docking solutions have low quality and folding stability with a complete lack of correlation with experimental data.

**Figure 2:**
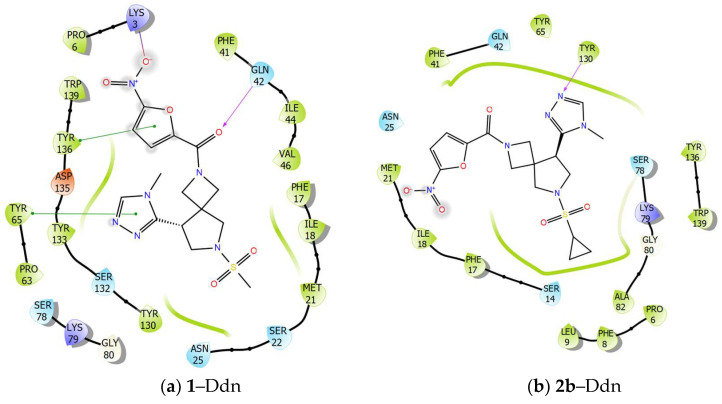

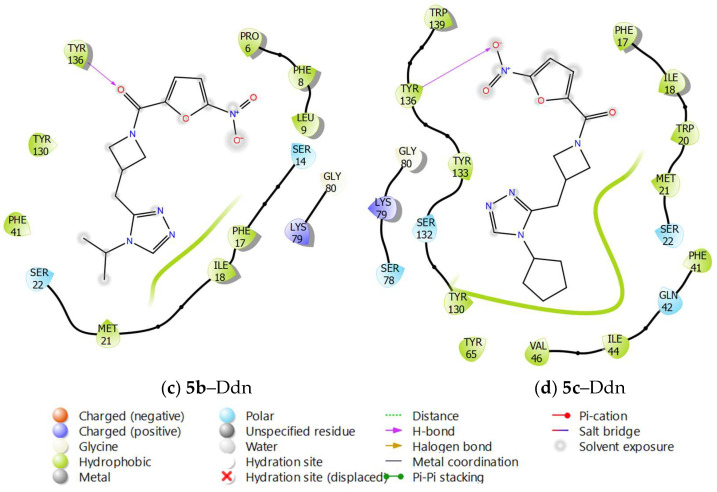
Ligand interactions diagrams of **1**, **2b, 5b** and **5c** with Ddn protein.

The molecular mechanism of reducing anti-tuberculosis activity is demonstrated in Figure 2. This figure shows the three-dimensional structures of the ligand–protein complexes of compounds **1**, **2b**, **5b**, and **5c** with the Ddn protein. In pair **1** vs. **2b**, the introduction of a lipophilic cyclopropyl substituent leads to an imbalance of lipophilic contacts. This causes a turn of the molecule in the active site due to an increase in the density of lipophilic contacts with Pro6, Phe8, Leu9, Ala82, and Trp139 (Figure 2b). The interaction between the nitro group and the catalytic center of the Ddn protein (within Tyr130/133/136, as illustrated in Figure 2a) is disrupted.

In the pair of ligands **1** and **5c**, the latter retains moderate activity due to the location of the nitro group in the active center within the amino acids Tyr130/133/136. The cyclopentyl substituent in the 1,2,4-triazole fragment of the molecule **5c** partially retains the attractive potential of lipophilic contacts with Tyr130/133, Ile44, and Val46 and ensures the contact of the nitrofuran part with the active center of Ddn (Figure 2d). The absence of a spirocyclic core in **5b** (Figure 2c), which increases the conformational lability of the ligand, together with the small size of the lipophilic substituent in the 1,2,4-triazole fragment, leads to destabilization of the nitrofuran group contact in the active center. This leads to a significant decrease in antitubercular activity level.

Furthermore, a correlation can be observed between the activity against *S. aureus* and the calculated results. The highest scoring function value and the optimal ligand binding quality within the active center of AzoR are inherent to the most active compounds, **2b** and **5c**. This makes AzoR a priority target. The remaining compounds in the series show a blurred differentiation between low- and moderately active compounds. For example, **4a** and **4c** differ from **5a**,**b** by only 0.17–0.19 kcal/mol.

A similar situation is with NfsA and NfsB: compounds **2b** and **5c** are in the lead, but moderately active compounds **4a** and **4c** are poorly differentiated from low-active ones. However, the general trend of changes in biological activity coincides with calculations.

Dihydrofolate reductase DHFR and phosphoglycerate kinase Pgk, considered potential side targets, did not yield any pronounced correlation with the biological experiment; therefore, it is highly likely that these proteins are not priority targets for the tested compounds.

**Figure 3:**
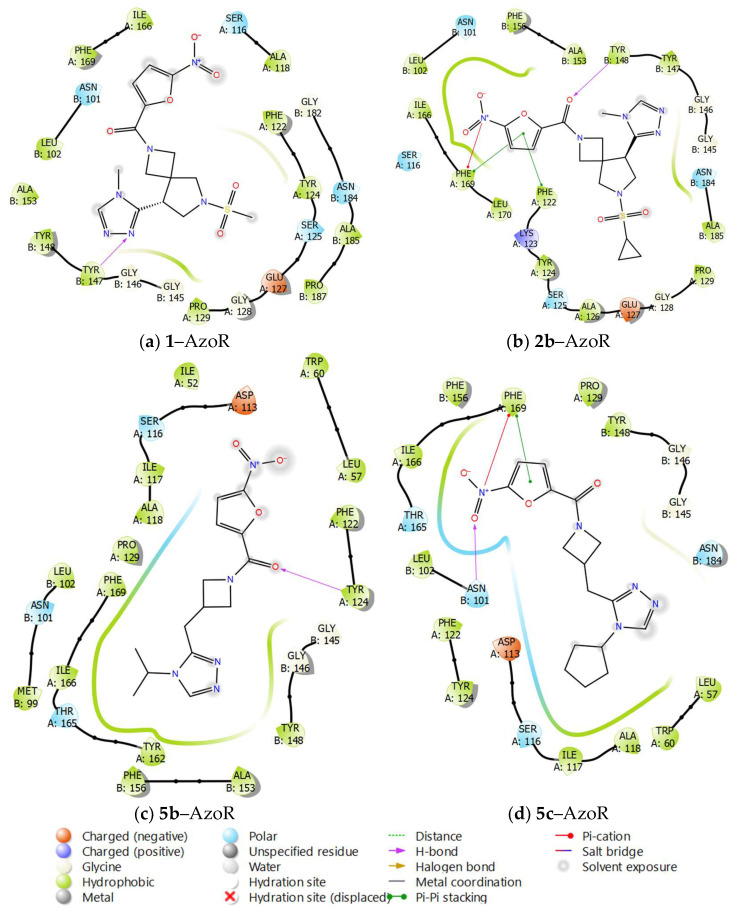
Ligand interactions diagrams of **1**, **2b**, **5b** and **5c** with AzoR protein.

Figure 3 shows the formation of complexes with a preferred AzoR target. The loss of activity towards AzoR in pair **1** vs. **2b** is due to the cyclopropyl sulfonamide moiety influence. The cyclopropyl substituent in molecule **2b** reduces the steric lability of the ligand in the active cavity due to the formation of lipophilic contacts with Tyr124, Ala185/126, and Pro129. The nitrofuran group of compound **1** is incorrectly oriented towards the solvent (Figure 3a). In the complex of **2b** with AzoR (Figure 3b), on the contrary, we observe the interaction of the nitrofuran group in the active site, where the flavin mononucleotide is bound, within the Phe122/169 zone. Directing π-stacking and π-cationic interactions are observed too. A comparison of the low-activity compound 5b with 5c (Figure 3c,d) reveals that a reduction in the lipophilic substituent volume, as observed in the previous case, has a detrimental impact on molecular recognition.

**Figure 4:**
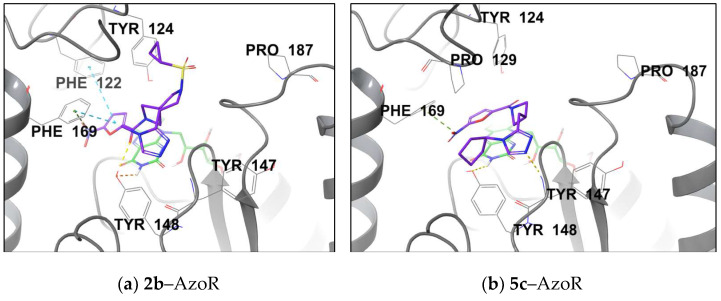
Binding comparison of **2b** and **5c** in AzoR active site. Tested compound in purple color, co-factor flavin mononucleotide (FMN) in green color. Dashed lines: yellow—hydrogen bond; light blue—π-stacking; green—π–cation interaction.

Compound **5c** retains the patterns of ligand–protein contacts, π-stacking and π-cationic interaction of the nitrofuran group with Phe169 and lipophilic contacts, as in compound **2b**. In terms of binding pose, compounds **2b** and **5c** form ligand–protein complexes that are similar in geometry (Figure 4).

## 4. Materials and Methods

### 4.1. Chemistry

Commercially available reagents were used without further purification. NMR spectra were recorded using DPX-300 spectrometer (Bruker Corporation, MA, USA) (^1^H: 300 MHz; ^13^C: 75 MHz). Chemical shifts are reported as parts per million (δ, ppm). The residual solvent peak (CHCl_3_ or DMSO-d_6_) was used as internal standard: 7.28 or 2.51 for ^1^H and 77.07 or 40.00 ppm for ^13^C. Multiplicities are abbreviated as follows: s = singlet; d = doublet; t = triplet; q = quartet; m = multiplet; br = broad; dd = doublet of doublets; dt = doublet of triplets. Coupling constants, *J*, are reported in Hz. Mass spectra were recorded using Bruker microTOF spectrometer (Bruker Corporation, MA, USA) (ionization by electrospray, positive ion detection). Melting points were determined in open capillary tubes using Stuart SMP50 Automatic Melting Point Apparatus (Cole-Parmer, IL, USA). Analytical thin-layer chromatography was carried out on UV-254 silica gel plates. Visualization was accomplished by UV light (254 nm). Column chromatography was performed with 230−400 mesh silica gel, Merk grade 60 (0.040−0.063 mm). All reactions were carried out under argon atmosphere. Compounds **10a**–**c** were purchased from Alinda Company (Moscow, Russia).

#### 4.1.1. Synthesis of **2** Series

##### General Procedure for the Synthesis of Compounds **7a**–**j**

To a solution of 0.25 g of *tert*-butyl 8-(4-methyl-4*H*-1,2,4-triazol-3-yl) -2,6-diazaspiro[3.4]octane-2-carboxylate **6** (0.99 mmol) and 0.23 g of triethylamine (2.28 mmol) in 25 mL of CH_2_Cl_2_, a solution of 1.5 mmol of the corresponding sulfonyl chloride in 15 mL of CH_2_Cl_2_ was added dropwise while cooling the mixture to 0 °C. The resultant mixture was allowed to reach room temperature and was then stirred for 18 h. Then, the mixture was poured in water (30 mL); the organic phase was separated and washed with 20 mL of 5% (aq) K_2_CO_3_. The organic phase was then separated again, dried over anhydrous Na_2_SO_4_, filtered, and concentrated in vacuo. The residue was purified by column chromatography on silica gel eluting with 0% → 2.5% methanol in CH_2_Cl_2_ to give the title compound. Compounds **7a**–**j** were characterized by LC-MS and used in the next step.

##### General Procedure for the Synthesis of Compounds **2a**–**j**

0.2 mmol of compound **7** was dissolved in a 1:1 mixture of CF_3_COOH:CH_2_Cl_2_ (1.5 mL). The mixture was stirred for 1 h at 0 °C. After that, the mixture was evaporated in vacuo. The residue was used in the next step without further purification.

A total of 0.032 g of N,N′-carbonyldiimidazole (0.2 mmol, 1.25 eq) and 0.025 g of 5-nitro-2-furoic acid (0.16 mmol, 1 eq) were dissolved in 1mL of dry dimethylformamide. The mixture was stirred for 20 min. Then, a solution of 1.25 equiv of deprotected **7** and 0.081 g of triethylamine (0.8 mmol, 5 equiv) in 1 mL of dry DMF was added. The mixture was stirred for 1 h, then poured into water (5 mL) and extracted with ethyl acetate (3 × 5 mL). The organic phase was washed with 5% K_2_CO_3_ solution (5 mL) and brine (5 mL), dried over Na_2_SO_4_, and evaporated. The residue was purified by column chromatography on silica gel, eluting with 0% → 10% methanol in CH_2_Cl_2_. Fractions containing the target compound **2** were combined and evaporated under vacuum.

*6-(isopropylsulfonyl)-8-(4-methyl-4H-1,2,4-triazol-3-yl)-2-(5-nitro-2-furoyl)-2,6-diazaspiro[3.4]octane **2a***

Yield: 0.042 g (60%) from 0.080 g of **7a**, white solid, Mp 160–161 °C. ^1^H-NMR (300 MHz, DMSO–d_6_) δ 8.45 (s, 1H), 7.78 (d, *J* = 3.9 Hz, 0,5H), 7.73 (d, *J* = 3.9 Hz, 0,5H), 7.36–7.26 (m, 1H), 4.56 (s, 2H), 4.14–3,97 (m, 4H), 3.89–3.71 (m, 3H), 3.66 (s, 3H), 3.52–3.46(m, 1H), 1.23–1.14 (m, 6H); ^13^C-NMR (75 MHz, DMSO–d_6_) δ 156.68, 152.39, 151.83, 147.72, 145.58, 117.41, 113.48, 59.26, 57.60, 56.82, 51.38, 44.05, 43.96, 30.89, 16.67, 16.64; LCMS (ESI), *m*/*z*: 439.5 [M+H]^+^; HRMS (ESI) *m*/*z* calculated for C_17_H_23_N_6_O_6_S [M+H]^+^439.1399 Da, found 439.1373 Da.

*6-(cyclopropylsulfonyl)-8-(4-methyl-4H-1,2,4-triazol-3-yl)-2-(5-nitro-2-furoyl)-2,6-diazaspiro[3.4]octane **2b***

Yield: 0.256 g (73%) from 0.400 g of **7b**, white solid, Mp 175–176 °C. ^1^H-NMR (300 MHz, DMSO–d_6_) δ 8.46 (s, 1H), 7.79 (d, *J* = 3.8 Hz, 0,5H), 7.75 (d, *J* = 3.8 Hz, 0,5H),7.32 (s, 1H), 4.67–4.51 (m, 2H), 4.19–3.99 (m, 4H), 3.91–3.61 (m, 5H), 3.55–3.46 (m, 1H), 2.68–2.55 (m, 1H), 1.02–0.72 (m, 4H); ^13^C-NMR (75 MHz, DMSO–d_6_) δ 156.67, 156.61, 152.23, 151.83, 147.79, 147.71, 145.68, 117.44, 113.55, 113.50, 62.60, 59.47, 57.60, 56.68, 53.97, 51.33, 51.19, 43.98, 43.86, 30.91, 30.88, 25.74, 25.67, 4.14; LCMS (ESI), *m*/*z*: 437.4 [M+H]^+^; HRMS (ESI) *m*/*z* calcd for C_17_H_21_N_6_O_6_S [M+H^+^] 437.1243 Da, found 437.1231 Da.

*N* *,N-dimethyl-8-(4-methyl-4H-1,2,4-triazol-3-yl)-2-(5-nitro-2-furoyl)-2,6-diazaspiro[3.4]octane-6-sulfonamide **2c***

Yield: 0.105 g (71%) from 0.170 g of **7c**, white solid, Mp 168–169 °C. ^1^H-NMR (300 MHz, DMSO–d_6_) 8.46 (s, 1H), 7.79 (d, *J* = 3.9 Hz, 0,5H), 7.74 (d, *J* = 3.9 Hz, 0,5H), 7.33 (d, *J* = 3.9 Hz, 0,5H), 7.30 (d, *J* = 3.9 Hz, 0,5H),4.55 (d, *J* = 4.0 Hz, 2H), 4.17–3.91 (m, 4H), 3.80–3.63 (m, 5H), 3.47–3.38 (m, 1H), 2.70 (d, *J* = 5.2 Hz, 6H); ^13^C-NMR (75 MHz, DMSO–d_6_) δ 156.66, 152.45, 151.83, 147.80, 147.71, 145.61, 117.44, 113.55, 113.50, 62.70, 59.31, 57.68, 57.26, 53.95, 51.89, 51.79, 43.94, 43.85, 38.12, 30.90; LCMS (ESI), *m*/*z*: 440.5 [M+H]^+^; HRMS (ESI) *m*/*z* calcd for C_16_H_22_N_7_O_6_S [M+H^+^] 440.1352 Da, found 440.1359 Da.

*6-[(4-methylphenyl)sulfonyl]-8-(4-methyl-4H-1,2,4-triazol-3-yl)-2-(5-nitro-2-furoyl)-2,6-diazaspiro[3.4]octane **2d***

Yield: 0.100 g (65%) from 0.180 g of **7d**, white solid, Mp 158–159 °C. ^1^H-NMR (300 MHz, DMSO–d_6_) δ 8.35 (s, 1H), 7.78–7.64 (m, 3H), 7.41 (2s, 2H), 7.22 (t, *J* = 4.1 Hz, 1H), 4.46–4.12 (m, 2H), 3.97–3.68 (m, 5H), 3.66–3.55 (m, 5H), 2.41 (s, 3H); ^13^C-NMR (75 MHz, DMSO–d_6_) mixture of rotamers δ 156.54, 156.45, 151.65, 147.78, 147.62, 145.49, 144.18, 144.12, 133.27, 133.22, 130.24, 127.66, 127.62, 117.39, 113.47, 62.01, 58.63, 57.47, 56.78, 53.76, 51.37, 51.22, 43.61, 43.51, 30.80, 21.45; LCMS (ESI), *m*/*z*: 487.5 [M+H]^+^; HRMS (ESI) *m*/*z* calcd for C_21_H_23_N_6_O_6_S [M+H^+^] 487.1399 Da, found 487.1408 Da.

*6-[(2,4-difluorophenyl)sulfonyl]-8-(4-methyl-4H-1,2,4-triazol-3-yl)-2-(5-nitro-2-furoyl)-2,6-diazaspiro[3.4]octane **2e***

Yield: 0.120 g (46%) from 0.300 g of **7e**, white solid, Mp 163–164 °C. ^1^H-NMR (300 MHz, DMSO–d_6_) δ 8.36 (d, *J* = 2.7 Hz, 1H), 7.98–7.63 (m, 2H), 7.52–7.38 (m, 1H), 7.33–7.18 (m, 2H), 4.54–4.39 (m, 2H), 4.09–3.89 (m, 4H), 3.87–3.72 (m, 2H), 3.64–3.50 (m, 4H); ^13^C-NMR (75 MHz, DMSO–d_6_) δ 156.64, 151.85 (d, *J* = 10.4 Hz), 147.77 (d, *J* = 6.8 Hz), 145.46, 133.07 (d, *J* = 10.8 Hz), 122.05 (dd, *J* = 15.1, 3.7 Hz), 117.38, 113.44, 112.68 (d, *J* = 22.0 Hz), 106.68 (t, *J* = 26.8 Hz), 59.50, 57.28, 56.21, 53.57, 51.18, 43.98, 43.86, 30.78; LCMS (ESI), *m*/*z*: 509.4 [M+H]^+^; HRMS (ESI) *m*/*z* calcd for C_20_H_19_F_2_N_6_O_6_S [M+H^+^] 509.1055 Da, found 509.1041 Da.

*6-[(4-fluorophenyl)sulfonyl]-8-(4-methyl-4H-1,2,4-triazol-3-yl)-2-(5-nitro-2-furoyl)-2,6-diazaspiro[3.4]octane **2f***

Yield: 0.161 g (75%) from 0.250 g of **7f**, white solid, Mp 174–175 °C. ^1^H-NMR (300 MHz, DMSO–d_6_) mixture of rotamers δ 8.34 (d, *J* = 3.6 Hz, 1H), 7.90–7.81 (m, 2H), 7.77 (d, *J* = 3.9 Hz, 0,5H), 7.72 (d, *J* = 3.9 Hz, 0,5H),7.40 (td, *J* = 8.9, 2.4 Hz, 2H), 7.30–7.22 (m, 1H), 4.44–4.16 (m, 2H), 3.98–3.61 (m, 6H), 3.58 (s, 3H), 1.17 (t, *J* = 7.3 Hz, 1H); ^13^C-NMR (75 MHz, DMSO–d_6_) mixture of rotamers δ 165.08 (d, *J* = 251.7 Hz), 156.57, 156.50, 151.74, 151.70, 147.79, 147.67, 145.44, 132.65 (d, *J* = 2.2 Hz), 130.59 (dd, *J* = 9.7, 4.9 Hz), 117.40, 116.93 (d, *J* = 22.6 Hz), 113.46, 62.31, 58.93, 57.32, 56.66, 53.60, 51.46, 51.34, 43.70, 43.59, 30.78; LCMS (ESI), *m*/*z*: 491.5 [M+H]^+^; HRMS (ESI) *m*/*z* calcd for C_20_H_20_FN_6_O_6_S [M+H^+^] 491.1149 Da, found 491.1155 Da.

*6-(benzylsulfonyl)-8-(4-methyl-4H-1,2,4-triazol-3-yl)-2-(5-nitro-2-furoyl)-2,6-diazaspiro[3.4]octane **2g***

Yield: 0.070 g (33%) %) from 0.244 g of **7g**, white solid, Mp 169–170 °C. ^1^H-NMR (300 MHz, DMSO–d_6_) mixture of rotamers δ 8.47 (s, 1H), 7.80 (d, *J* = 3.9 Hz, 0,5H), 7.75 (d, *J* = 3.9 Hz, 0,5H),7.38–7.27 (m, 6H), 4.64–4.35 (m, 4H), 4.16–3.93 (m, 4H), 3.85–3.75 (m, 1H), 3.67–3.50 (m, 5H); ^13^C-NMR (75 MHz, DMSO–d_6_) mixture of rotamers δ 156.66, 156.59, 152.16, 152.10, 151.84, 147.87, 147.71, 145.67, 131.25, 130.06, 128.72, 128.50, 117.44, 113.55, 113.48, 62.69, 59.09, 57.75, 56.56, 54.83, 54.70, 53.99, 51.20, 51.06, 44.01, 43.91, 30.86; LCMS (ESI), *m*/*z*: 487.5 [M+H] ^+^; HRMS (ESI) *m*/*z* calcd for C_21_H_23_N_6_O_6_S [M+H^+^] 487.1399 Da, found 487.1381 Da.

*6-[(3,5-difluorophenyl)sulfonyl]-8-(4-methyl-4H-1,2,4-triazol-3-yl)-2-(5-nitro-2-furoyl)-2,6-diazaspiro[3.4]octane **2h***

Yield: 0.213 g (75%) from 0.328 g of **7h**, white solid, Mp 161–162 °C. ^1^H-NMR (300 MHz, DMSO–d_6_) mixture of rotamers δ 8.34 (s, 0,5H), 8.32 (s, 0,5H), 7.79 (d, *J* = 3.9 Hz, 0,5H), 7.73 (d, *J* = 3.9 Hz, 0,5H), 7.67–7.56 (m, 1H), 7.54–7.43 (m, 2H), 7.28 (t, *J* = 4.3 Hz, 1H), 4.53–4.31 (m, 2H), 4.03–3.67 (m, 7H), 3.60 (s, 3H); ^13^C-NMR (75 MHz, DMSO–d_6_) mixture of rotamers δ 162.67 (dd, *J* = 251.6, 12.9 Hz), 156.59, 156.54, 151.75, 147.79, 147.70, 145.36, 139.67 (t, *J* = 8.0 Hz), 117.41, 113.53, 113.46, 111.28 (dd, *J* = 28.0, 6.8 Hz), 109.49 (t, *J* = 25.7 Hz), 62.67, 59.44, 57.17, 56.55, 53.48, 51.57, 51.47, 43.81, 43.68, 30.76, 30.73; LCMS (ESI), *m*/*z*: 509.5 [M+H]^+^; HRMS (ESI) *m*/*z* calcd for C_20_H_19_F_2_N_6_O_6_S [M+H^+^] 509.1055 Da, found 509.1040 Da.

*6-[(3-fluoro-4-methylphenyl)sulfonyl]-8-(4-methyl-4H-1,2,4-triazol-3-yl)-2-(5-nitro-2-furoyl)-2,6-diazaspiro[3.4]octane **2i***

Yield: 0.170 g (70%) from 0.280 g of **7i**, white solid, Mp 176–177 °C. ^1^H-NMR (300 MHz, DMSO–d_6_) mixture of rotamers δ 8.34 (d, *J* = 5.1 Hz, 1H), 7.78 (d, *J* = 3.9 Hz, 0.5H), 7.72 (d, *J* = 3.8 Hz, 0.5H), 7.58–7.46 (m, 3H), 7.30–7.21 (m, 1H), 4.48–4.21 (m, 2H), 3.98–3.83 (m, 4H), 3.79–3.64 (m, 2H), 3.59 (s, 3H), 3.50–3.42 (m, 1H), 2.32 (s, 3H); ^13^C-NMR (75 MHz, DMSO–d_6_) δ 160.63 (d, *J* = 247.8 Hz), 156.59, 151.75, 151.64, 145.38, 135.80 (d, *J* = 6.4 Hz), 132.95 (d, *J* = 5.0 Hz), 130.96 (d, *J* = 16.9 Hz), 123.51 (d, *J* = 3.2 Hz), 117.30, 114.08 (d, *J* = 25.2 Hz), 113.38, 62.39, 58.81, 57.48, 56.75, 53.71, 51.33, 43.76, 30.77, 14.64 (d, *J* = 3.5 Hz); LCMS (ESI), *m*/*z*: 505.5 [M+H] ^+^; HRMS (ESI) *m*/*z* calcd for C_21_H_22_FN_6_O_6_S [M+H^+^] 505.1306 Da, found 505.1313 Da.

*6-[(3-chlorophenyl)sulfonyl]-8-(4-methyl-4H-1,2,4-triazol-3-yl)-2-(5-nitro-2-furoyl)-2,6-diazaspiro[3.4]octane **2j***

Yield: 0.145 g (58%) from 0.289 g of **7j**, white solid, Mp 159–160 °C. ^1^H-NMR (300 MHz, DMSO–d_6_) mixture of rotamers δ 8.34 (s 1H), 7.84–7.68 (m, 4H), 7.67–7.55 (m, 1H), 7.26 (t, *J* = 3.9 Hz, 1H), 4.41 (s, 1H), 4.38–4.23 (m, 1H), 4.02–3.63 (m, 6H), 3.60 (s, 3H), 3.56–3.43 (m, 1H); ^13^C-NMR (75 MHz, DMSO–d_6_) mixture of rotamers δ 156.58, 156.52, 151.78, 151.69, 151.67, 147.78, 147.66, 145.47, 138.18, 134.53, 133.72, 131.73, 126.96, 126.90, 126.31, 126.25, 117.42, 113.54, 113.48, 62.28, 59.03, 57.27, 56.64, 53.60, 51.47, 51.35, 43.72, 43.60, 30.79, 30.76; LCMS (ESI), *m*/*z*: 507.9 [M+H]^+^; HRMS (ESI) *m*/*z* calcd for C_20_H_20_ClN_6_O_6_S [M+H^+^] 507.0854 Da, found 507.0861 Da.

#### 4.1.2. Synthesis of **3** Series

*tert*-butyl *8-(4-methyl-4H-1,2,4-triazol-3-yl)-6-(5-nitro-2-furoyl)-2,6-diazaspiro[3.4]octane-2-carboxylate **8***

A total of 0.443 g of N,N′-carbonyldiimidazole (2.73 mmol, 1.25 equiv.) and 0.343 g of 5-nitro-2-furoic acid (2.18 mmol, 1 equiv.) were dissolved in dry DMF (10 mL). The mixture was stirred for 20 min following the adding of a solution of 0.757 g of compound **6** (2.73 mmol, 1.25 equiv.) and 0.882 g of triethylamine (8.73 mmol, 4 equiv.) in 5 mL of dry DMF to the mixture. The mixture was stirred for 1 h, then the reaction mixture was poured into water (30 mL) and extracted with ethyl acetate (3 × 20 mL). The organic phase was washed with a 5% K_2_CO_3_ solution (10 mL) and brine. The organic phase was dried over Na_2_SO_4_ and evaporated in vacuo. The residue was recrystallized from EtOH. Yield: 0.618 g (70%), colorless oil. LCMS (ESI), *m*/*z*: 433.4 [M+H]^+^.

##### General Procedure for the Synthesis of Compounds **3a**–**e**

A total of 0.68 g (1.68 mmol) of compound **8** was dissolved in a 1:1 mixture of CF_3_COOH:CH_2_Cl_2_ (2 mL). The mixture was stirred for 1 h at 0 °C. After that, the mixture was evaporated in vacuo. The residue (8-(4-methyl-4*H*-1,2,4-triazol-3-yl) -6-(5-nitro-2-furoyl)-2,6-diazaspiro[3.4]octane trifluoroacetate **8a**) was used in the next step without further purification.

To a mixture of 0.45 g (1 mmol) of compound **8a** and 0.23 g of triethylamine (2.28 mmol) in 25 mL of CH_2_Cl_2_, a solution of 1.5 mmol of the corresponding sulfonyl chloride in 15 mL of CH_2_Cl_2_ was added dropwise while cooling the mixture to 0 °C. The reaction mixture was allowed to reach room temperature and stirred for 18 h. Then, the mixture was poured in water (30 mL), and the organic phase was separated and washed with 20 mL of 5% (aq) K_2_CO_3_. The organic phase was then separated again, dried over anhydrous Na_2_SO_4_, filtered, and concentrated in vacuo. The residue was purified by column chromatography on silica gel eluting with 0% → 10% methanol in CH_2_Cl_2_ to give the title compound.

*2-(methylsulfonyl)-8-(4-methyl-4H-1,2,4-triazol-3-yl)-6-(5-nitro-2-furoyl)-2,6-diazaspiro[3.4]octane **3a***

Yield: 0.051 g (41%) from 0.135 g of **8a**, white solid, Mp 183–184 °C. ^1^H-NMR (300 MHz, DMSO–d_6_) mixture of rotamers δ 8.46 (s, 1H), 7.80 (d, *J* = 3.9 Hz, 0,5H), 7.75 (d, *J* = 3.9 Hz, 0,5H), 7.42 (d, *J* = 3.9 Hz, 0,5H), 7.36 (d, *J* = 3.9 Hz, 0,5H), 4.52–4.29 (m, 1H), 4.24–3.82 (m, 8H), 3.70 (d, *J* = 4.6 Hz, 3H), 2.94 (s, 3H); ^13^C-NMR (75 MHz, DMSO–d_6_) mixture of rotamers δ 156.01, 152.36, 151.97, 148.17, 145.67, 118.11, 113.46, 60.18, 59.66, 56.33, 56.10, 55.65, 55.43, 51.06, 50.62, 42.69, 38.08, 33.26, 32.88, 30.99; LCMS (ESI), *m*/*z*: 411.4 [M+H]^+^; HRMS (ESI) *m*/*z* calcd for C_15_H_19_N_6_O_6_S [M+H^+^] 411.1087 Da, found 411.1073 Da.

*2-(ethylsulfonyl)-8-(4-methyl-4H-1,2,4-triazol-3-yl)-6-(5-nitro-2-furoyl)-2,6-diazaspiro[3.4]octane **3b***

Yield: 0.052 g (40%) from 0.135 g of **8a**, white solid, Mp 185–186 °C. ^1^H-NMR (300 MHz, DMSO–d_6_) mixture of rotamers δ 8.45 (s, 1H), 7.80 (d, *J* = 3.9 Hz, 0,5H), 7.74 (d, *J* = 3.9 Hz, 0,5H), 7.42 (d, *J* = 3.9 Hz, 0,5H), 7.35 (d, *J* = 3.9 Hz, 0,5H), 4.57–4.41 (m, 1H), 4.27–3.83 (m, 8H), 3.70 (d, *J* = 5.1 Hz, 3H), 3.06 (m, 2H), 1.22–1.12 (m, 3H); ^13^C-NMR (75 MHz, DMSO–d_6_) mixture of rotamers δ 156.00, 155.93, 152.31, 151.94, 151.79, 148.21, 145.64, 118.05, 113.40, 113.32, 59.60, 59.09, 56.24, 56.01, 55.34, 55.08, 51.03, 50.59, 42.95, 42.92, 42.66, 38.11, 30.97, 30.92, 7.76; LCMS (ESI), *m*/*z*: 425.4 [M+H]^+^; HRMS (ESI) *m*/*z* calcd for C_16_H_21_N_6_O_6_S [M+H^+^] 425.1243 Da, found 425.1250 Da.

*2-(isopropylsulfonyl)-8-(4-methyl-4H-1,2,4-triazol-3-yl)-6-(5-nitro-2-furoyl)-2,6-diazaspiro[3.4]octane **3c***

Yield: 0.052 g (41%) from 0.130 g of **8a**, white solid, Mp 178–179 °C. ^1^H-NMR (300 MHz, DMSO–d_6_) mixture of rotamers δ 8.46 (s, 1H), 7.79 (d, *J* = 3.9 Hz, 0.5H), 7.74 (d, *J* = 3.9 Hz, 0.5H), 7.42 (d, *J* = 3.9 Hz, 0.5H), 7.36 (d, *J* = 3.9 Hz, 0.5H), 4.45–4.27 (m, 1H), 4.14–3.79 (m, 8H), 3.71 (d, *J* = 5.1 Hz, 3H), 3.23–3.09 (m, 1H), 1.14 (dd, *J* = 6.9, 2.8 Hz, 6H); ^13^C NMR (75 MHz, DMSO-d_6_) mixture of rotamers δ 155.58, 155.51, 151.82, 151.44, 147.83, 145.25, 145.21, 117.67, 117.62, 113.00, 112.93, 59.45, 58.89, 55.78, 55.59, 55.51, 55.21, 52.17, 52.13, 50.64, 50.17, 37.70, 30.58, 15.94; LCMS (ESI), *m*/*z*: 439.5 [M+H^+^]^+^; HRMS (ESI) *m*/*z* calcd for C_17_H_23_N_6_O_6_S [M+H^+^] 439.1399 Da, found 439.1373 Da

*2-(cyclopropylsulfonyl)-8-(4-methyl-4H-1,2,4-triazol-3-yl)-6-(5-nitro-2-furoyl)-2,6-diazaspiro[3.4]octane **3d***

Yield: 0.049 g (38%) from 0.130 g of **8a**, white solid, Mp 171–172 °C. ^1^H-NMR (300 MHz, DMSO–d_6_) mixture of rotamers δ 8.46 (s, 1H), 7.79 (d, *J* = 3.9 Hz, 0,5H), 7.75 (d, *J* = 3.9 Hz, 0,5H), 7.42 (d, *J* = 3.9 Hz, 0,5H), 7.36 (d, *J* = 3.9 Hz, 0,5H), 4.53–4.30 (m, 1H), 4.25–3.79 (m, 8H), 3.70 (d, *J* = 4.8 Hz, 3H), 2.70–2.60 (m, 1H), 1.02–0,82 (m, 4H); ^13^C-NMR (75 MHz, DMSO–d_6_) mixture of rotamers δ 155.98, 155.94, 152.25, 151.88, 151.80, 151.72, 148.19, 148.17, 145.71, 118.14, 113.48, 113.39, 59.98, 59.47, 56.38, 56.15, 55.72, 55.50, 51.05, 50.63, 43.09, 37.99, 31.10, 30.99, 24.19, 23.86, 4.30, 4.25; LCMS (ESI), *m*/*z*: 437.4 (M+H+); HRMS (ESI) *m*/*z* calcd for C_17_H_21_N_6_O_6_S [M+H]^+^ 437.1243 Da, found 437.1235 Da

*2-(butylsulfonyl)-8-(4-methyl-4H-1,2,4-triazol-3-yl)-6-(5-nitro-2-furoyl)-2,6-diazaspiro[3.4]octane **3e***

Yield: 0.035 g (26%) from 0.130 g of **8a**, white solid, Mp 189–190 °C. ^1^H-NMR (300 MHz, DMSO–d_6_) mixture of rotamers δ 8.46 (c, 1H), 7.79 (d, *J* = 3.9 Hz, 0,5H), 7.74 (d, *J* = 3.9 Hz, 0,5H), 7.42 (d, *J* = 3.9 Hz, 0,5H), 7.36 (d, *J* = 3.9 Hz, 0,5H), 4.48–4.27 (m, 1H), 4.13–3.78 (m, 8H), 3.70 (d, *J* = 4.7 Hz, 3H), 3.07–2.98 (m, 2H), 1.66–1.49 (m, 2H), 1.45–1.28 (m, 2H), 0.95–0.82 (m, 3H); ^13^C-NMR (75 MHz, DMSO–d_6_) mixture of rotamers δ 155.99, 151.89, 151.71, 148.23, 145.64, 118.05, 113.41, 113.33, 59.44, 56.27, 56.04, 55.26, 55.11, 51.06, 50.59, 47.62, 47.27, 42.90, 38.06, 30.98, 24.96, 21.28, 13.84; LCMS (ESI), *m*/*z*: 453.5 [M+H]^+^; HRMS (ESI) *m*/*z* calcd for C_18_H_25_N_6_O_6_S [M+H^+^] 453.1556 Da, found 453.1569 Da

#### 4.1.3. Synthesis of 4 and 5 Series

##### General Procedure for the Synthesis of Compounds **4a**–**d** and **5a**–**c**

A total of 0.443 g of N,N′-carbonyldiimidazole (2.73 mmol, 1.25 equiv.) and 0.343 g of 5-nitro-2-furoic acid (2.18 mmol, 1 equiv.) were dissolved in dry DMF (10 mL). The mixture was stirred for 20 min, after a solution of the corresponding amine compound (**9** for **4** series or **10** for **5** series) (2.73 mmol, 1.25 equiv.) in 5 mL of dry DMF was added to the mixture. The mixture was stirred for 1 h, then the resultant mixture was poured into water (30 mL) and extracted with ethyl acetate (3 × 20 mL). The organic phase was washed with a 5% K_2_CO_3_ solution (10 mL) and brine. The organic phase was dried over Na_2_SO_4_ and evaporated in vacuo. The residue was purified by column chromatography on silica gel eluting with 0% → 5% methanol in CH_2_Cl_2_ to give the title compound.

*6-(5-nitro-2-furoyl)-8-(4-propyl-4H-1,2,4-triazol-3-yl)-6-azaspiro[3.4]octane **4a***

Yield: 0.110 g (53%) from 0.160 g of **9a**, white solid, Mp 181–182 °C. ^1^H-NMR (300 MHz, DMSO–d_6_) mixture of rotamers δ 8.50 (s, 1H), 7.78 (d, *J* = 3.9 Hz, 0,5H), 7.74 (d, *J* = 3.9 Hz, 0,5H), 7.42 (d, *J* = 3.9 Hz, 0,5H), 7.37 (d, *J* = 3.9 Hz, 0,5H), 4.31–4.13 (m, 1H), 4.07–3.68 (m, 6H), 2.13–1.99 (m, 2H), 1.91–1.65 (m, 6H), 0.89 (td, 3H); ^13^C-NMR (75 MHz, DMSO-d6) δ 155.94, 152.89, 152.44, 148.41, 144.33, 118.00, 113.40, 58.05, 57.81, 50.86, 48.84, 46.40, 45.34, 32.38, 24.04, 15.81, 11.10; LCMS (ESI), *m*/*z*: 360.4 [M+H]^+^; HRMS (ESI) *m*/*z* calcd for C_17_H_22_N_5_O_4_ [M+H^+^] 360.1672 Da, found 360.1685 Da

*4-(4-methyl-4H-1,2,4-triazol-3-yl)-2-(5-nitro-2-furoyl)-2-azaspiro[4.4]nonane **4b***

Yield: 0.198 g (51%) from 0.300 g of **9b**, white solid, Mp 163–164 °C. ^1^H-NMR (300 MHz, DMSO–d_6_) mixture of rotamers δ 8.39 (s, 1H), 7.77 (d, *J* = 3.9 Hz, 0,5H), 7.74 (d, *J* = 3.9 Hz, 0,5H), 7.40 (t, *J* = 4.1 Hz, 1H), 4.31–4.07 (m, 1H), 4.03–3.75 (m, 2H), 3.73–3.62 (m, 4H), 3.60–3.46 (m, 1H), 1.71–1.42 (m, 7H), 1.40–1.29 (m, 1H); ^13^C-NMR (75 MHz, DMSO–d_6_) mixture of rotamers δ 155.95, 155.90, 153.88, 153.42, 151.75, 148.52, 148.42, 145.11, 118.10, 117.94, 113.42, 113.40, 58.18, 57.88, 54.11, 52.25, 51.90, 51.42, 41.41, 36.95, 36.61, 31.57, 30.95, 24.64, 24.50, 24.33; LCMS (ESI), *m*/*z*: 346.4 [M+H]^+^; HRMS (ESI) *m*/*z* calcd for C_16_H_20_N_5_O_4_ [M+H^+^] 346.1515 Da, found 346.1519 Da

*4-(4-methyl-4H-1,2,4-triazol-3-yl)-2-(5-nitro-2-furoyl)-2-azaspiro[4.5]decane **4c***

Yield: 0.040 g (41%) from 0.078 g of **9c**, white solid, Mp 168–169 °C. ^1^H-NMR (300 MHz, DMSO–d_6_) mixture of rotamers δ 8.40 (s, 1H), 7.78 (d, *J* = 3.9 Hz, 0,5H), 7.74 (d, *J* = 3.9 Hz, 0,5H), 7.44 (dd, *J* = 3.9 Hz, 0,5H), 7.39 (d, *J* = 3.9 Hz, 0,5H), 4.33–4.11 (m, 1H), 4.04–3.82 (m, 2H), 3.65 (d, *J* = 4.3 Hz, 3H), 3.60–3.40 (m, 2H), 1.67–1.04 (m, 10H); ^13^C-NMR (75 MHz, DMSO–d_6_) mixture of rotamers δ 156.06, 153.05, 152.61, 151.73, 148.57, 148.43, 145.22, 118.03, 117.94, 113.34, 54.95, 54.48, 50.85, 50.56, 46.90, 44.02, 43.20, 35.00, 34.93, 31.05, 30.16, 25.75, 23.35, 23.10, 22.92, 22.85; LCMS (ESI), *m*/*z*: 360.4 [M+H]^+^; HRMS (ESI) *m*/*z* calcd for C_17_H_22_N_5_O_4_ [M+H^+^] 360.1672 Da, found 360.1687 Da

*4-(4-methyl-4H-1,2,4-triazol-3-yl)-2-(5-nitro-2-furoyl)-8-oxa-2-azaspiro[4.5]decane **4d***

Yield: 0.097 g (47%) from 0.165 g of **9c**, white solid, Mp 177–178 °C. ^1^H-NMR (300 MHz, DMSO–d_6_) mixture of rotamers δ 8.42 (s, 1H), 7.79 (d, *J* = 3.9 Hz, 0,5H), 7.75 (d, *J* = 3.9 Hz, 0,5H), 7.46 (d, *J* = 3.9 Hz, 0,5H), 7.39 (d, *J* = 3.9 Hz, 0,5H), 4.34–4.00 (m, 2H), 3.99–3.81 (m, 2H), 3.81–3.56 (m, 6H), 3.55–3.37 (m, 2H), 1.70–1.61 (m, 2H), 1.52–1.33 (m, 1H), 3.55–3.37 (m, 1H); ^13^C-NMR (75 MHz, DMSO–*d_6_*) mixture of rotamers δ 156.11, 156.06, 152.71, 152.28, 148.54, 148.37, 145.29, 118.06, 117.91, 113.35, 64.52, 64.42, 64.35, 54.43, 54.03, 50.57, 50.25, 44.65, 42.89, 41.84, 34.76, 31.05, 30.61, 30.55; LCMS (ESI), *m*/*z*: 362.4 [M+H]^+^; HRMS (ESI) *m*/*z* calcd for C_16_H_20_N_5_O_5_ [M+H^+^] 362.1464 Da, found 362.1453 Da

*4-(cyclopropylmethyl)-3-{[1-(5-nitro-2-furoyl)azetidin-3-yl]methyl}-4H-1,2,4-triazole **5a***

Yield: 0.025 g (16%) from 0.110 g of **10a**, white solid, Mp 159–160 °C. ^1^H-NMR (300 MHz, DMSO–d_6_) δ 8.50 (s, 1H), 7.77 (d, *J* = 3.9 Hz, 1H), 7.32 (d, *J* = 3.9 Hz, 1H), 4.71 (t, *J* = 8.8 Hz, 1H), 4.35–4.17 (m, 2H), 3.90–3.73 (m, 3H), 3.31–3.07 (m, 3H), 1.25–1.10 (m, 1H), 0.60–0.46 (m, 2H), 0.43–0.32 (m, 2H); ^13^C-NMR (75 MHz, DMSO–d_6_) δ 156.65, 151.82, 151.68, 147.98, 144.18, 117.28, 113.51, 58.03, 54.07, 48.09, 28.15, 27.55, 11.55, 4.15. LCMS (ESI), *m*/*z*: 332.3 [M+H]^+^; HRMS (ESI) *m*/*z* calcd for C_15_H_18_N_5_O_4_ [M+H^+^] 332.1359 Da, found 332.1364 Da

*4-isopropyl-3-{[1-(5-nitro-2-furoyl)azetidin-3-yl]methyl}-4H-1,2,4-triazole **5b***

Yield: 0.018 g (13%) from 0.100 g of **10b**, white solid, Mp 164–165 °C. ^1^H-NMR (300 MHz, DMSO–d_6_) δ 8.60 (s, 1H), 7.77 (d, *J* = 3.9 Hz, 1H), 7.32 (d, *J* = 3.9 Hz, 1H), 4.72 (t, *J* = 8.8 Hz, 1H), 4.46–4.18 (m, 3H), 3.84 (dd, *J* = 10.4, 5.5 Hz, 1H), 3.30–3.15 (m, 1H), 3.11 (d, *J* = 6.9 Hz, 2H), 1.39 (d, *J* = 6.7 Hz, 6H); ^13^C-NMR (75 MHz, DMSO–*d_6_*) δ 156.65, 151.81, 151.13, 148.01, 141.68, 117.26, 113.50, 58.07, 54.09, 46.73, 28.36, 27.57, 23.27; LCMS (ESI), *m*/*z*: 320.3 [M+H]^+^; HRMS (ESI) *m*/*z* calcd for C_14_H_18_N_5_O_4_ [M+H^+^] 320.1359 Da, found 320.1374 Da

*4-cyclopentyl-3-{[1-(5-nitro-2-furoyl)azetidin-3-yl]methyl}-4H-1,2,4-triazole **5c***

Yield: 0.056 g (35%) from 0.120 g of **10c**, white solid, Mp 170–171 °C. ^1^H-NMR (300 MHz, DMSO–d_6_) δ 8.57 (s, 1H), 7.77 (d, *J* = 3.9 Hz, 1H), 7.32 (d, *J* = 3.9 Hz, 1H), 4.71 (t, *J* = 8.6 Hz, 1H), 4.48 (p, *J* = 7.2 Hz, 1H), 4.34–4.17 (m, 2H), 3.83 (dd, *J* = 9.8, 5.7 Hz, 1H), 3.22 (q, *J* = 7.2 Hz, 1H), 3.12 (d, *J* = 6.8 Hz, 2H), 2.20–2.04 (m, 2H), 1.85–1.60 (m, 6H); ^13^C-NMR (75 MHz, DMSO–*d_6_*) δ 156.65, 151.84, 148.00, 141.98, 117.27, 113.51, 58.05, 55.55, 54.08, 33.15, 28.56, 27.57, 23.67; LCMS (ESI), *m*/*z*: 346.4 [M+H]^+^; HRMS (ESI) *m*/*z* calcd for C_16_H_20_N_5_O_4_ [M+H^+^] 346.1515 Da, found 346.1528 Da

### 4.2. Antibacterial Activity

All synthesized compounds were tested against the *Mycobacterium tuberculosis* H37Rv drug-sensitive strain, relative to isoniazid and ethambutol, which were served as a positive control. The *MTb* H37Rv strain (originating from the Institute of Hygiene and Epidemiology in Prague, 1976) was obtained on 7 August 2013 from the Federal Scientific Center for Expertise of Medical Products (RF Ministry of Health Care). The lyophilized strain was inoculated on Löwenstein–Jensen growth medium. The minimal inhibitory concentration (MIC) of the compounds was determined using a REMA (resazurin microtiter plate assay) [35]. Testing was performed as described previously [36].

Testing was also performed against the following microorganisms: *Enterococcus faecalis* (ATCC 29812), *Staphylococcus aureus* (ATCC 25912), *Klebsiella pneumoni*ae (ATCC 19882), *Acinetobacter baumannii* (948^®^, patient-derived strain from the Pasteur Institute, own collection), *Pseudomonas aeruginosa* (ATCC 27853), and *Enterobacter cloacae* (ATCC 13047) for all synthesized compounds and ciprofloxacin (used as a positive control). MICs were determined using serial broth dilutions [37]. All measurements were carried out in triplicate.

### 4.3. Molecular Modeling

#### 4.3.1. Protein Models Used for Calculations

The following protein models from the PDB database were used in the calculations: *Mtb* deazaflavin-dependent nitroreductase Ddn–3R5W [38], *Mtb* arylamine-N-acetyltransferase TBNAT–4BGF [39], and *Mtb* transcriptional regulatory repressor protein EthR–5NZ0 [40].

For the following proteins, the models were predicted by the neural network AlphaFold [41]: there are *S. aureus* azoreductase (AzoR-AF-Q99X11-F1), *S. aureus* oxygen-insensitive NADPH nitroreductase A (NfsA-AF-Q6GJR6-F1), and *S. aureus* oxygen-insensitive NAD(P)H nitroreductase B (NfsB-AF-A0A653YAV7-F1).

*S. aureus* dihydrofolate reductase DHFR–3SRR [42] and phosphoglycerate kinase Pgk–4DG5 [20] can be considered side targets, so we included them in our calculations.

#### 4.3.2. Protein and Small Molecule Preparation

All proteins were preprocessed before calculations using the Protein Prepwizard tool from the Schrodinger suite [43]. During preprocessing, errors such as missing amino acid sidechains, incorrect protonation states, missing hydrogens, incorrect bond orders, angles, etc. were fixed, solvent (water) molecules were deleted, and restrained minimization for X-ray structures was performed [44].

The ligand geometry was generated by the LigPrep module. All molecular modeling operations were performed in the OPLS4 force field [45]. Schrödinger Suite 2022-4 [43] was used for calculations.

#### 4.3.3. Induced-Fit Docking Procedure

The docking grid box was calculated on the basis of reference ligand coordinates and size (grid placement on complex ligand centroid, maximum grid side size is 12 Å). If a reference ligand is absent, the centroid coordinates of residues in the protein active cavity are used (in the case of Ddn protein, the centroid between FMN and Tyr65/130/133/136 is used). For each ligand, 20 docking solutions were calculated. The best-fitting binding pose was selected by comparison with reference ligand positioning in the protein active site (if it was present in the PDB files) or by orientation towards interacting aminoacids and flavine mononucleotide. All ligands present in the used PDB models were redocked for docking quality validation and reference-scoring function value calculations.

Binding pose clustering is also a significant parameter that indicates the quality of ligand binding. Furthermore, the observed cluster must replicate the pharmacophore characteristics of the reference ligands (if they are present). This value is indicated by stars in parentheses. Three stars represent a clustered docking solution with a root mean square deviation (RMSD) of less than 1.5 Å in more than 60% of cases, two stars indicate a clustered docking solution with an RMSD of less than 3 Å in 40–60% of cases, and one star indicates a clustered docking solution with an RMSD of less than 40% or no clustering.

#### 4.3.4. MM-GBSA Calculations

For each ligand binding pose, Gibbs free energy (ΔG) was calculated with the presence of an implicit solvent. Calculations performed with the use of the MM-GBSA method [46]. The point of interest is strain energy value distribution, which indicates the most strained protein–ligand interactions. This parameter can explain the absence of correct interactions with proteins and, as follows, decreased activity.

## 5. Conclusions

Structural modification of the lead molecule **1** did not improve its anti-TB activity because the parent compound appears to bind better to its preferred target than others. However, due to the privileged scaffold structure, analogues of compound **1** exhibit superior activity against another target of another pathogen, demonstrating the superiority of privileged structure-based drug development. Of course, calculations are not enough to determine the exact target. An experiment with enzyme test systems is needed, the results of which we plan to publish separately. Nevertheless, substances **2b** and **5c** can definitely be used as leads for the development of a selective agent against *S. aureus*.

## Figures and Tables

**Table 1:** Minimal inhibitory concentration (MIC, µg/mL) of active compounds and positive control antibiotics against *MTb* and *S. aureus*; nt—not tested. The MIC values are mean values from three different assays (errors were in the range of ±5–10% of the reported values). The MIC values less than the positive control are highlighted in green. Iz = isoniazid; Eth = ethambutol; Cip = ciprofloxacin.

Compd	1	2b	4a	4c	5a	5b	5c	Iz	Eth	Cip
*MTb*	0.031	>100	3.1	100	12.5	25	6.2	0.025–0.05	0.78–2.0	nt
*S. aureus*	>100	0.171	2.95	2.3	>100	>100	0.19	nt	nt	1.25

**Table 2:** Results of molecular docking of the investigated compounds into the active sites of the most likely targets associated with antibiotics of the nitrofuran class; stars indicate the quality of the binding pose, where ***—good, **—fair, *—low. Coloring in Target columns: green—correlated with experiment; yellow—fair correlation.

GlideScore (kcal/mol) and Binding Quality (Stars)									
	Species	*Mtb*			*S. aureus*				
Compd	Target	Ethr	TBNAT	Ddn	NfsA	NfsB	DHFR	AzoR	Pgk
**1**		−6.33 (*)	−5.14 (*)	−8.47 (***)	−4.73 (*)	−6.61 (**)	−7.21 (***)	−6.72 (*)	−6.79 (***)
**2b**		−4.49 (*)	−5.33 (*)	−5.63 (*)	−6.62 (***)	−7.93 (***)	−7.58 (***)	−8.03 (***)	−6.10 (***)
**4a**		n/a	−4.55 (*)	−5.55 (**)	−5.94 (**)	−7.31 (***)	−6.23 (*)	−6.77 (**)	−6.36 (***)
**4c**		n/a	−3.93 (*)	−5.37 (*)	−6.44 (***)	−6.25 (***)	−7.15 (***)	−6.79 (***)	−6.73 (***)
**5a**		−8.92 (*)	−4.41 (*)	−5.51 (**)	−5.44 (***)	−6.30 (*)	−6.55 (***)	−6.65 (**)	−5.54 (***)
**5b**		−9.12 (*)	−5.12 (*)	−5.42 (**)	−5.70 (***)	−5.62 (*)	−5.78 (*)	−6.60 (*)	−6.15 (***)
**5c**		−9.36 (*)	−5.79 (*)	−5.43 (**)	−6.54 (***)	−7.81 (***)	−6.71 (***)	−7.72 (***)	−5.08 (*)

## Data Availability

Data are contained within the article and Supplementary Materials.

## Supplementary Materials

The following supporting information can be downloaded at: https://www.mdpi.com/article/10.3390/ijms26010207/s1.
