# Supplementary material for: You Win Some, You Lose Some: Modifying the Molecular Periphery of Nitrofuran-Tagged Diazaspirooctane Reshapes Its Antibacterial Activity Profile
Source: Int J Mol Sci. 2024 Dec 29;26(1):207. doi: 10.3390/ijms26010207 (PMC11720470; doi:10.3390/ijms26010207)
Supplement: Supplementary file 1 [file ijms-26-00207-s001.zip › ijms-3370550-supplementary.pdf]

Supporting Information

You Win Some, You Lose Some: Modifying the Molecular Periphery of Nitrofuran-Tagged Diazaspirooctane Reshapes Its Antibacterial Activity Profile

Lyubov Vinogradova [1], Kristina Komarova [1], Alexey Lukin [1], Maxim Zhuravlev [1], Dmitry Deniskin [1], Anastasia Poliakova [1], Mikhail Chudinov [1,*], Maxim Gureev [2], Marine Dogonadze [3], Tatiana Vinogradova [3], Elizaveta Rogacheva [4], Lyudmila Kraeva [4], Yuri Porozov [5,6] and Viktor Korzhikov-Vlakh [7,*]

1 Lomonosov Institute of Fine Chemical Technologies, MIREA—Russian Technological University, Moscow 119454, Russia; vlv010599@yandex.ru (L.V.); kristinka-komarova.1999@mail.ru (K.K.); alex-look@yandex.ru (A.L.); max.2903@mail.ru (M.Z.); deniskin.02d@mail.ru (D.D.); polyakova.a.e@edu.mirea.ru (A.P.)
2 Institute of Cytology, Russian Academy of Sciences, Saint Petersburg 194064, Russia; max_technik@mail.ru
3 Saint-Petersburg State Research Institute of Phthisiopulmonology of the Ministry of Healthcare of the Russian Federation, Saint Petersburg 191036, Russia; marine-md@mail.ru (M.D.); vinogradova@spbniif.ru (T.V.)
4 Pasteur Institute of Epidemiology and Microbiology, Saint Petersburg 197101, Russia; elizvla@yandex.ru (E.R.); lykraeva@yandex.ru (L.K.)
5 Laboratory of Angiopathology, The Institute of General Pathology and Pathophysiology, Moscow 125315, Russia; yporozov@hse.ru
6 Advitam Laboratory, 11108 Belgrade, Serbia
7 Department of Medical Chemistry, Institute of Chemistry, Saint Petersburg State University, Saint Petersburg 199034, Russia
* Correspondence: chudinov@mirea.ru (M.C.); v.korzhikov-vlakh@spbu.ru (V.K.-V.)

NMR spectra of synthesized compounds

6-(methylsulfonyl)-8-(4-methyl-4H-1,2,4-triazol-3-yl) -2-(5-nitro-2-furoyl)-2,6-diazaspiro[3.4]octane 1 3

6-(isopropylsulfonyl)-8-(4-methyl-4H-1,2,4-triazol-3-yl)-2-(5-nitro-2-furoyl)-2,6-diazaspiro[3.4]octane 2a 4

6-(cyclopropylsulfonyl)-8-(4-methyl-4H-1,2,4-triazol-3-yl)-2-(5-nitro-2-furoyl)-2,6- diazaspiro[3.4]octane 2b 5

N,N-dimethyl-8-(4-methyl-4H-1,2,4-triazol-3-yl)-2-(5-nitro-2-furoyl)-2,6-diazaspiro[3.4] octane-6-sulfonamide 2c ... 6

6-[(4-methylphenyl)sulfonyl]-8-(4-methyl-4H-1,2,4-triazol-3-yl)-2-(5-nitro-2-furoyl)-2,6- diazaspiro[3.4]octane 2d ... 7

6-[(2,4-difluorophenyl)sulfonyl]-8-(4-methyl-4H-1,2,4-triazol-3-yl)-2-(5-nitro-2-furoyl)- 2,6-diazaspiro[3.4]octane 2e 8

6-[(4-fluorophenyl)sulfonyl]-8-(4-methyl-4H-1,2,4-triazol-3-yl)-2-(5-nitro-2-furoyl)-2,6- diazaspiro[3.4]octane 2f 9

6-(benzylsulfonyl)-8-(4-methyl-4H-1,2,4-triazol-3-yl)-2-(5-nitro-2-furoyl)-2,6-diazaspiro [3.4]octane 2g 10

6-[(3,5-difluorophenyl)sulfonyl]-8-(4-methyl-4H-1,2,4-triazol-3-yl)-2-(5-nitro-2-furoyl)- 2,6-diazaspiro[3.4]octane 2h 11

6-[(3-fluoro-4-methylphenyl)sulfonyl]-8-(4-methyl-4H-1,2,4-triazol-3-yl)-2-(5-nitro-2- furoyl)-2,6-diazaspiro[3.4]octane 2i 12

6-[(3-chlorophenyl)sulfonyl]-8-(4-methyl-4H-1,2,4-triazol-3-yl)-2-(5-nitro-2-furoyl)-2,6- diazaspiro[3.4]octane 2j 13

2-(methylsulfonyl)-8-(4-methyl-4H-1,2,4-triazol-3-yl)-6-(5-nitro-2-furoyl)-2,6-diazaspiro [3.4]octane 3a 14

2-(ethylsulfonyl)-8-(4-methyl-4H-1,2,4-triazol-3-yl)-6-(5-nitro-2-furoyl)-2,6-diazaspiro [3.4]octane 3b 15

2-(isopropylsulfonyl)-8-(4-methyl-4H-1,2,4-triazol-3-yl)-6-(5-nitro-2-furoyl)-2,6-diazaspiro[3.4]octane 3c 16

2-(cyclopropylsulfonyl)-8-(4-methyl-4H-1,2,4-triazol-3-yl)-6-(5-nitro-2-furoyl)-2,6- diazaspiro[3.4]octane 3d 17

2-(butylsulfonyl)-8-(4-methyl-4H-1,2,4-triazol-3-yl)-6-(5-nitro-2-furoyl)-2,6-diazaspiro [3.4]octane 3e 18

6-(5-nitro-2-furoyl)-8-(4-propyl-4H-1,2,4-triazol-3-yl)-6-azaspiro[3.4]octane 4a 19

4-(4-methyl-4H-1,2,4-triazol-3-yl)-2-(5-nitro-2-furoyl)-2-azaspiro[4.4]nonane 4b 20

4-(4-methyl-4H-1,2,4-triazol-3-yl)-2-(5-nitro-2-furoyl)-2-azaspiro[4.5]decane 4c 21

4-(4-methyl-4H-1,2,4-triazol-3-yl)-2-(5-nitro-2-furoyl)-8-oxa-2-azaspiro[4.5]decane 4d 22

4-(cyclopropylmethyl)-3-{[1-(5-nitro-2-furoyl)azetidin-3-yl]methyl}-4H-1,2,4-triazole 5a 23

4-isopropyl-3-{[1-(5-nitro-2-furoyl)azetidin-3-yl]methyl}-4H-1,2,4-triazole 5b 24

4-cyclopentyl-3-{[1-(5-nitro-2-furoyl)azetidin-3-yl]methyl}-4H-1,2,4-triazole 5c 25

LCMS data files of target compounds (see SI1.zip) 26

6-(methylsulfonyl)-8-(4-methyl-4H-1,2,4-triazol-3-yl) -2-(5-nitro-2-furoyl)-2,6-diazaspiro[3.4]octane ***1***

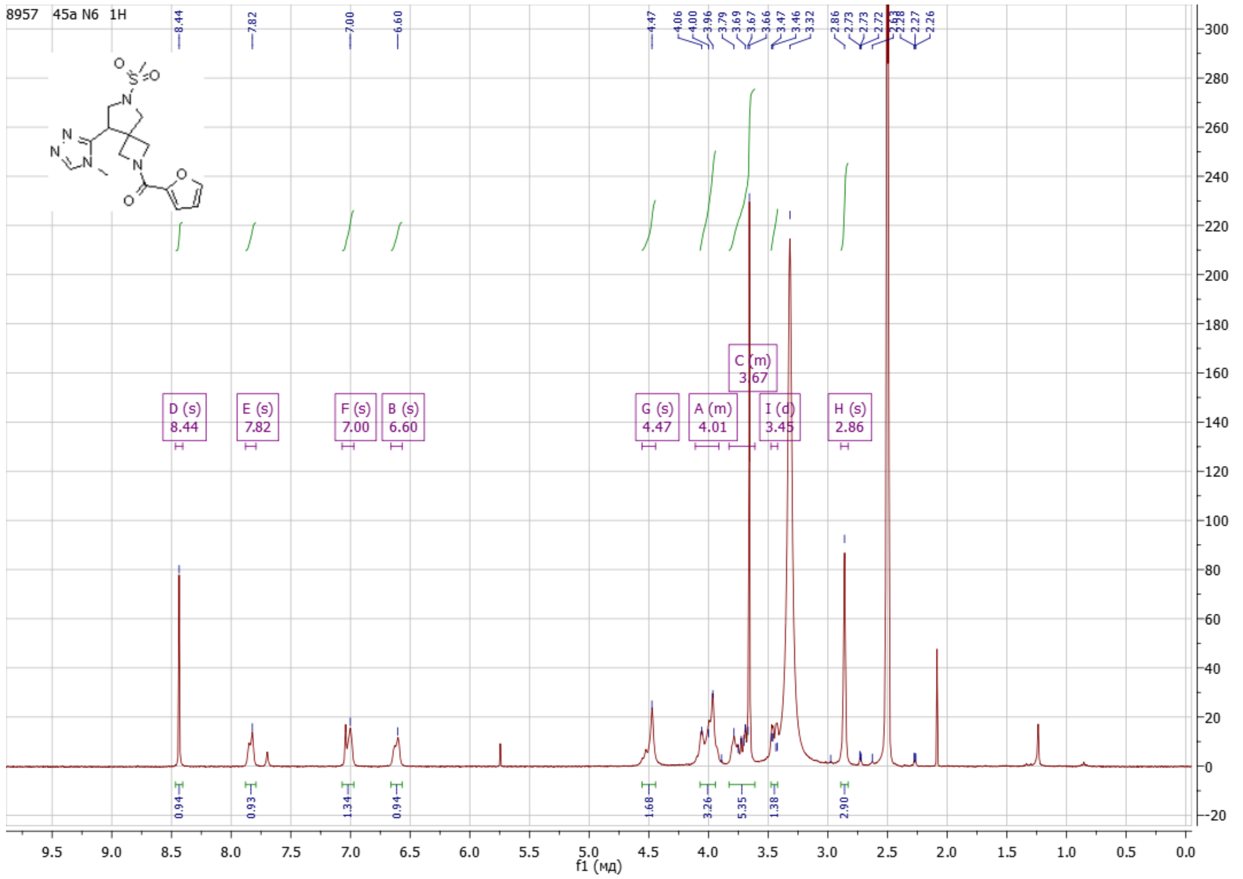

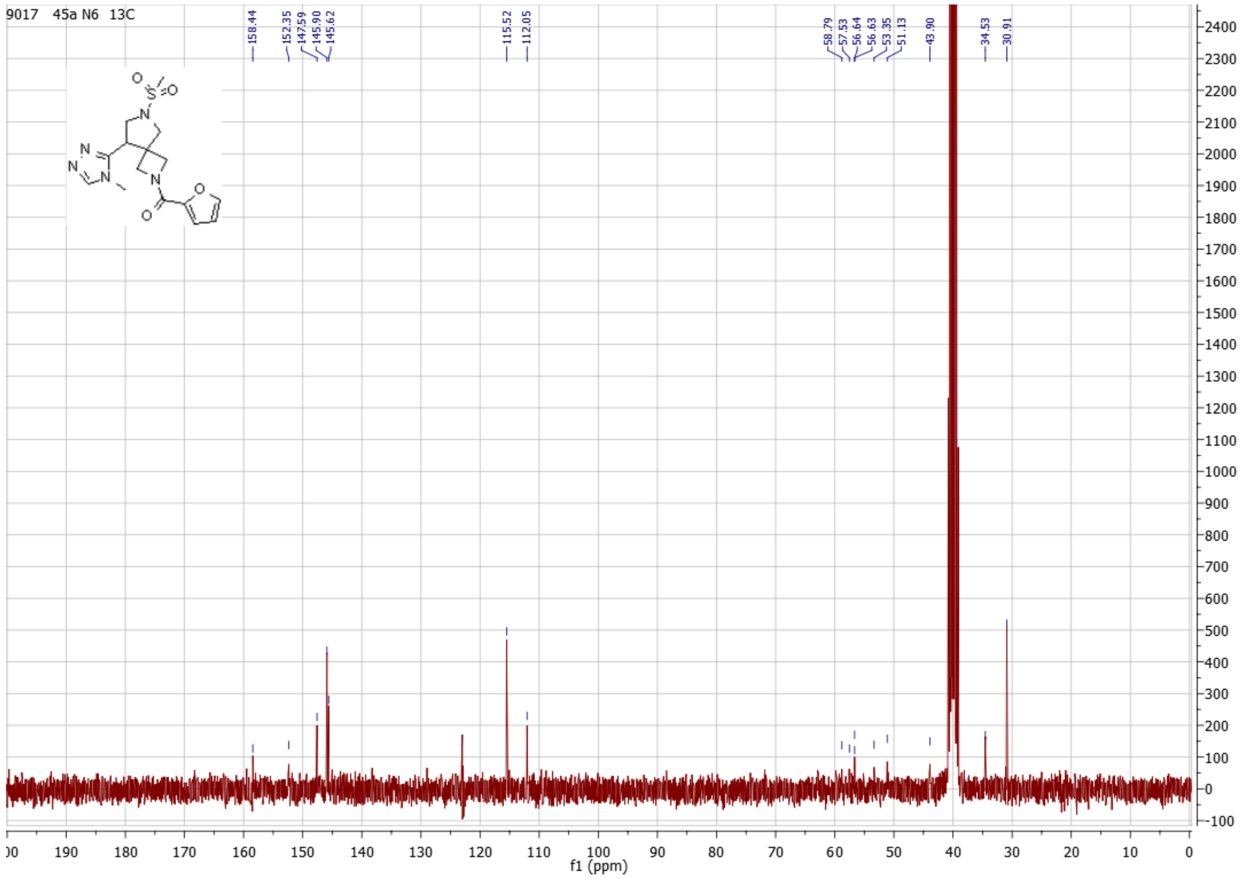

*6-(isopropylsulfonyl)-8-(4-methyl-4H-1,2,4-triazol-3-yl)-2-(5-nitro-2-furoyl)-2,6-diazaspiro[3.4]octane **2a***

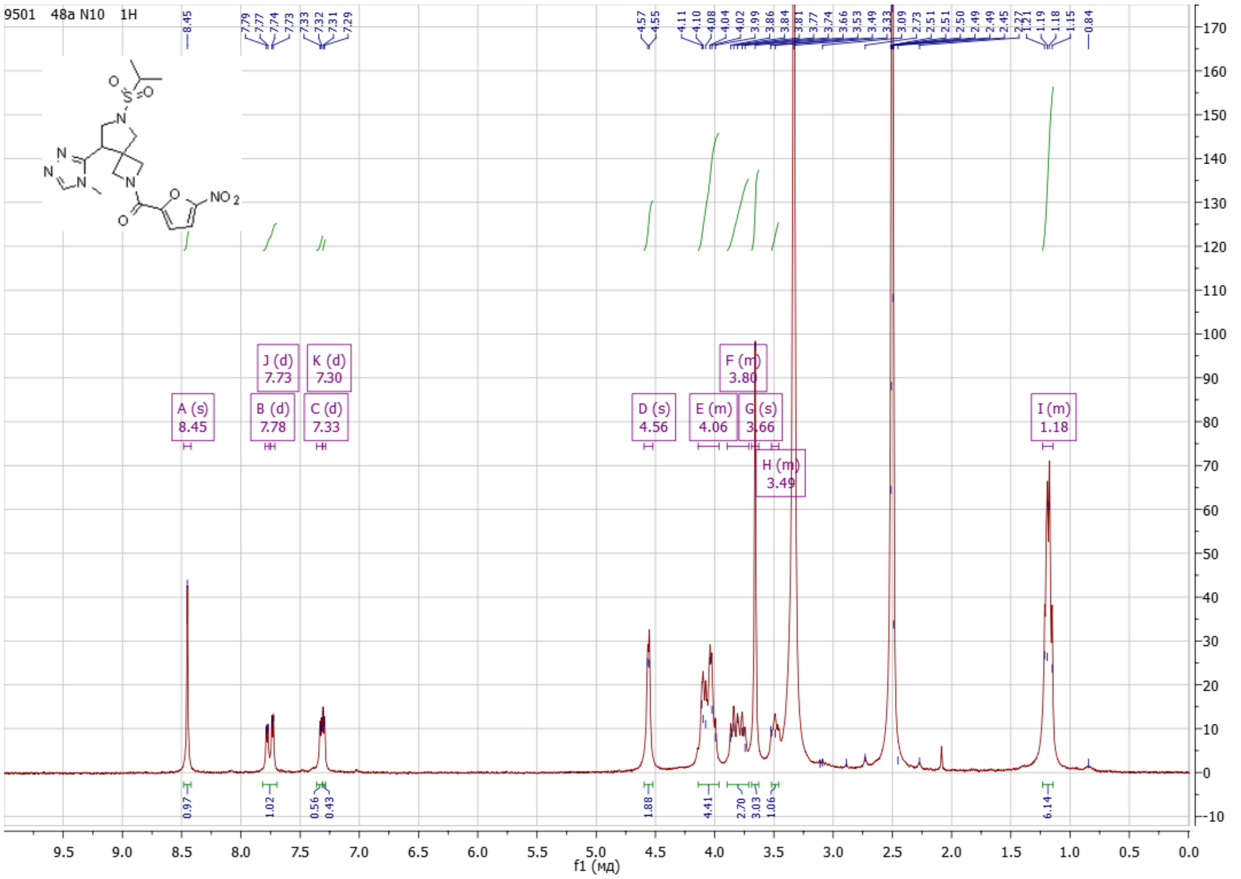

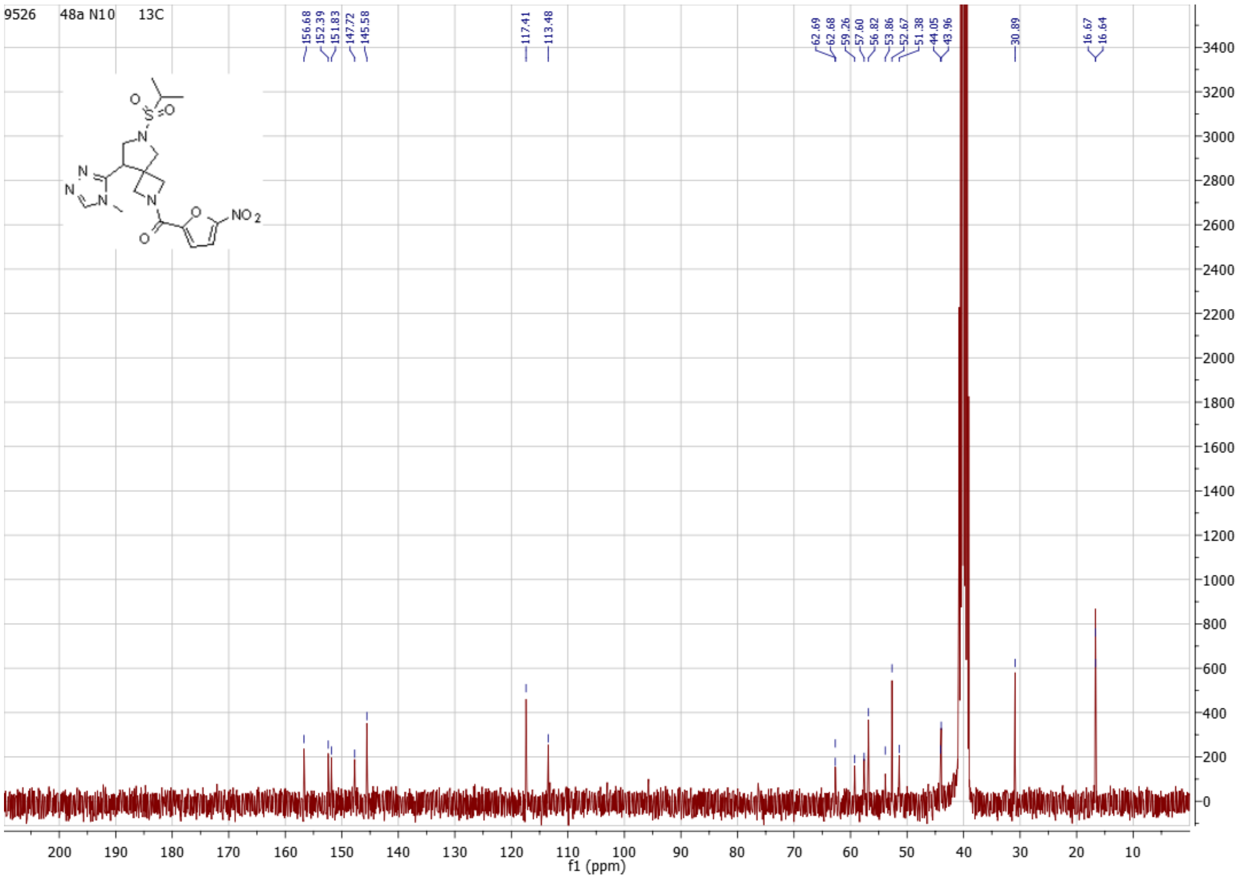

6-(cyclopropylsulfonyl)-8-(4-methyl-4H-1,2,4-triazol-3-yl)-2-(5-nitro-2-furoyl)-2,6- diazaspiro[3.4]octane ***2b***

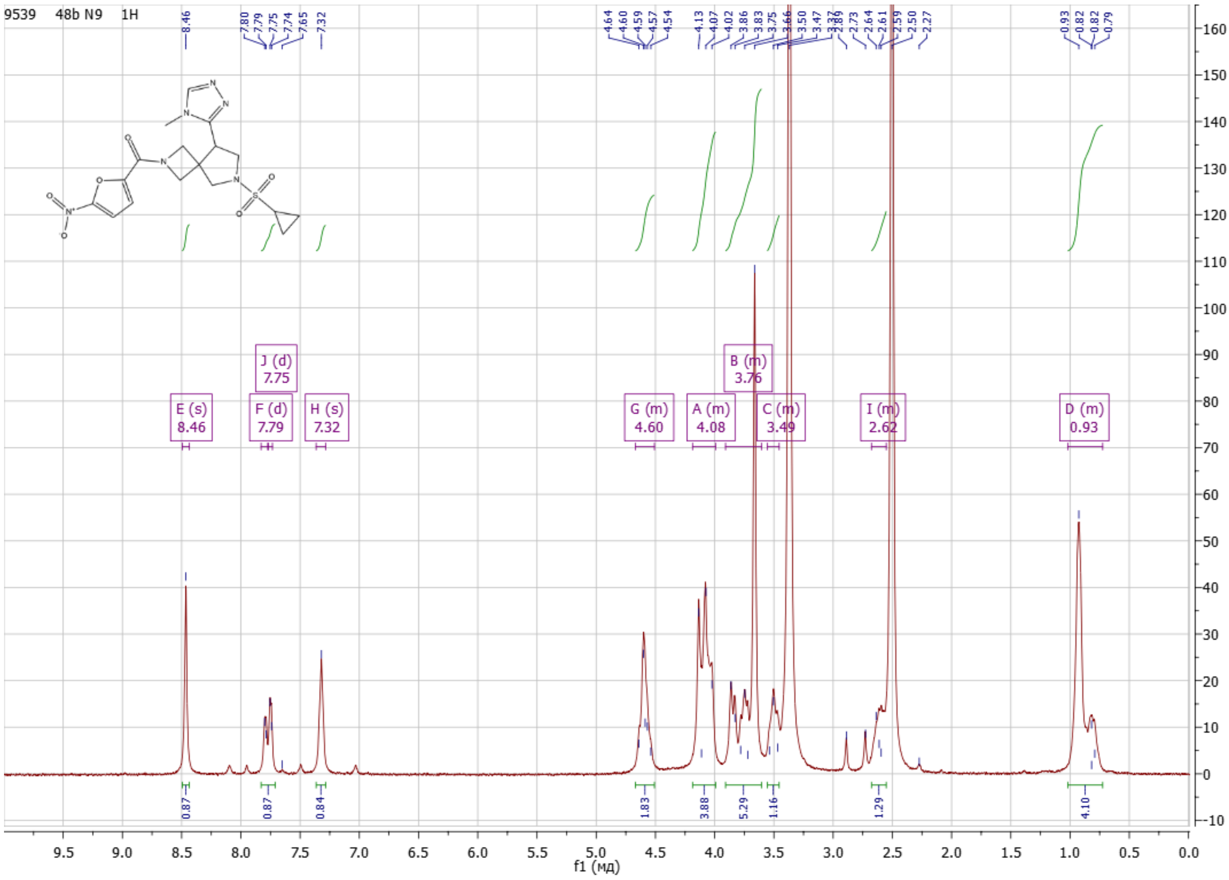

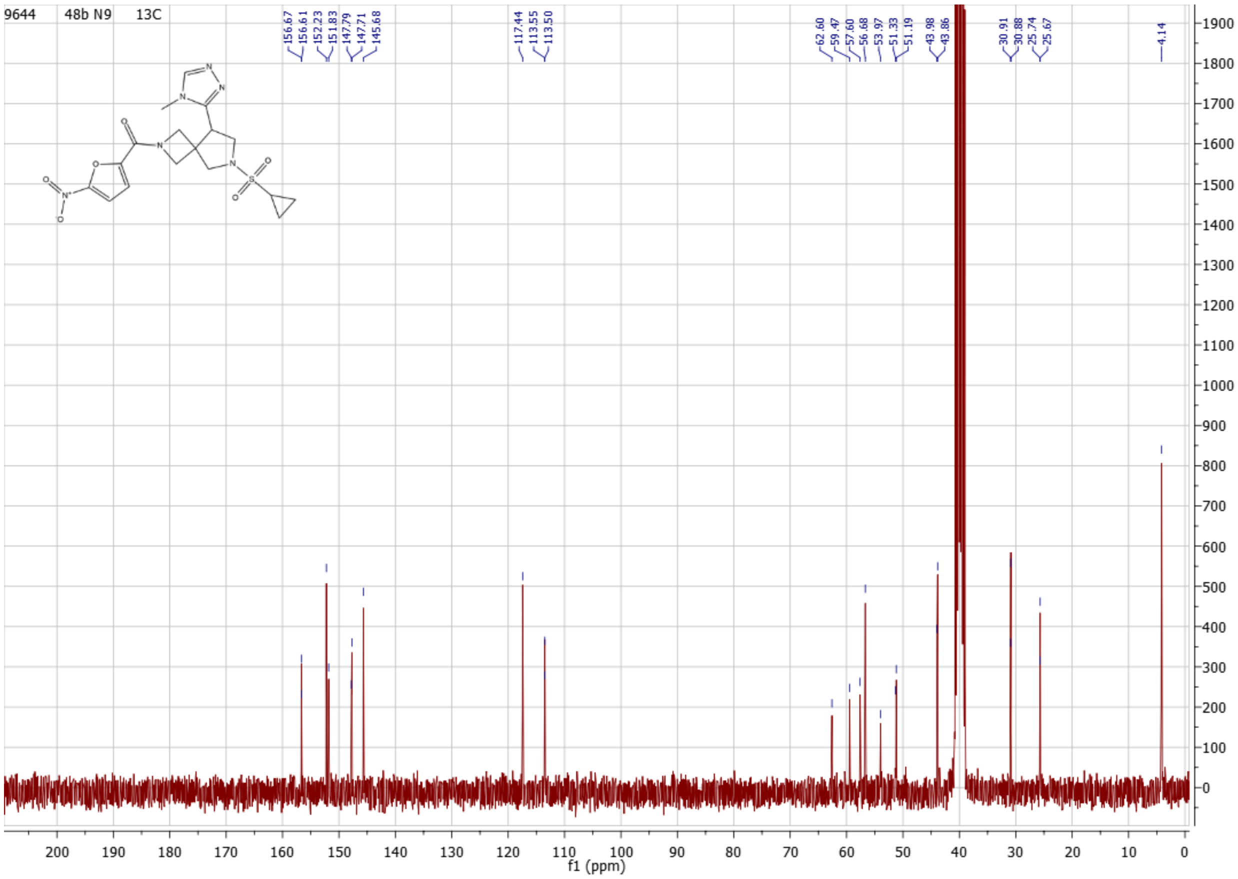

N,N-dimethyl-8-(4-methyl-4H-1,2,4-triazol-3-yl)-2-(5-nitro-2-furoyl)-2,6-diazaspiro[3.4] octane-6-sulfonamide ***2c***

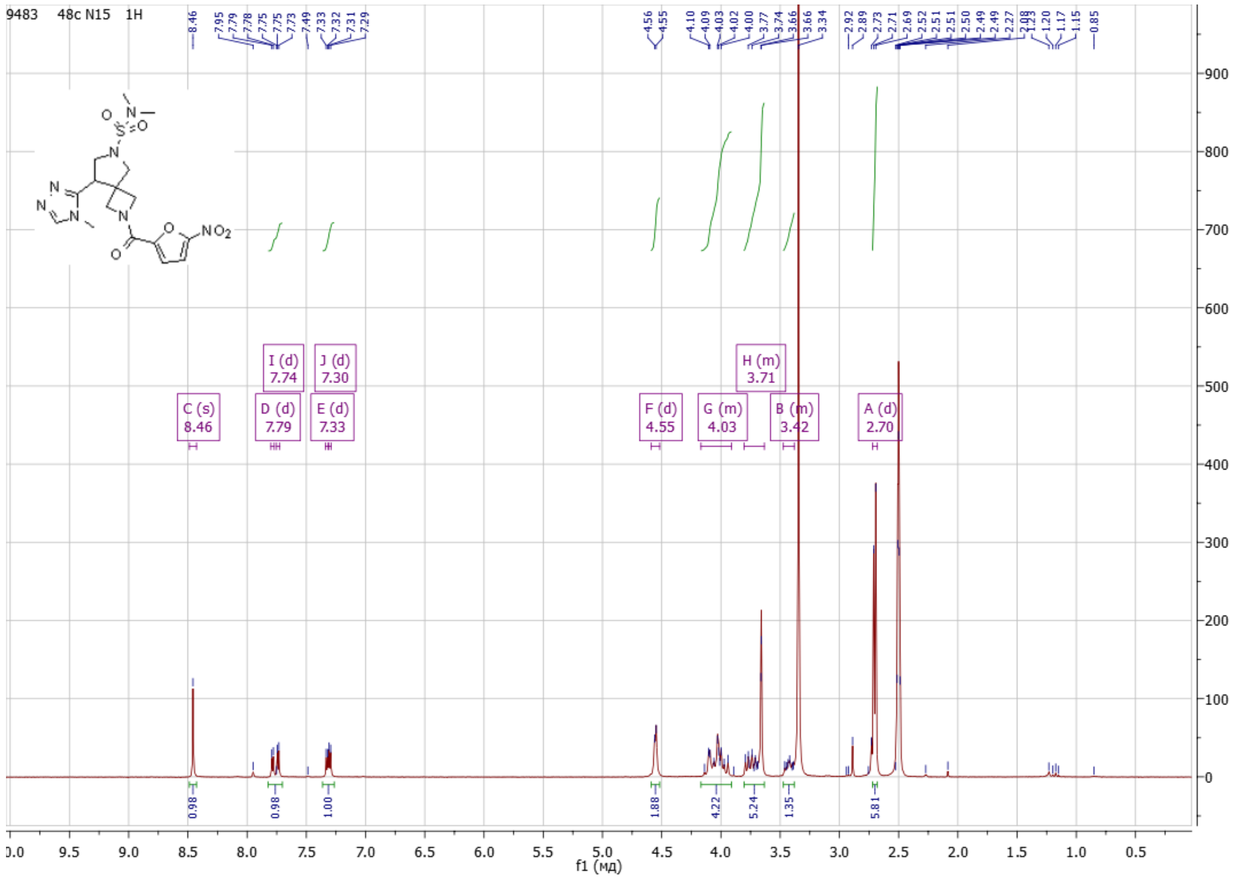

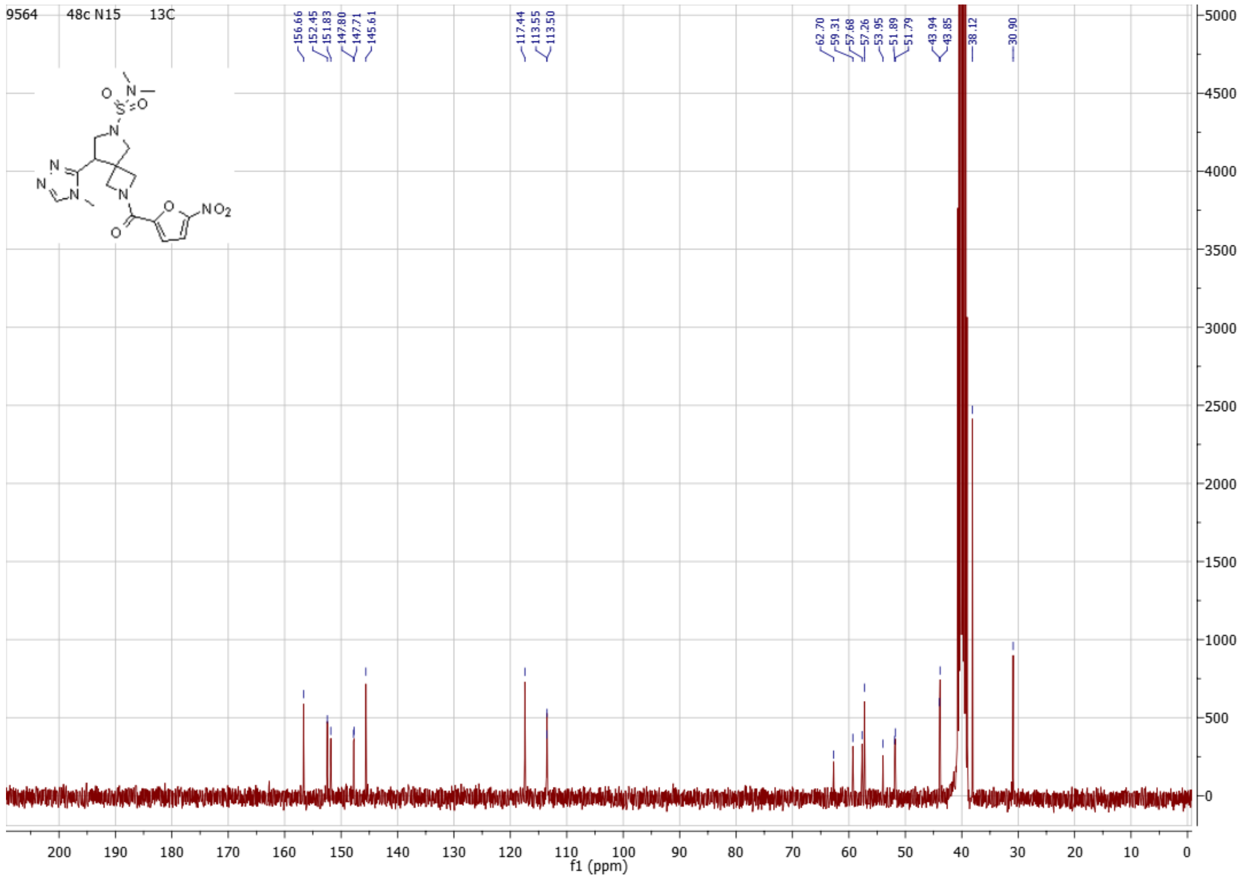

6-[(4-methylphenyl)sulfonyl]-8-(4-methyl-4H-1,2,4-triazol-3-yl)-2-(5-nitro-2-furoyl)-2,6- diazaspiro[3.4]octane ***2d***

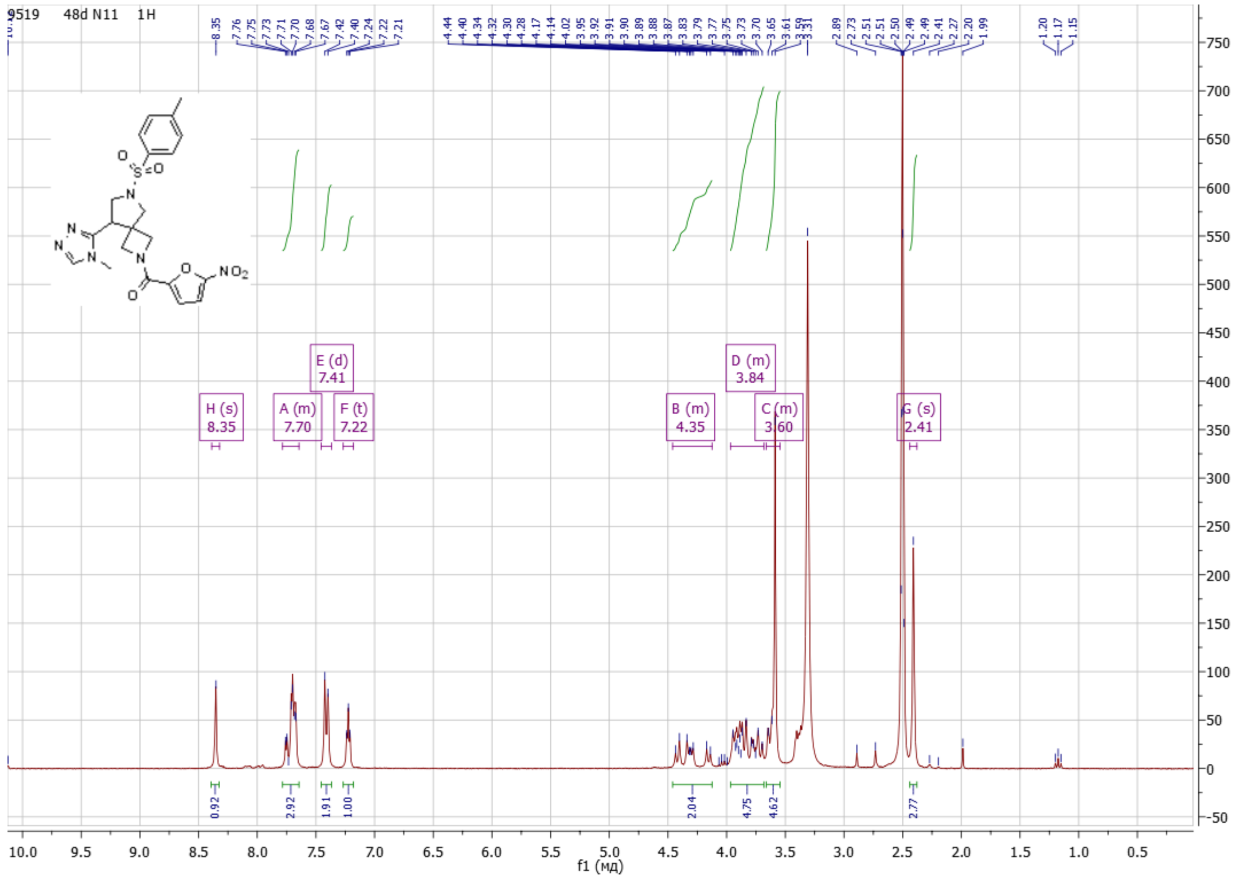

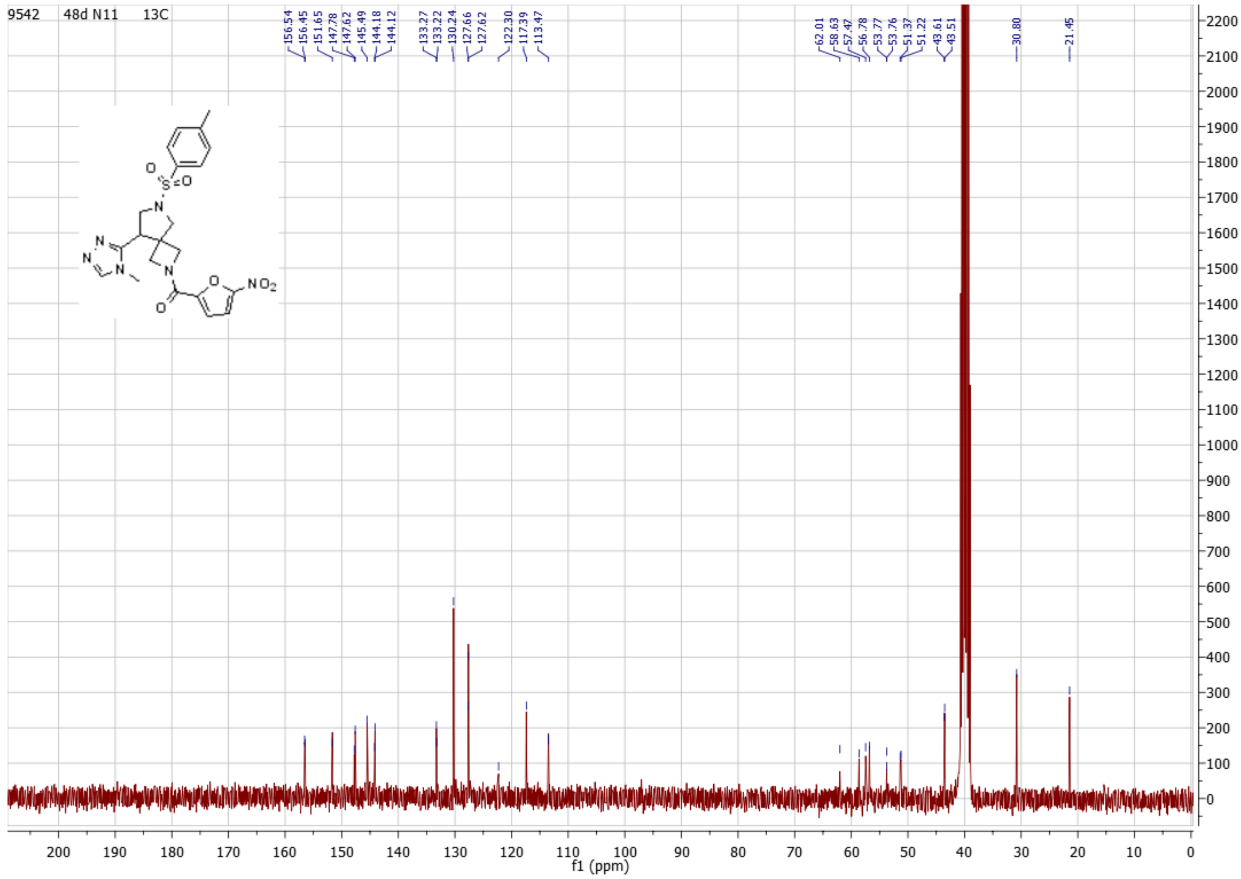

6-[(2,4-difluorophenyl)sulfonyl]-8-(4-methyl-4H-1,2,4-triazol-3-yl)-2-(5-nitro-2-furoyl)- 2,6-diazaspiro[3.4]octane ***2e***

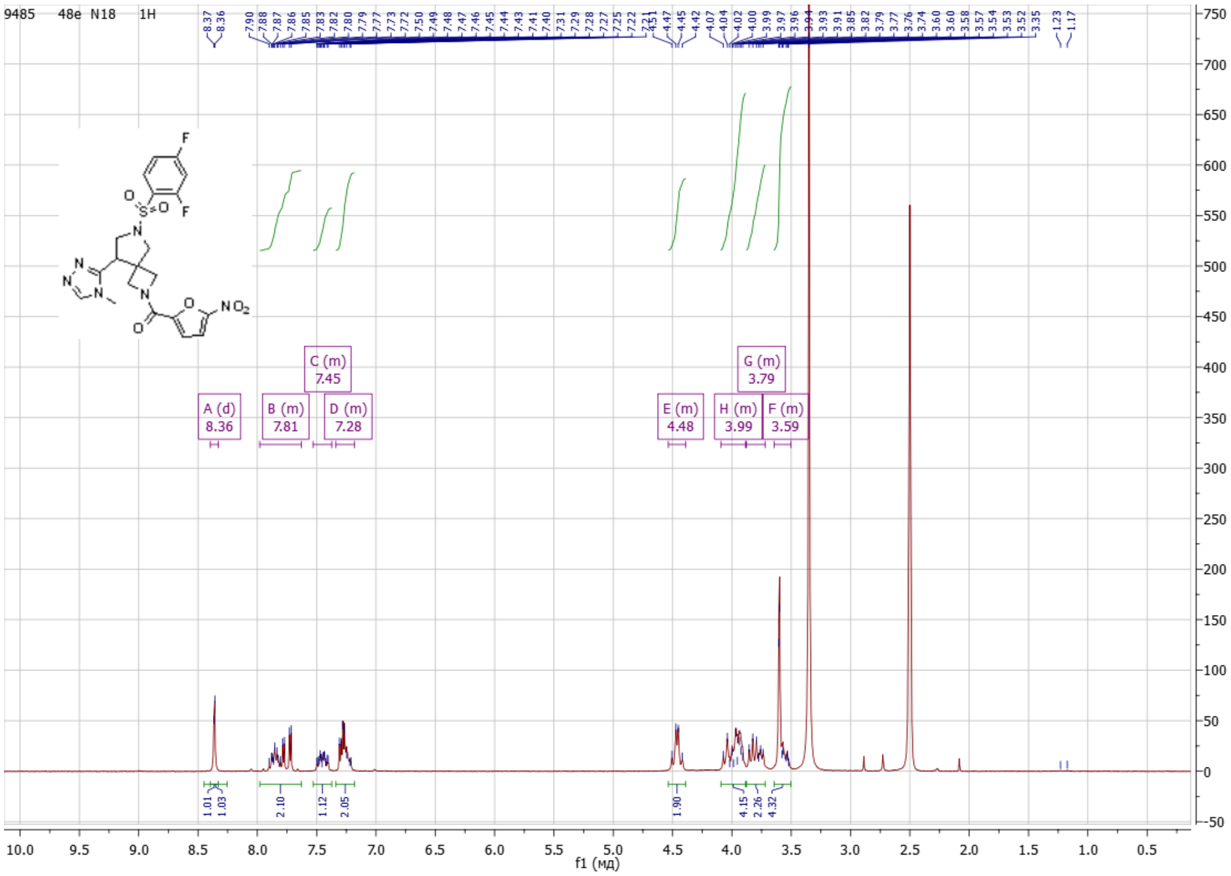

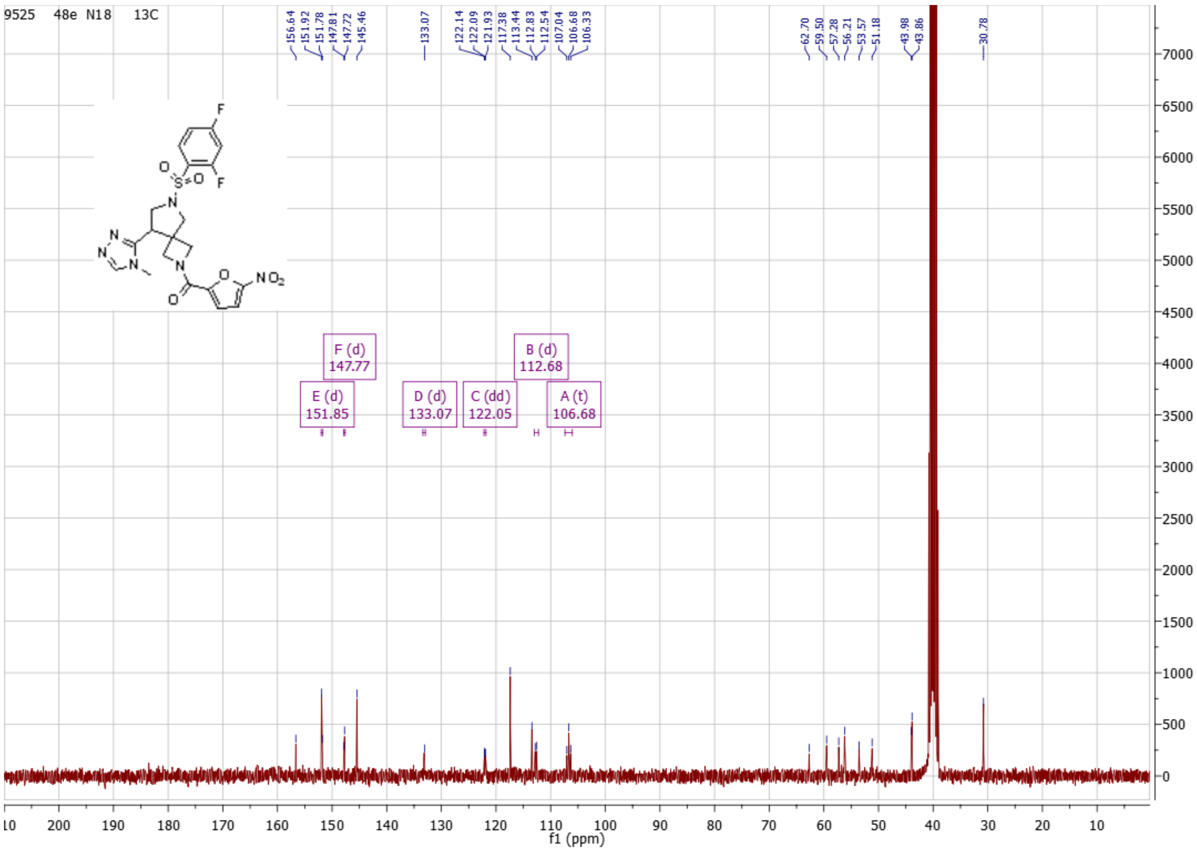

6-[(4-fluorophenyl)sulfonyl]-8-(4-methyl-4H-1,2,4-triazol-3-yl)-2-(5-nitro-2-furoyl)-2,6- diazaspiro[3.4]octane ***2f***

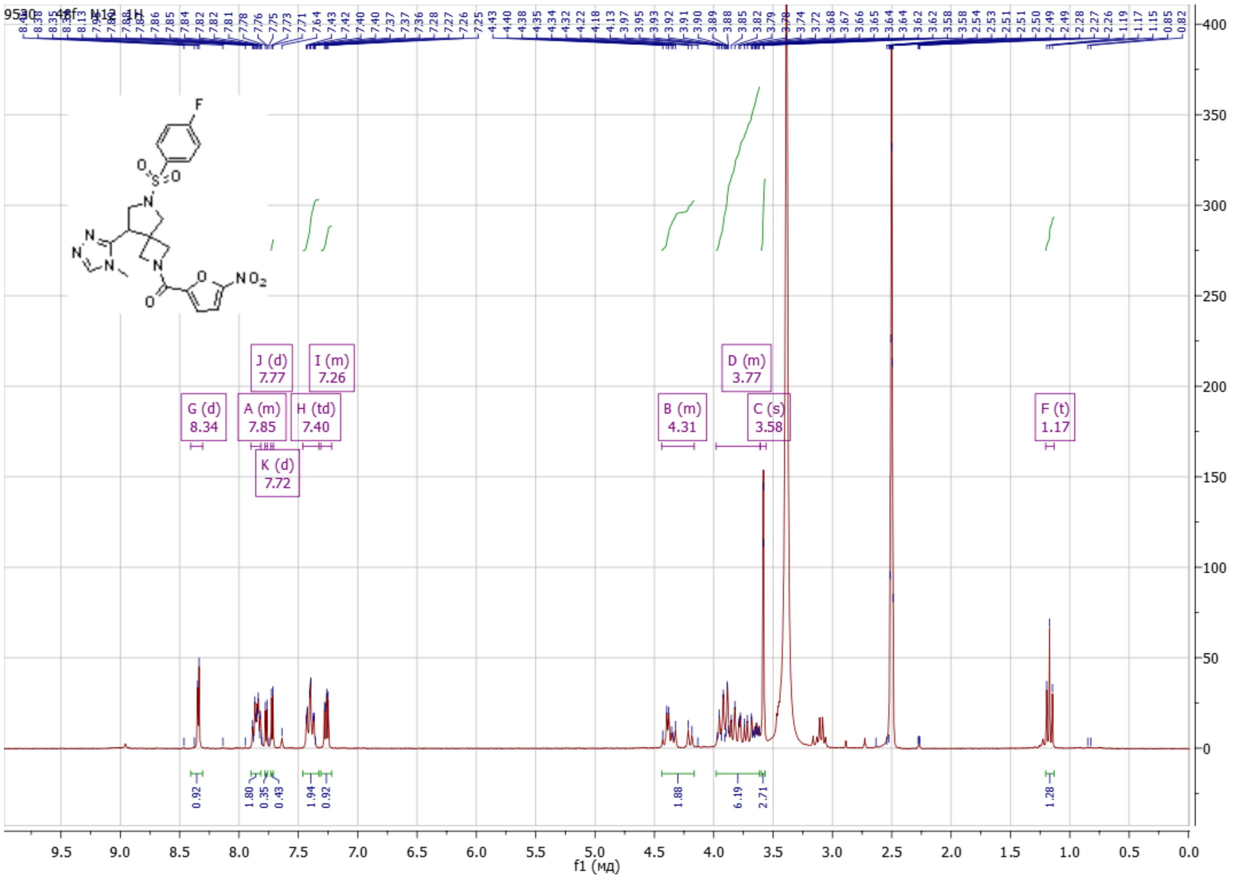

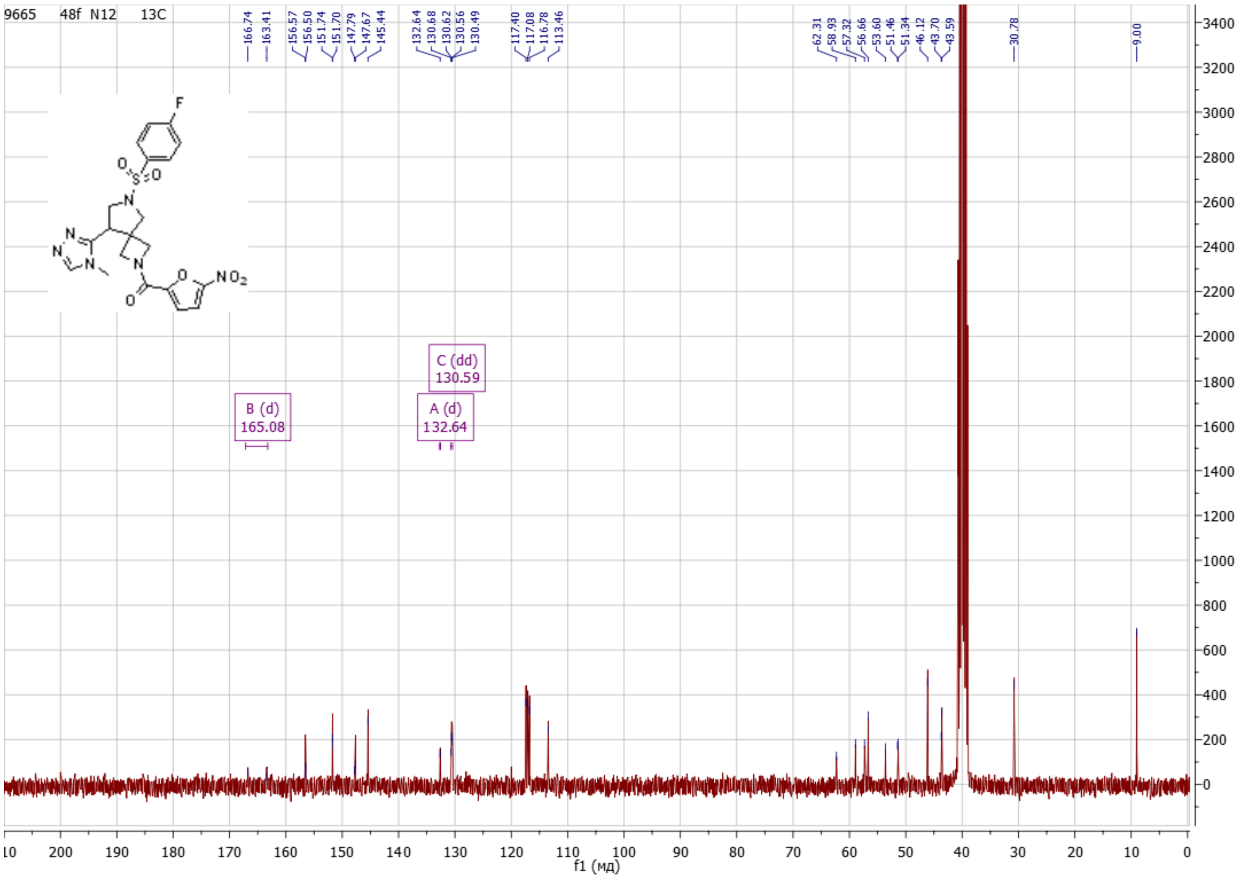

6-(benzylsulfonyl)-8-(4-methyl-4H-1,2,4-triazol-3-yl)-2-(5-nitro-2-furoyl)-2,6-diazaspiro [3.4]octane ***2g***

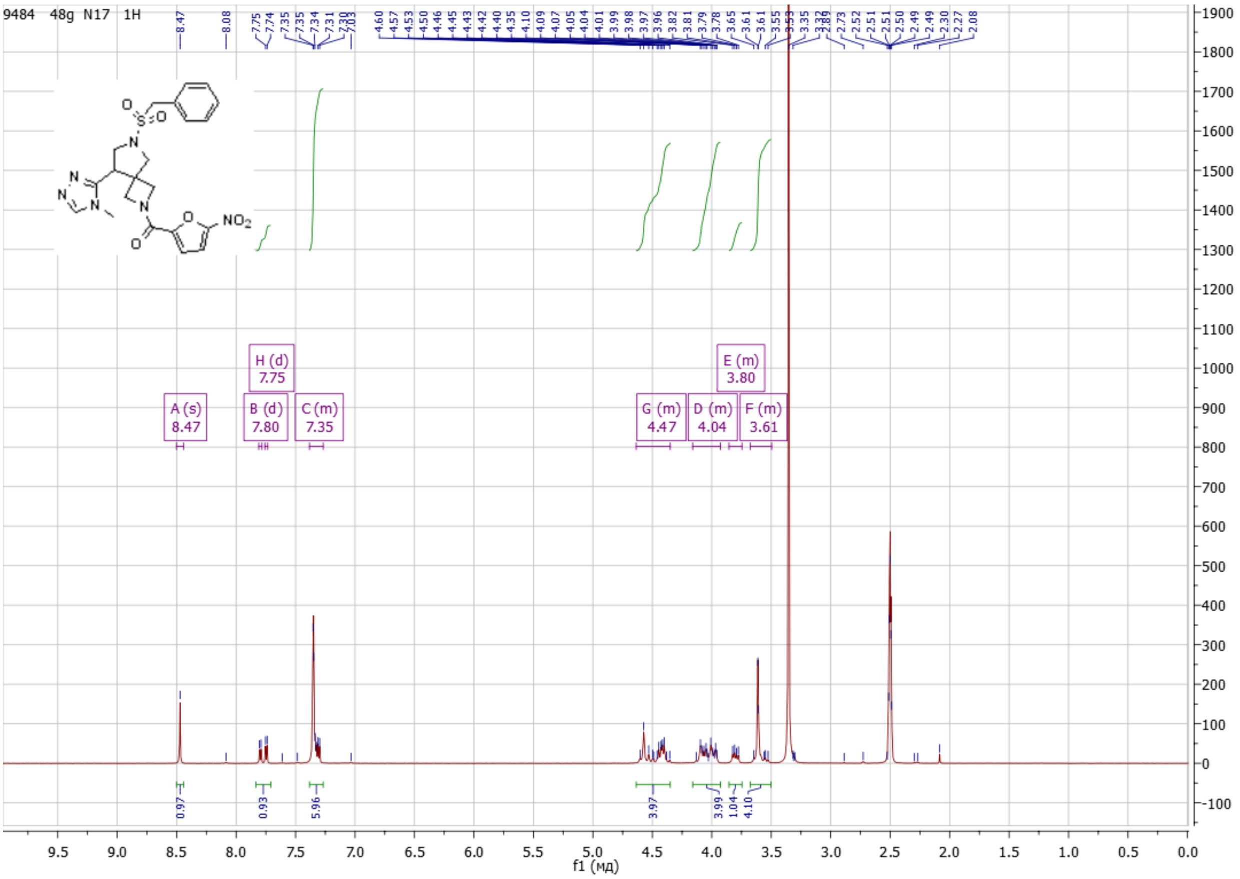

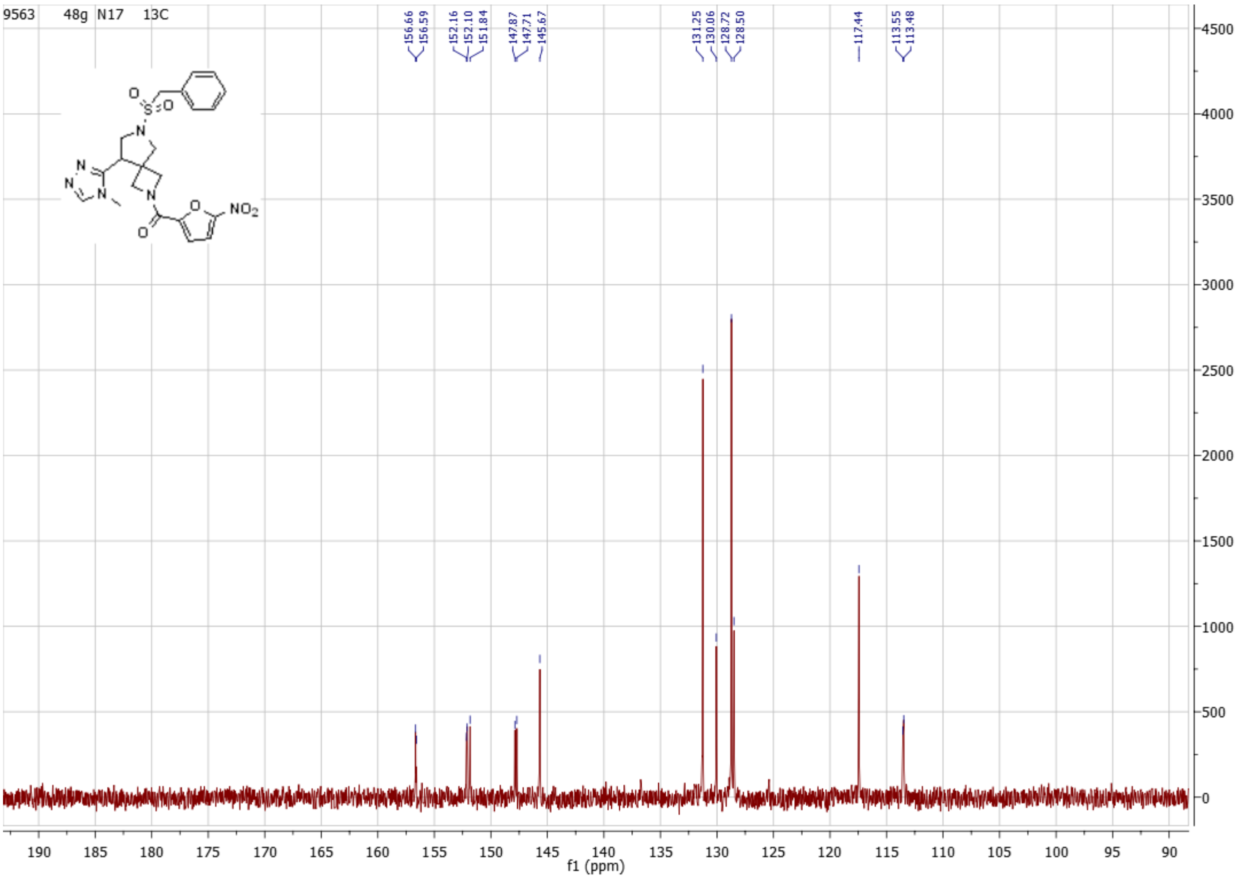

6-[(3,5-difluorophenyl)sulfonyl]-8-(4-methyl-4H-1,2,4-triazol-3-yl)-2-(5-nitro-2-furoyl)- 2,6-diazaspiro[3.4]octane ***2h***

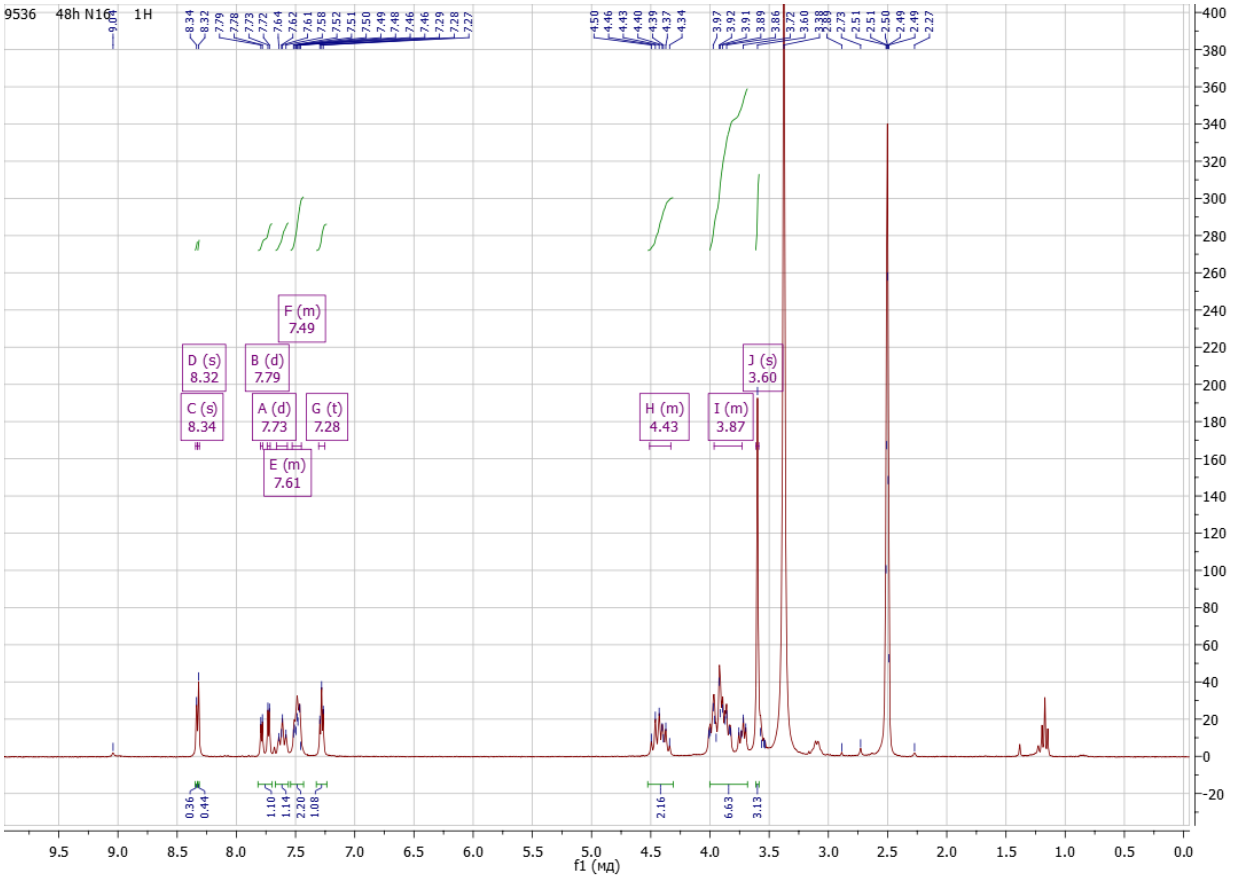

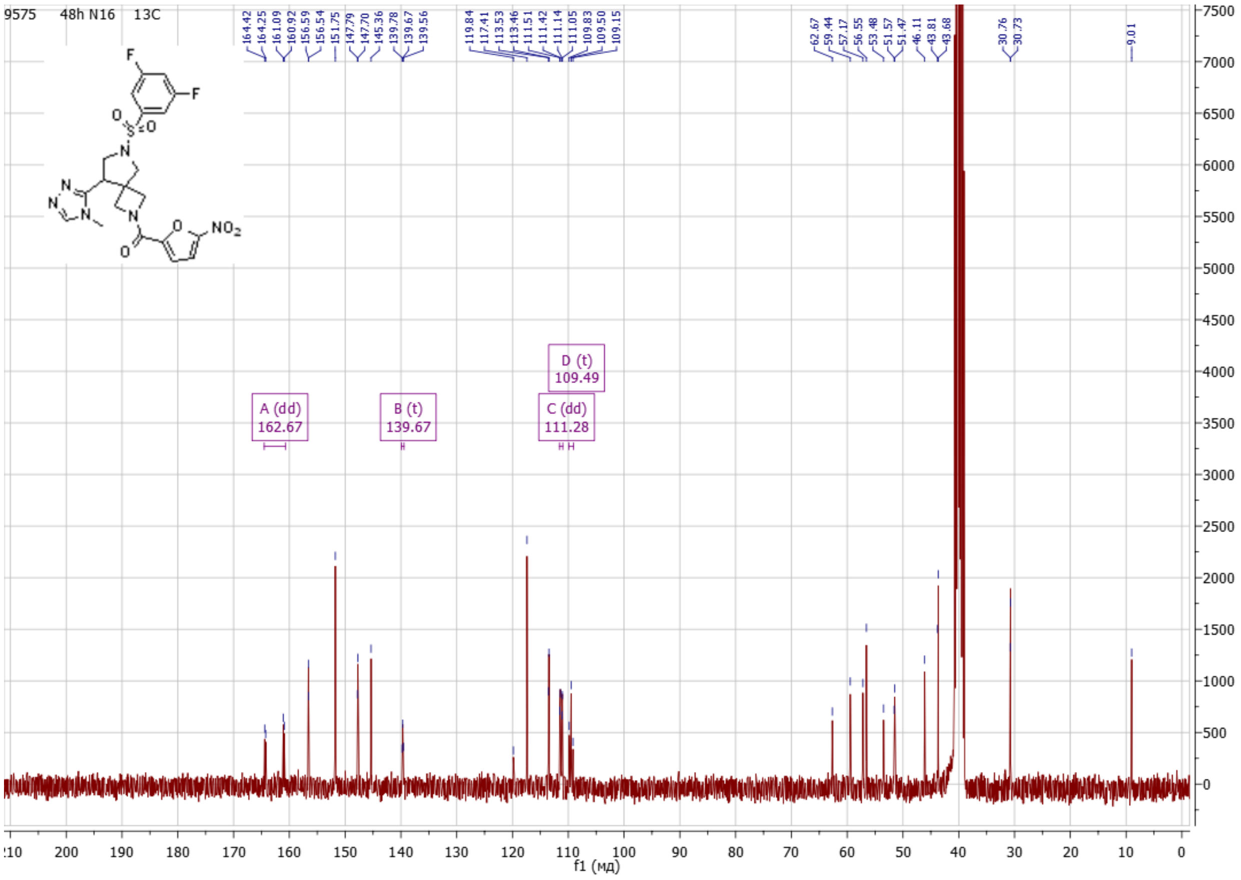

*6-[(3-fluoro-4-methylphenyl)sulfonyl]-8-(4-methyl-4H-1,2,4-triazol-3-yl)-2-(5-nitro-2- furoyl)-2,6-diazaspiro[3.4]octane **2i***

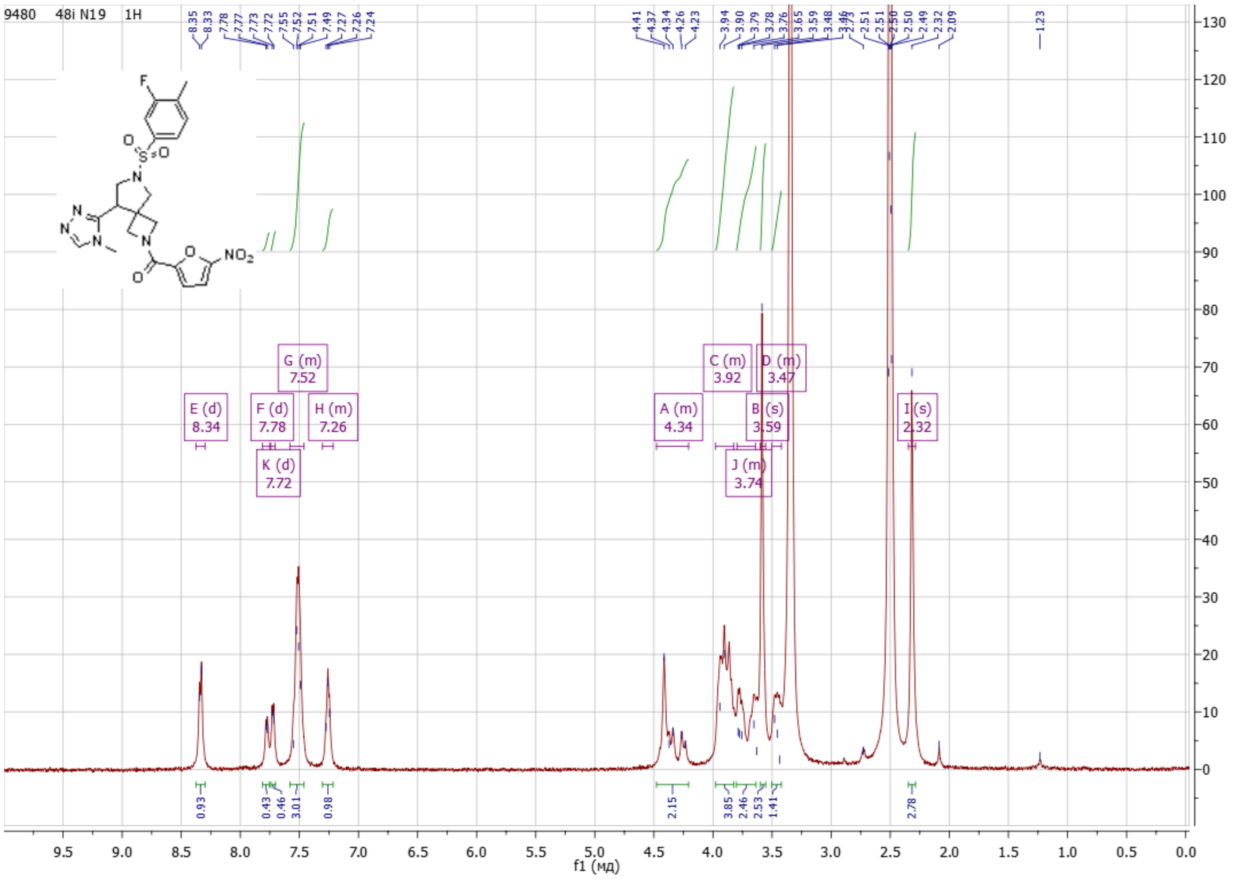

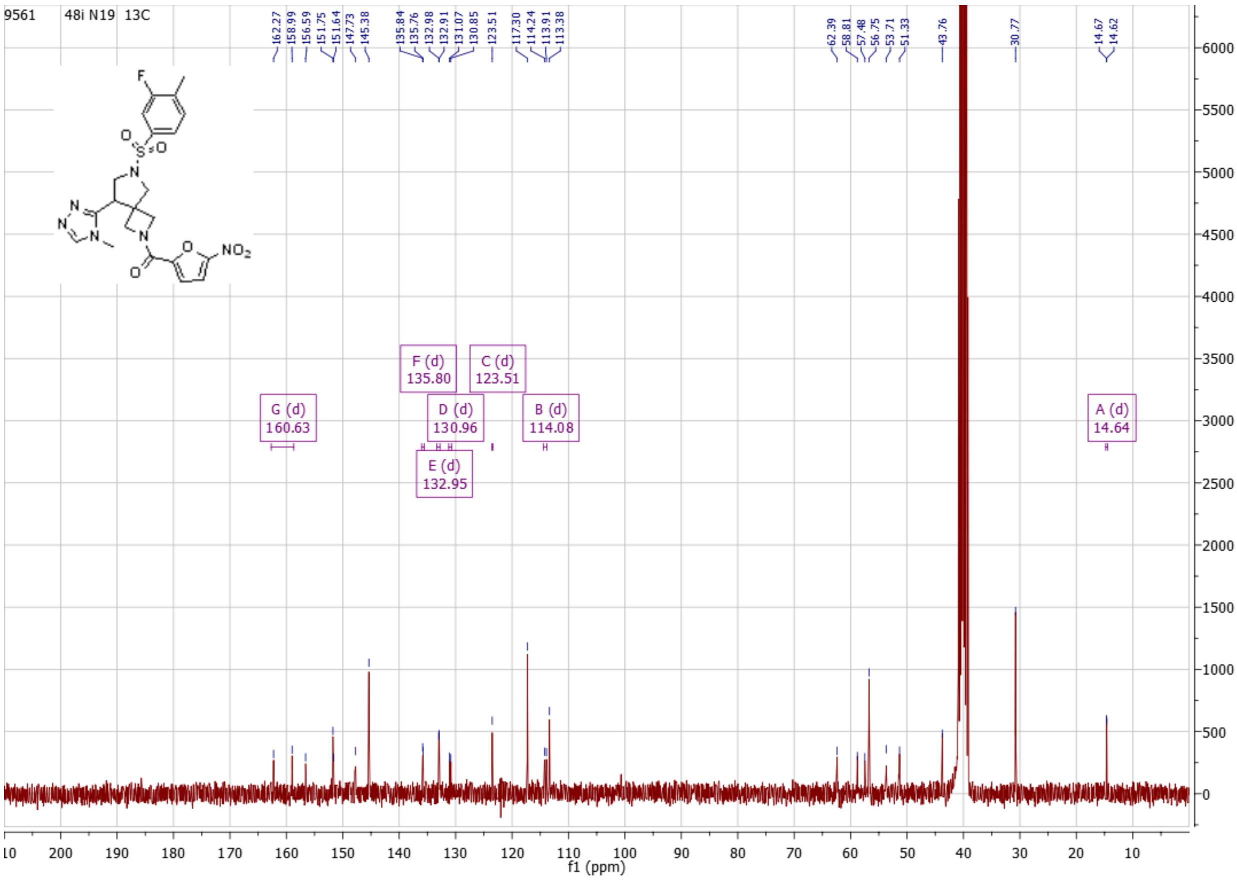

6-[(3-chlorophenyl)sulfonyl]-8-(4-methyl-4H-1,2,4-triazol-3-yl)-2-(5-nitro-2-furoyl)-2,6- diazaspiro[3.4]octane ***2j***

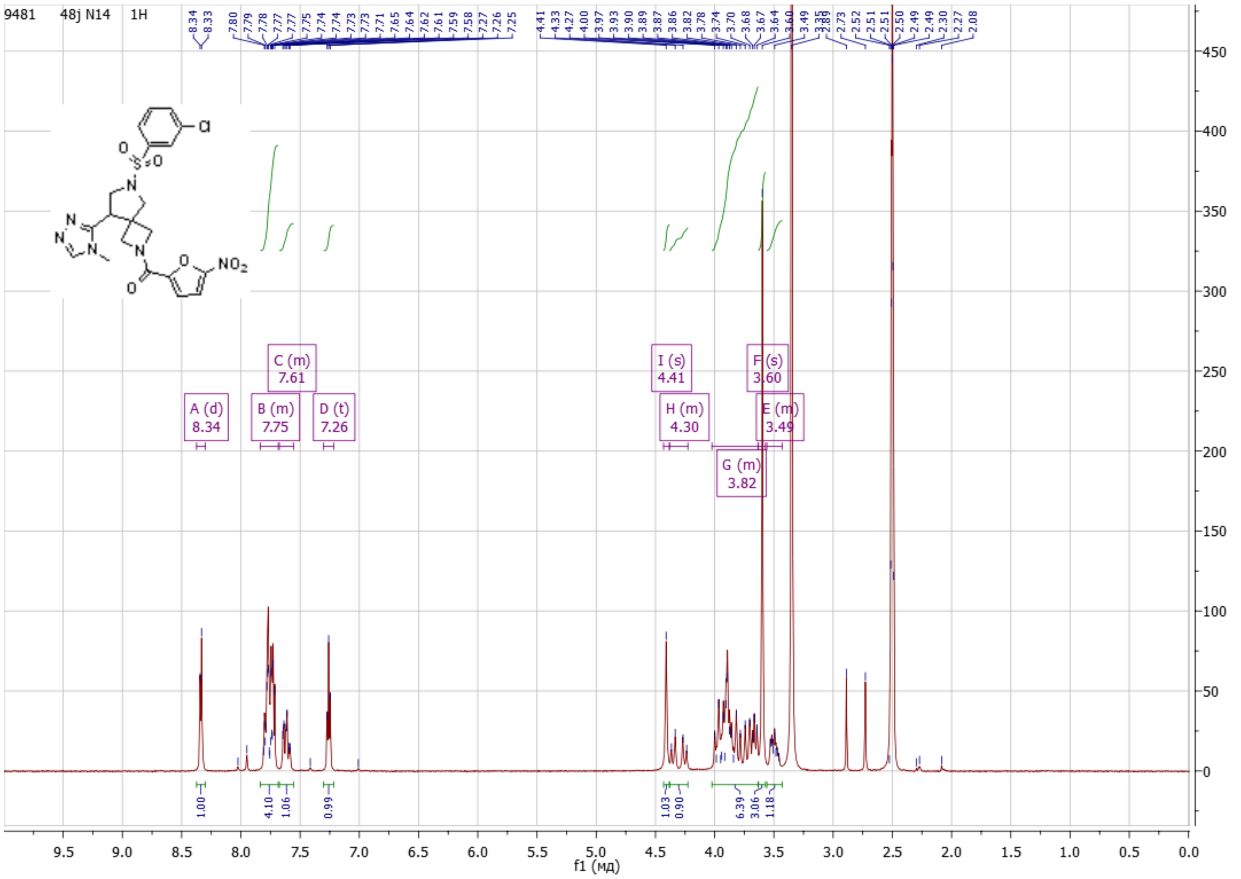

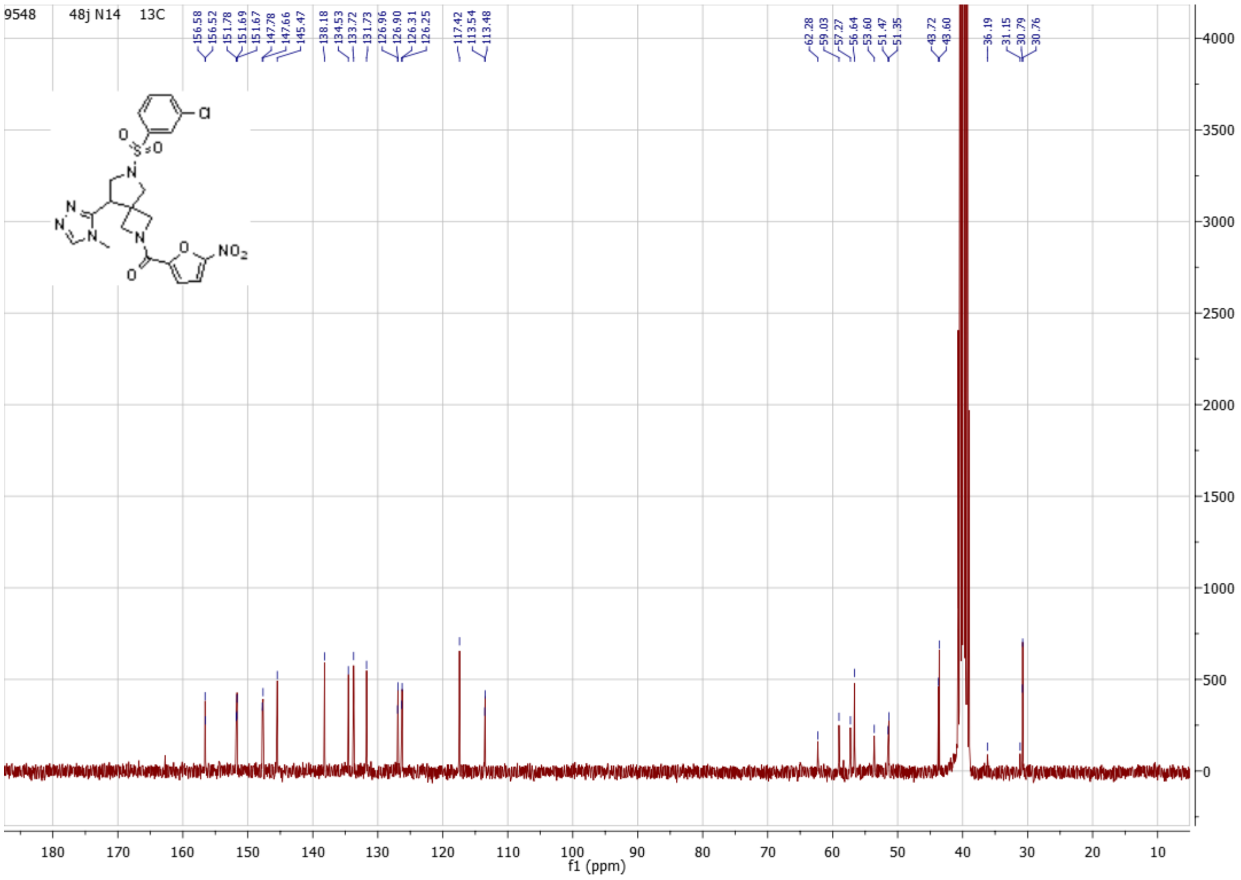

*2-(methylsulfonyl)-8-(4-methyl-4H-1,2,4-triazol-3-yl)-6-(5-nitro-2-furoyl)-2,6-diazaspiro [3.4]octane **3a***

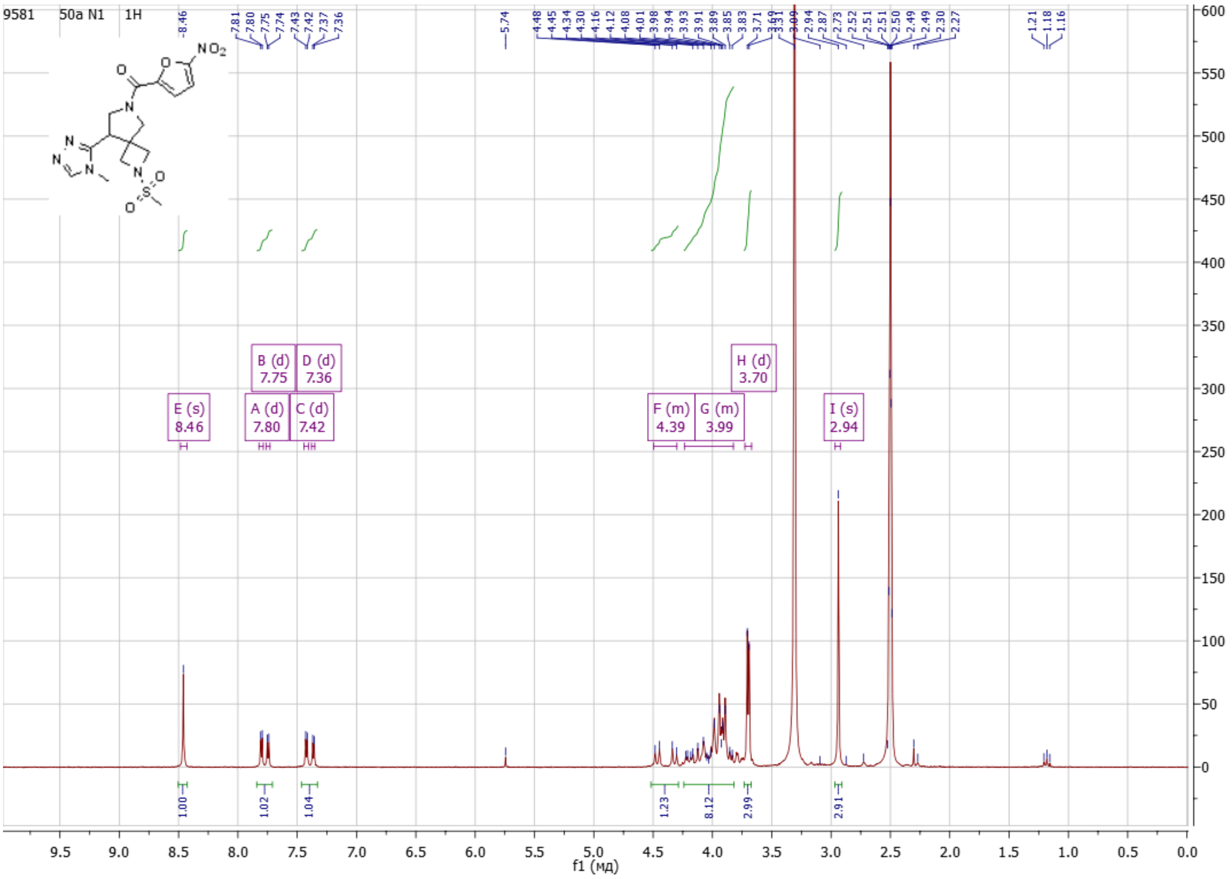

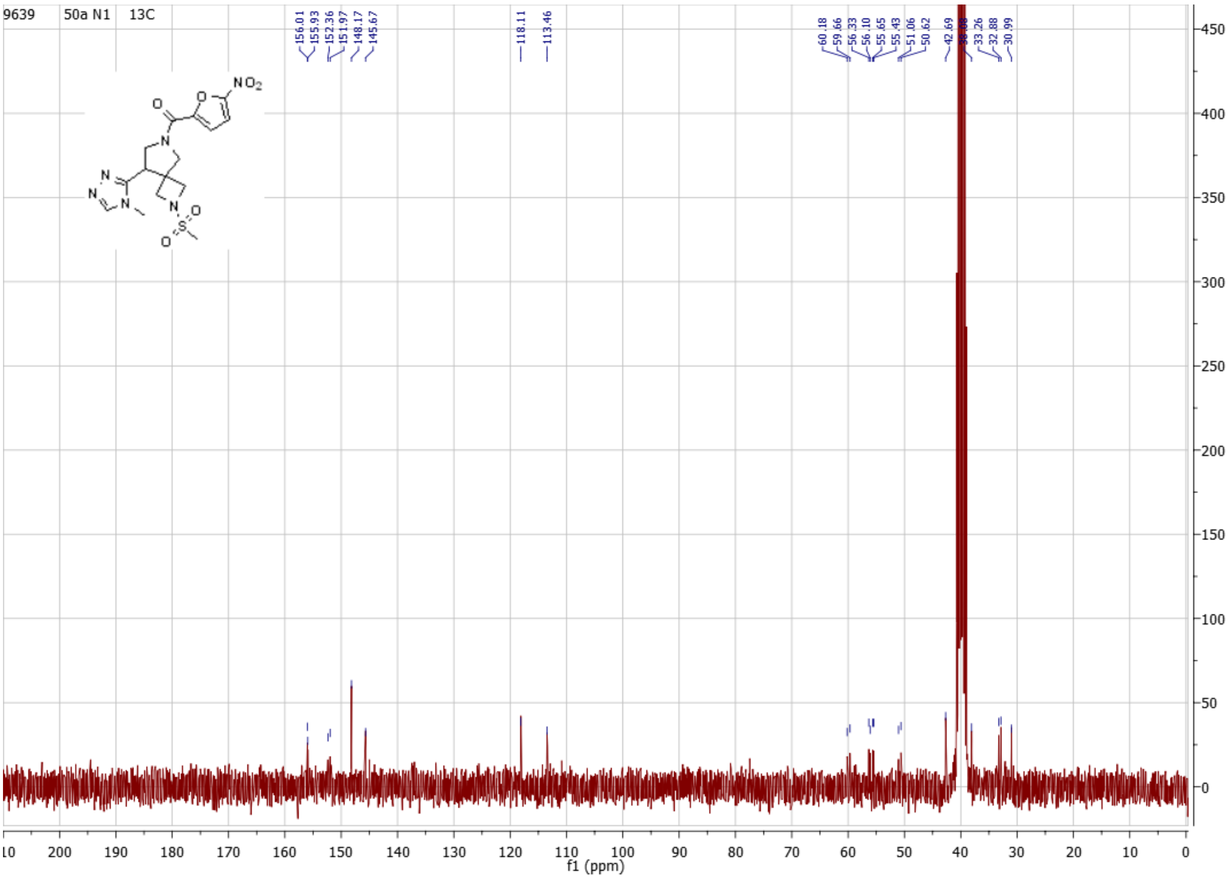

*2-(ethylsulfonyl)-8-(4-methyl-4H-1,2,4-triazol-3-yl)-6-(5-nitro-2-furoyl)-2,6-diazaspiro [3.4]octane **3b***

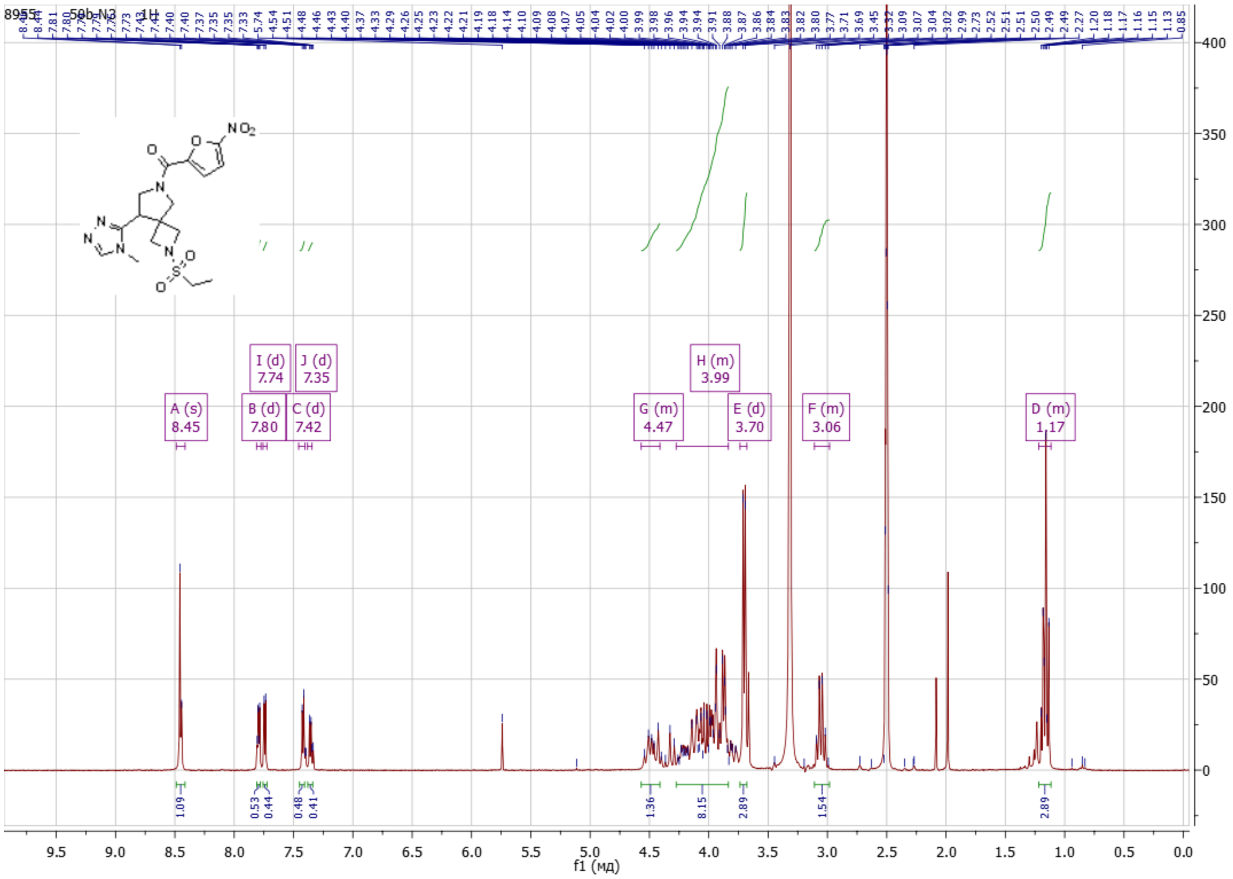

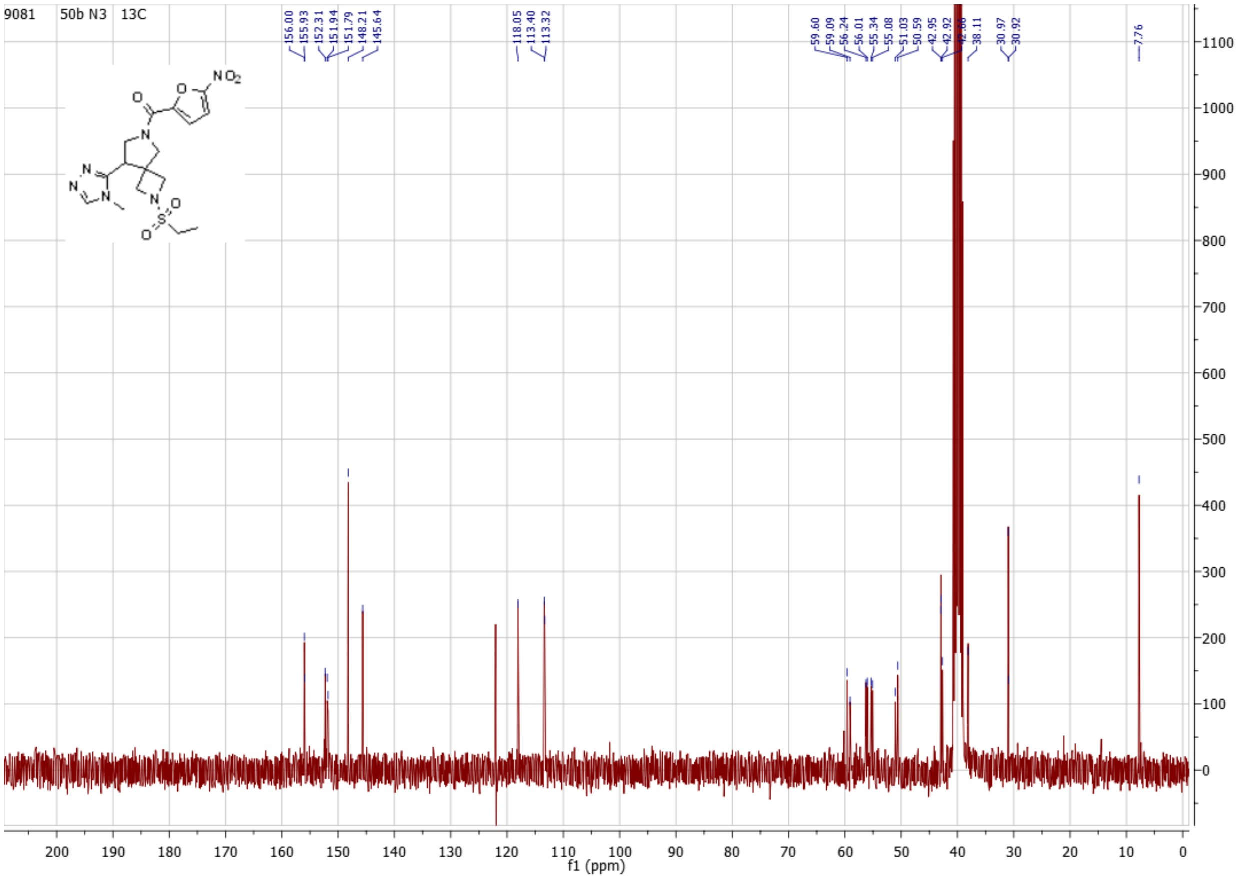

*2-(isopropylsulfonyl)-8-(4-methyl-4H-1,2,4-triazol-3-yl)-6-(5-nitro-2-furoyl)-2,6-diazaspiro[3.4]octane **3c***

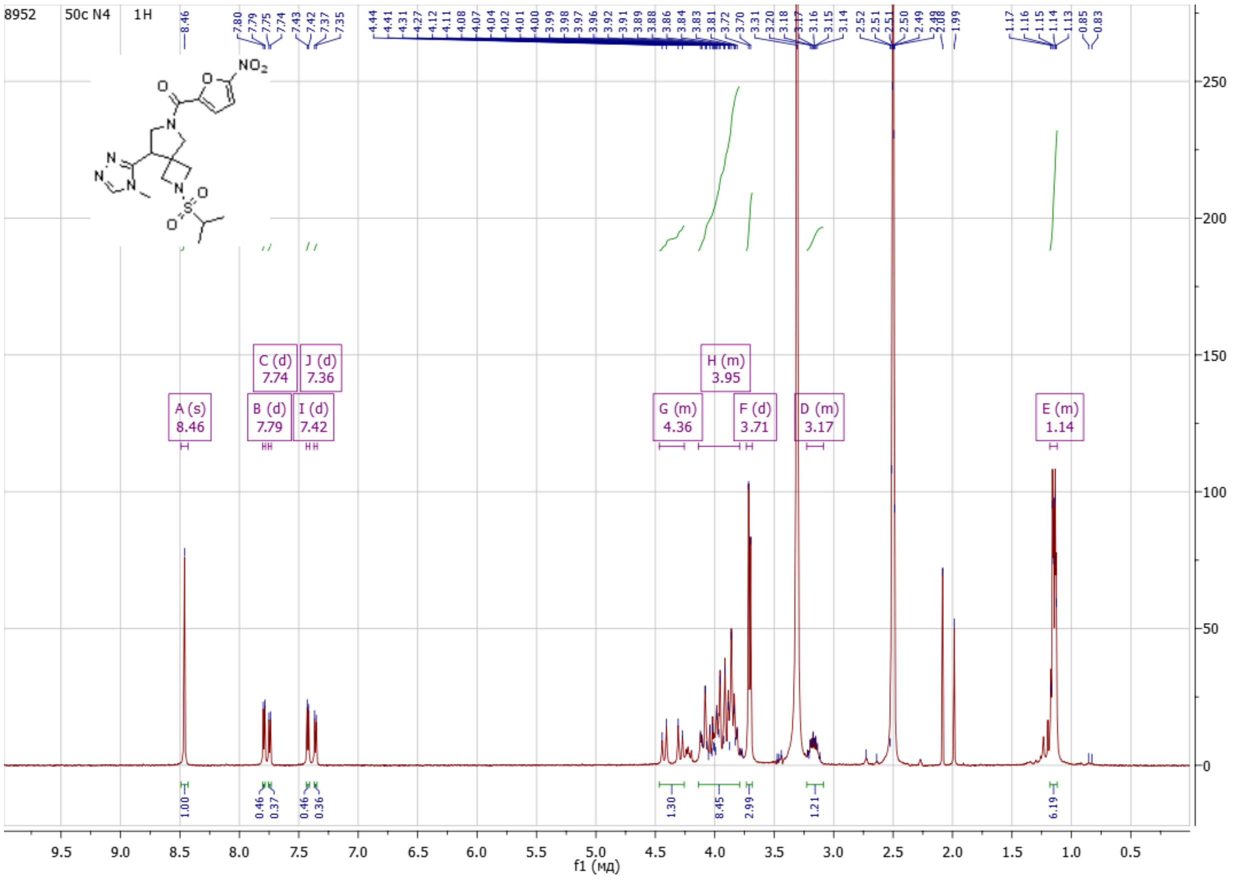

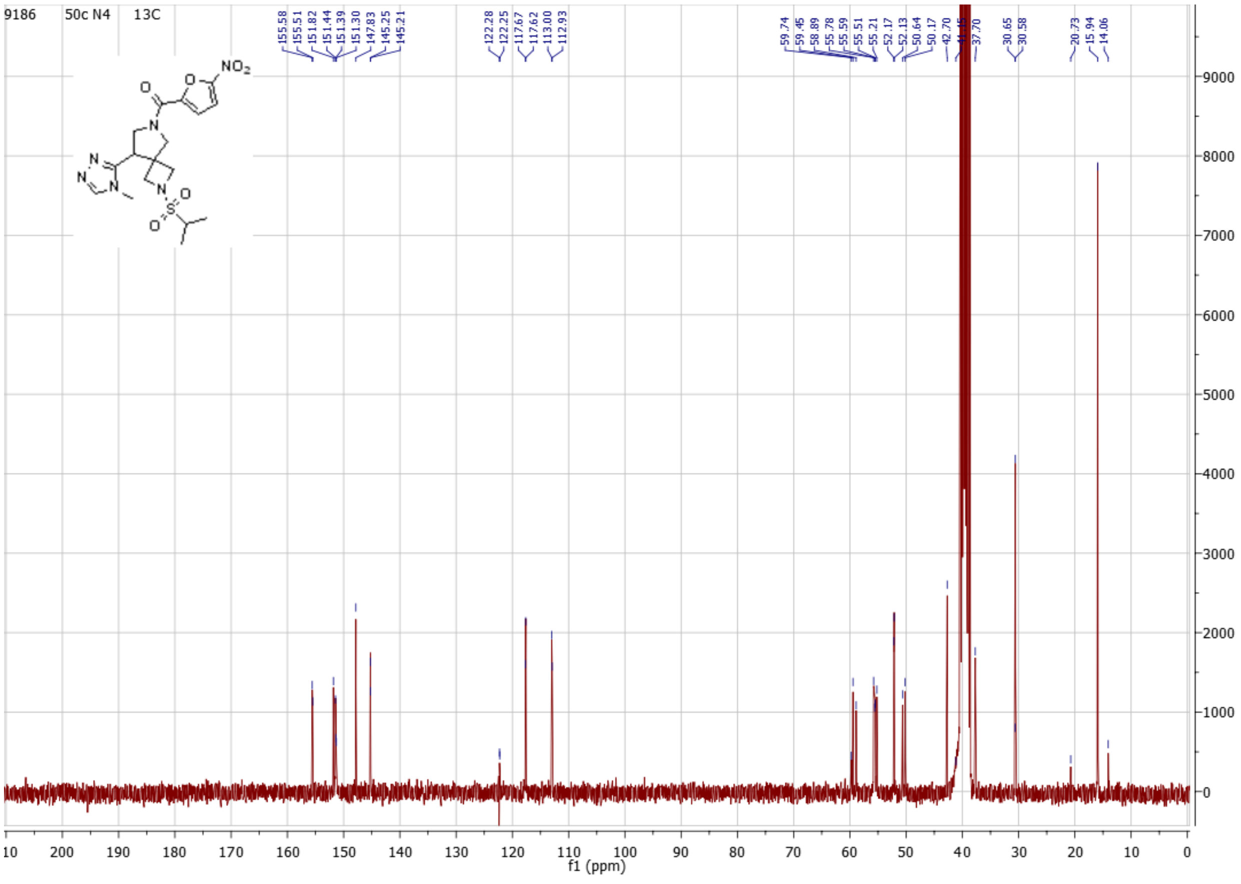

2-(cyclopropylsulfonyl)-8-(4-methyl-4H-1,2,4-triazol-3-yl)-6-(5-nitro-2-furoyl)-2,6- diazaspiro[3.4]octane ***3d***

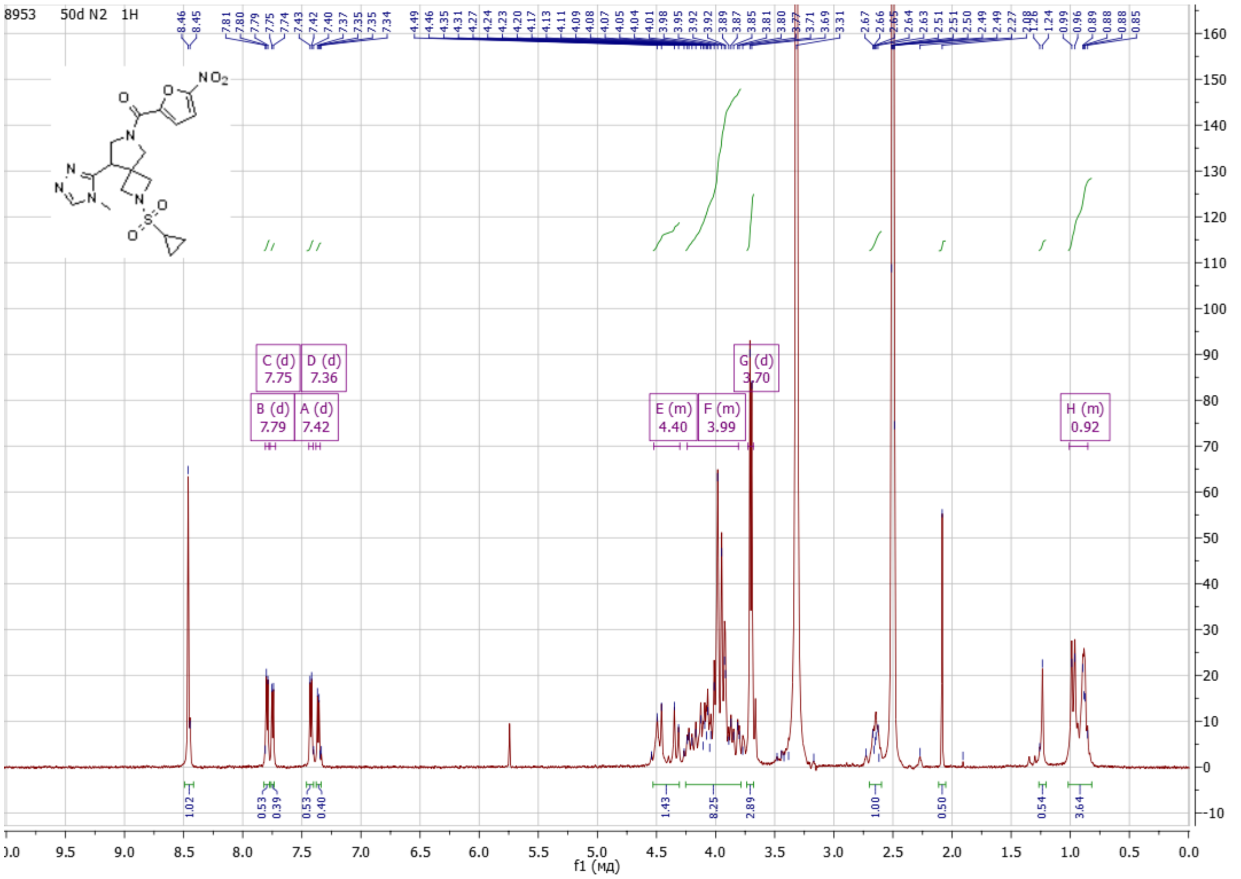

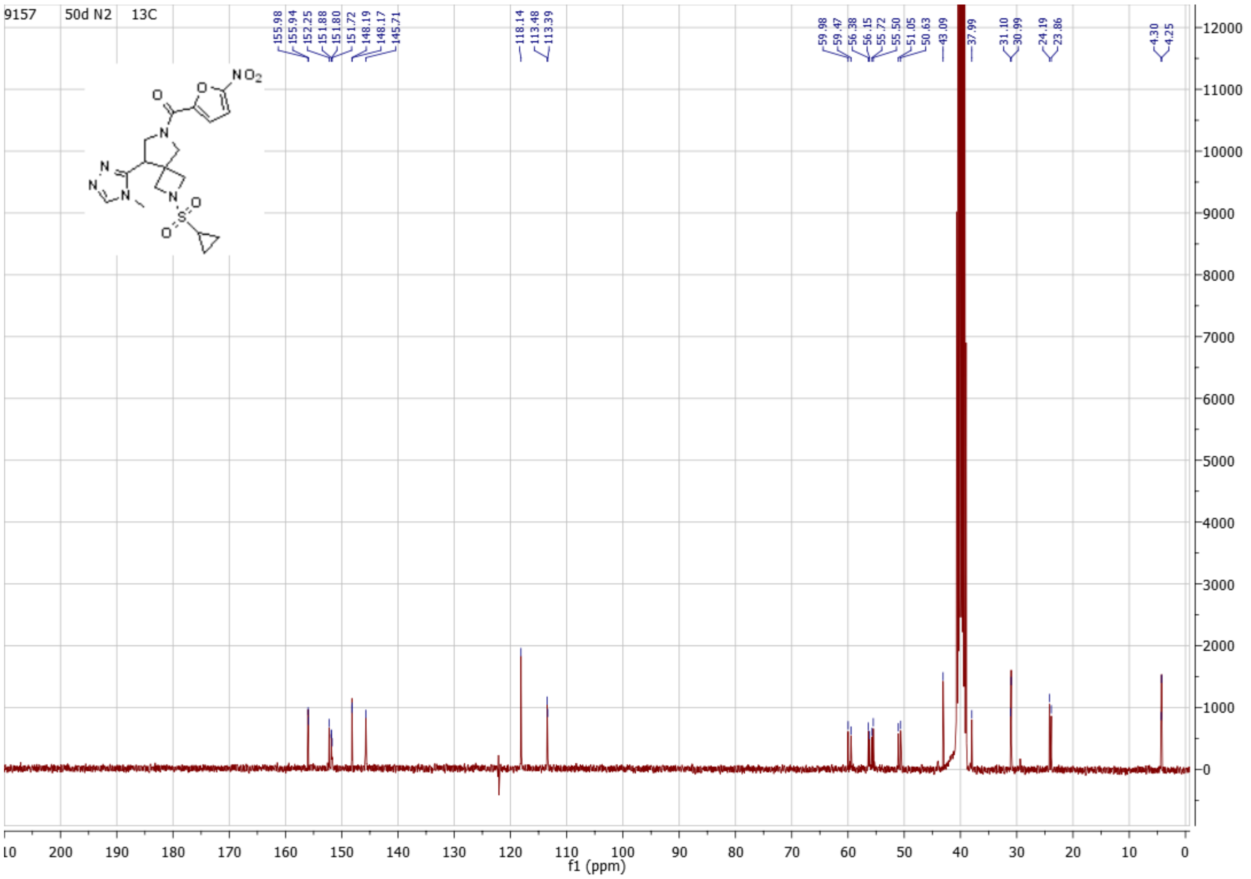

2-(butylsulfonyl)-8-(4-methyl-4H-1,2,4-triazol-3-yl)-6-(5-nitro-2-furoyl)-2,6-diazaspiro [3.4]octane ***3e***

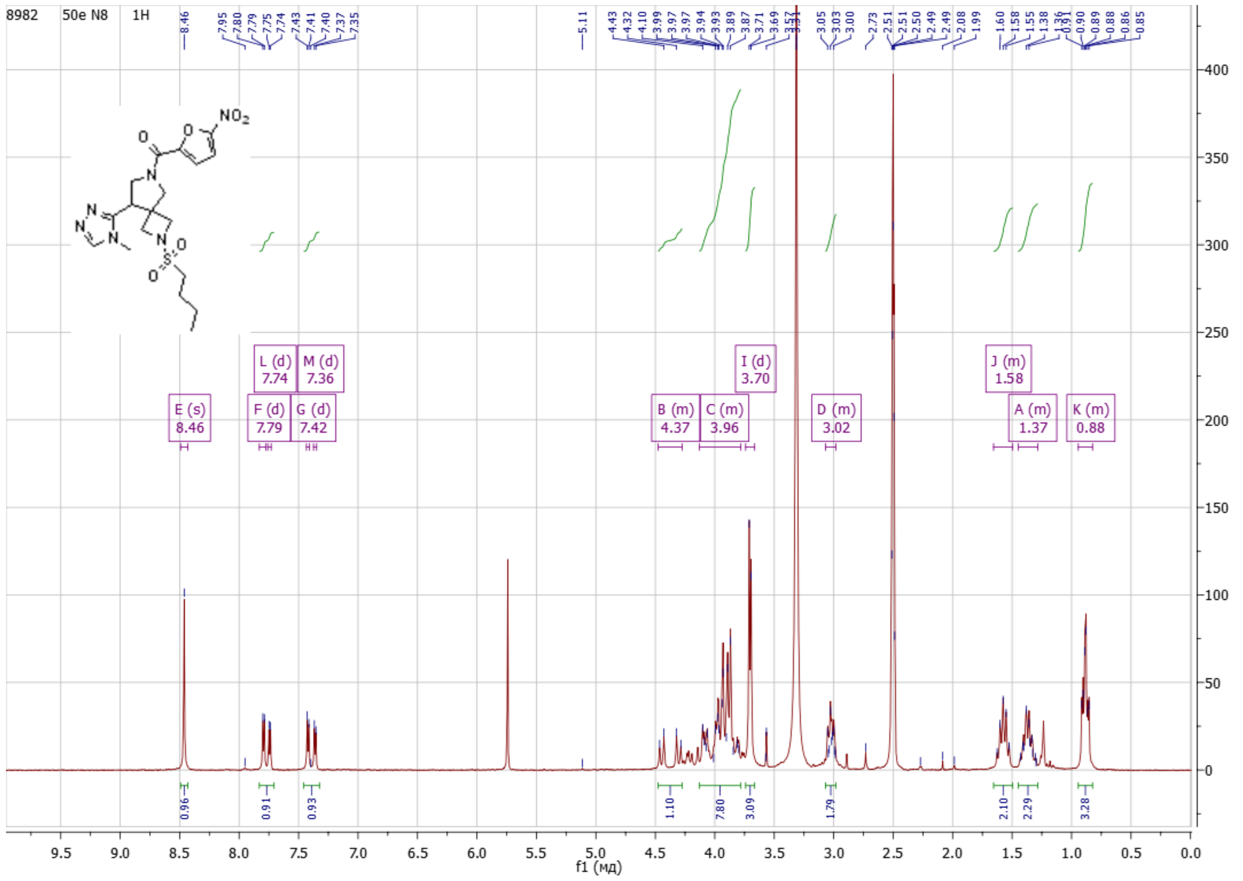

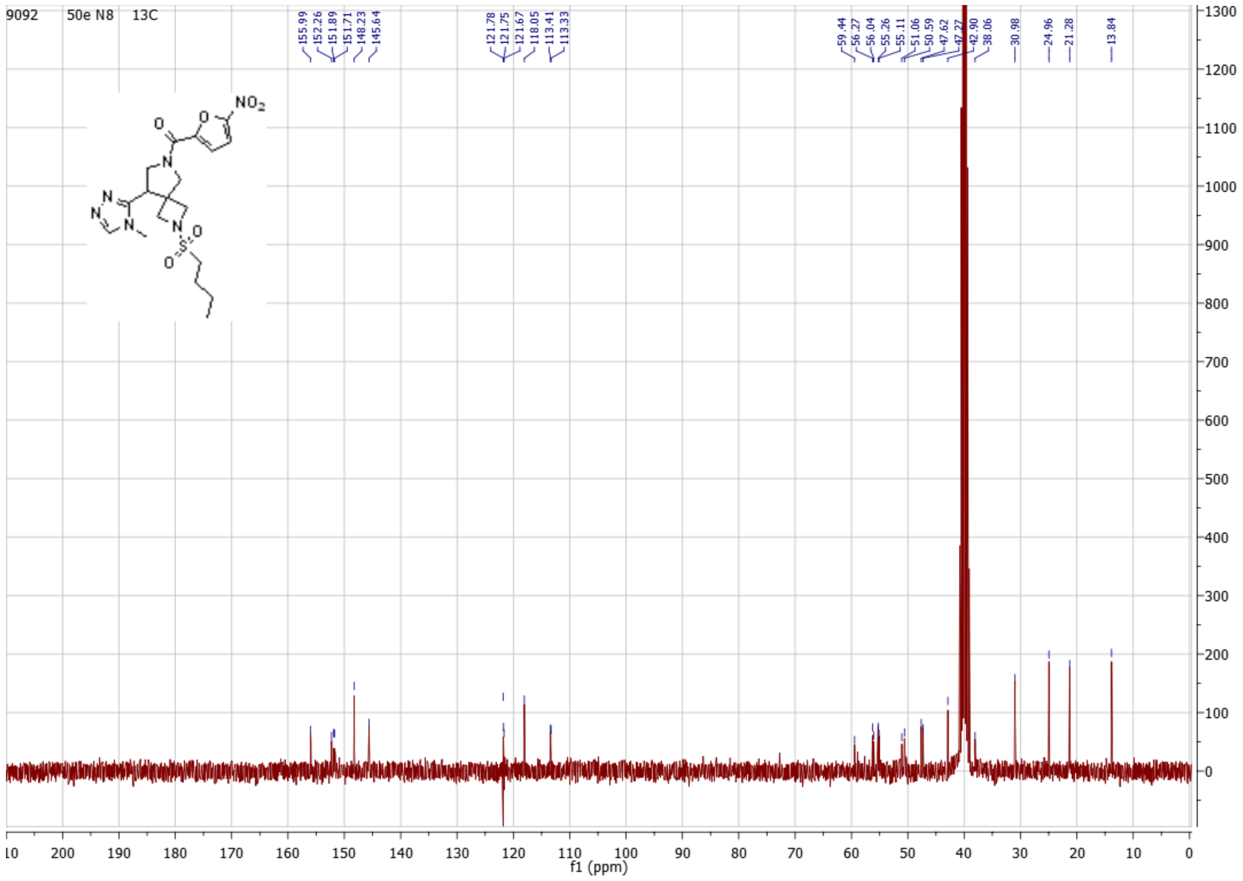

*6-(5-nitro-2-furoyl)-8-(4-propyl-4H-1,2,4-triazol-3-yl)-6-azaspiro[3.4]octane **4a***

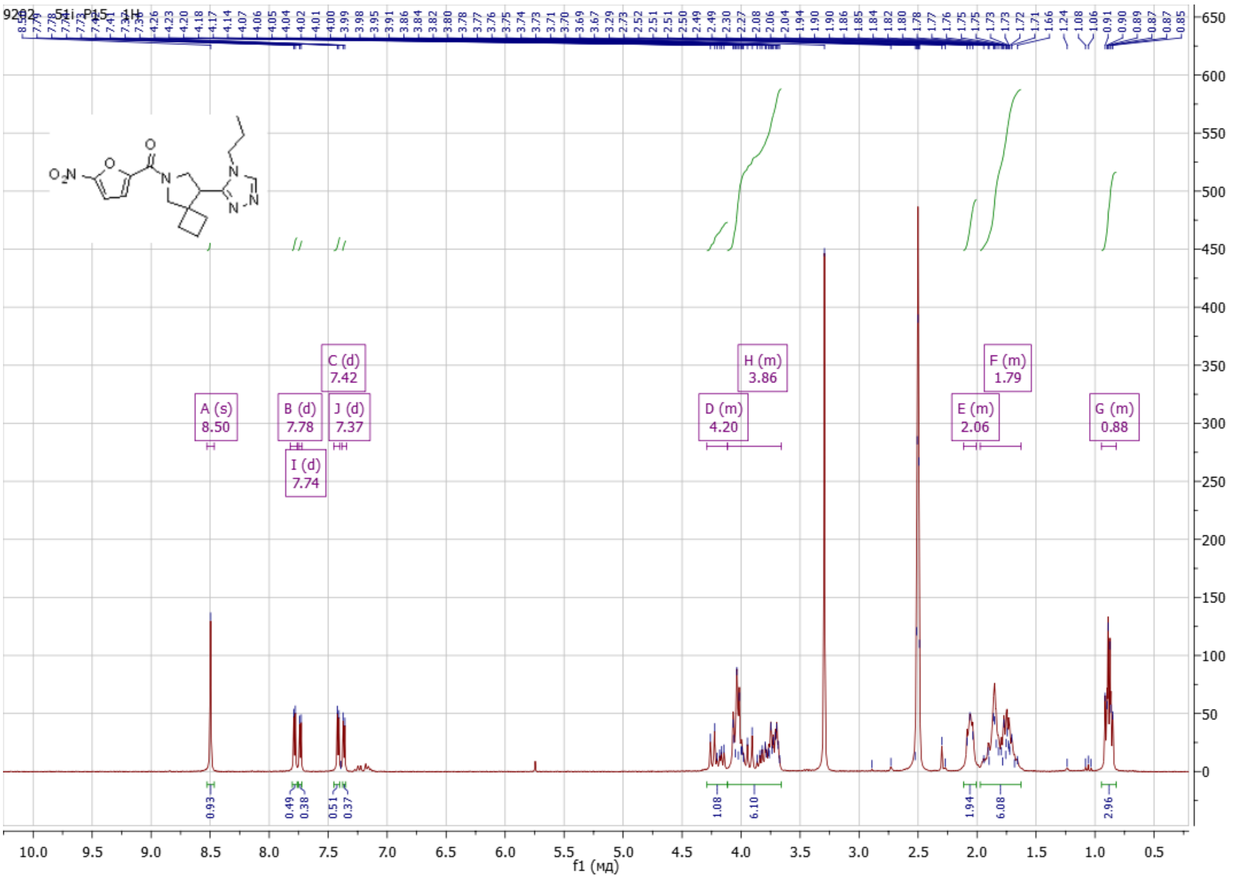

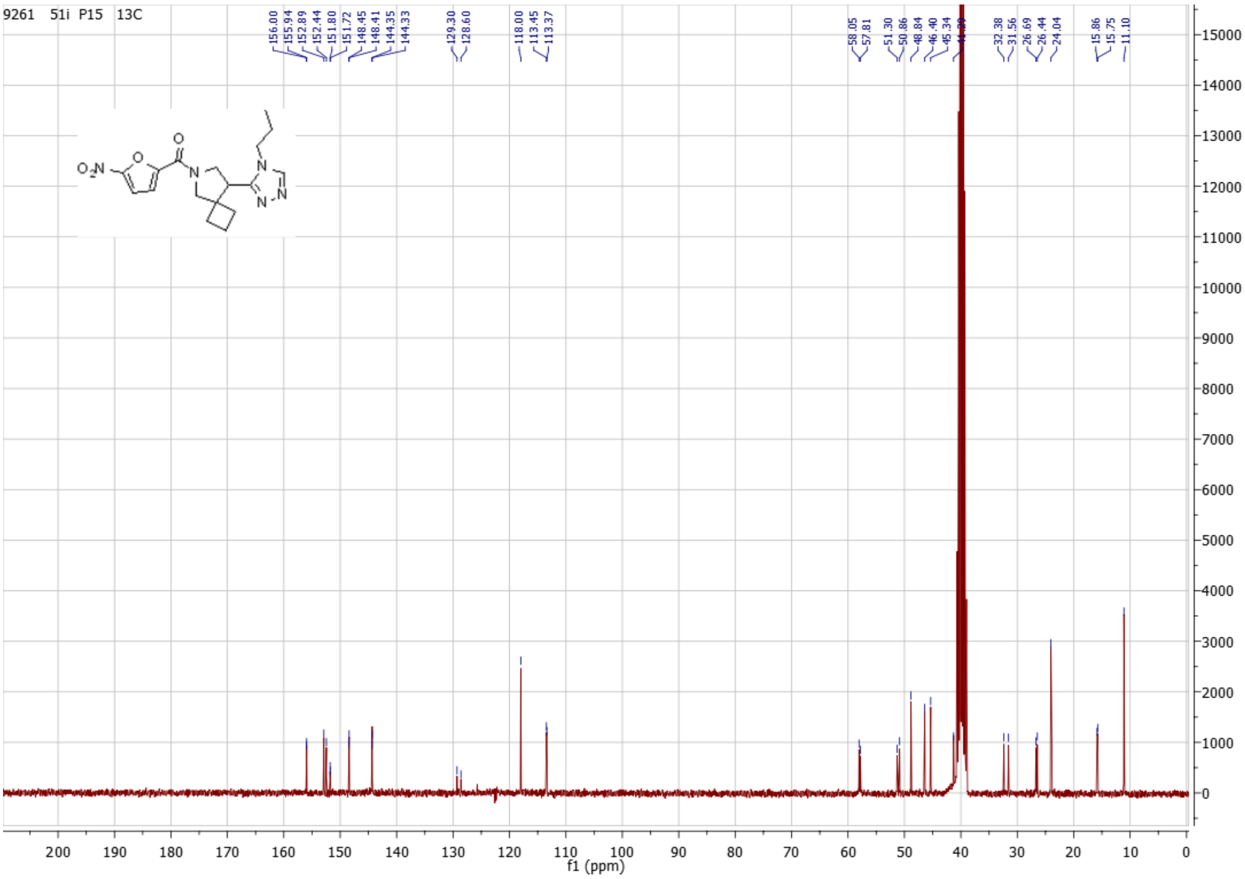

4-(4-methyl-4H-1,2,4-triazol-3-yl)-2-(5-nitro-2-furoyl)-2-azaspiro[4.4]nonane ***4b***

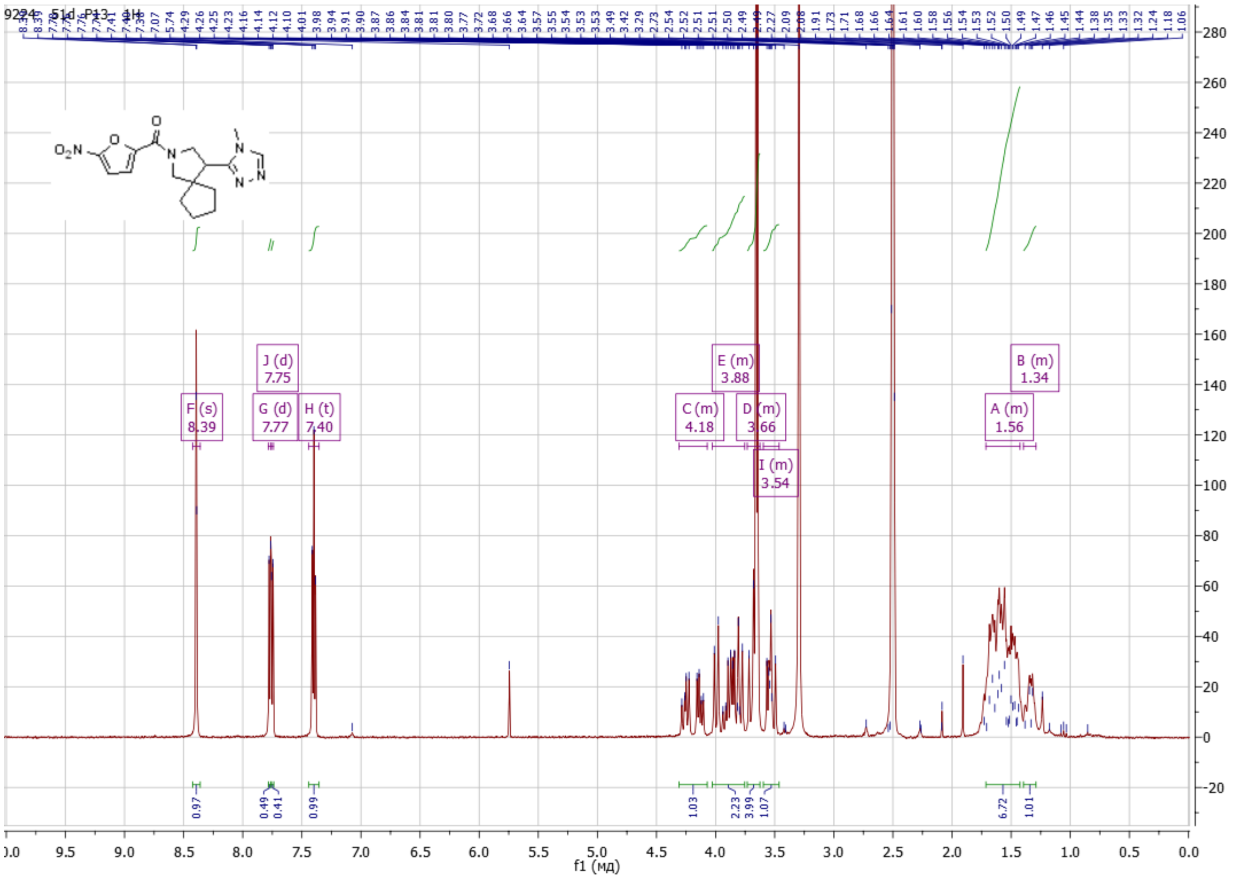

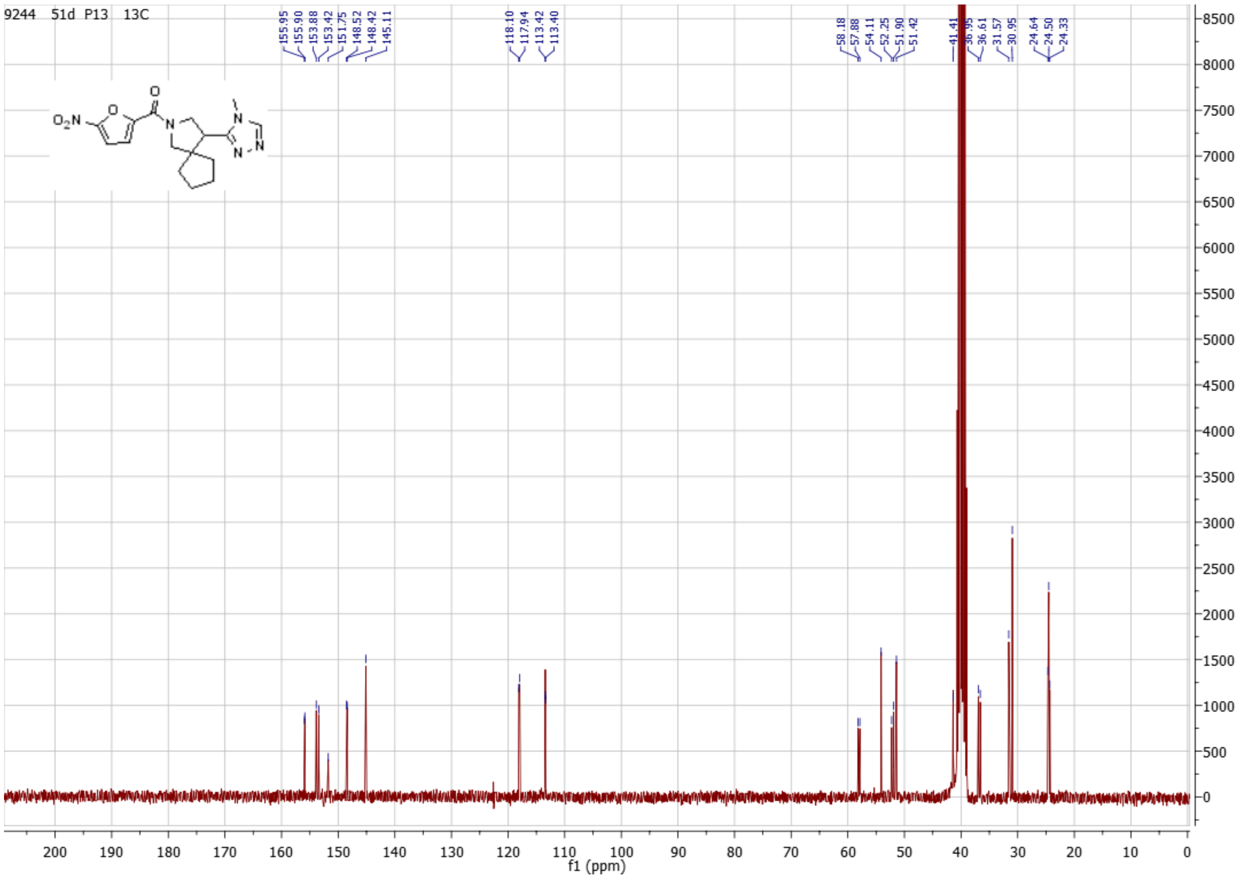

4-(4-methyl-4H-1,2,4-triazol-3-yl)-2-(5-nitro-2-furoyl)-2-azaspiro[4.5]decane ***4c***

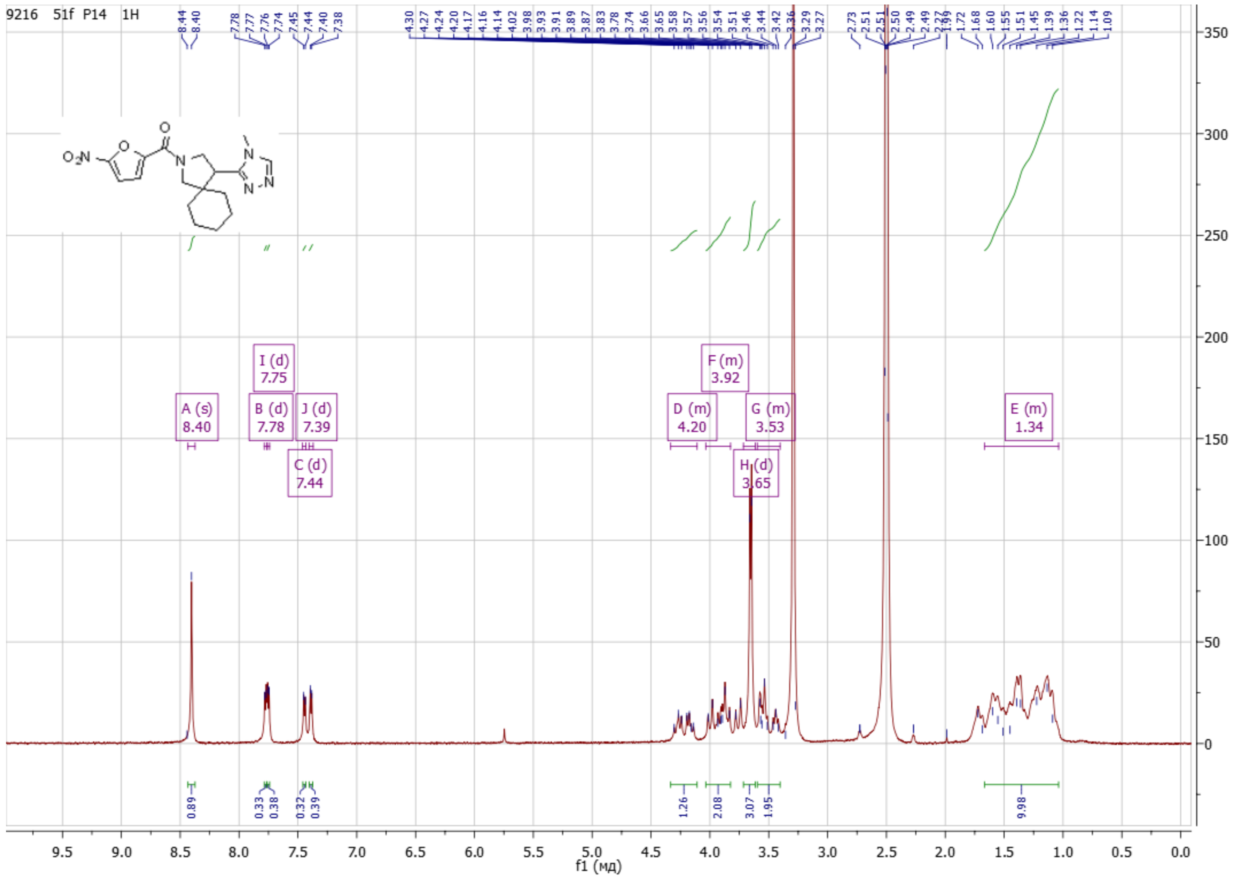

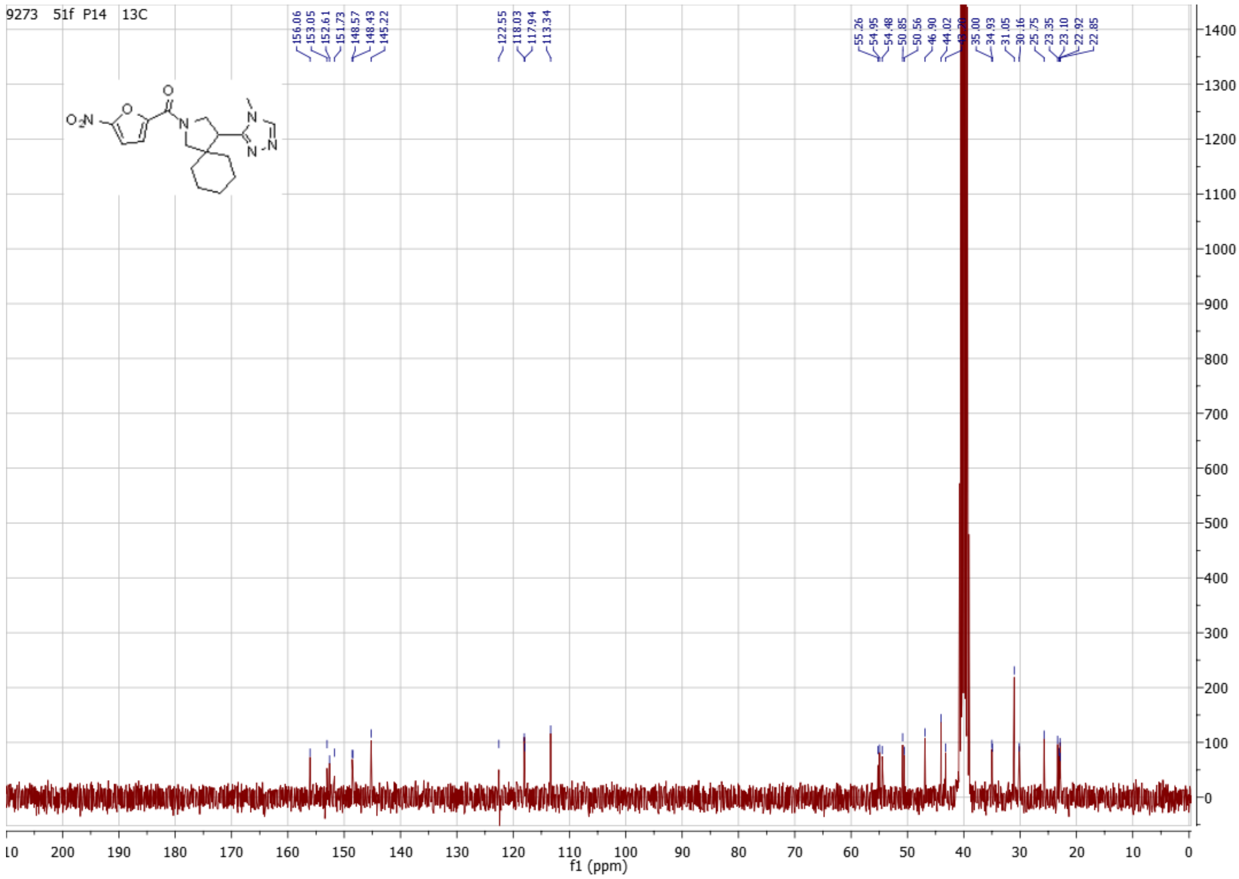

4-(4-methyl-4H-1,2,4-triazol-3-yl)-2-(5-nitro-2-furoyl)-8-oxa-2-azaspiro[4.5]decane ***4d***

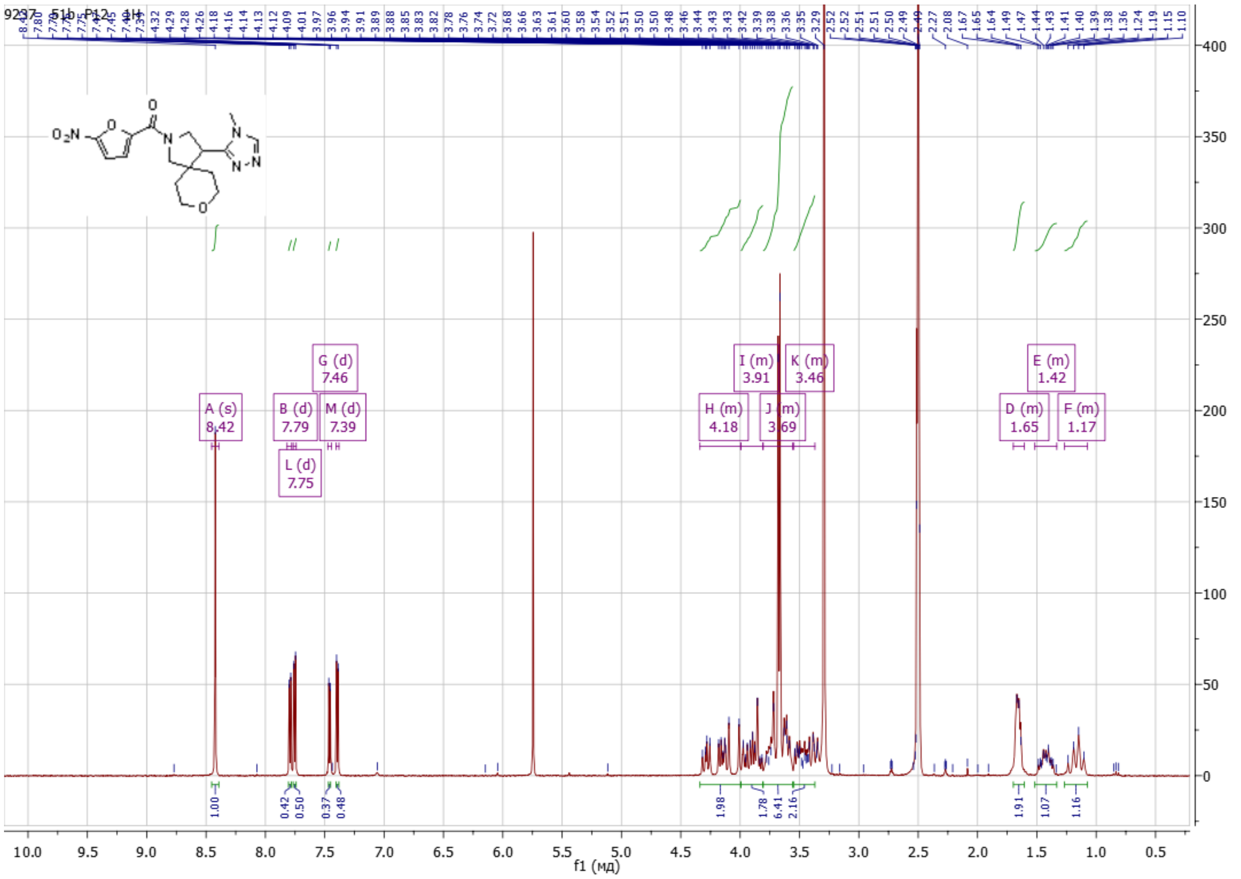

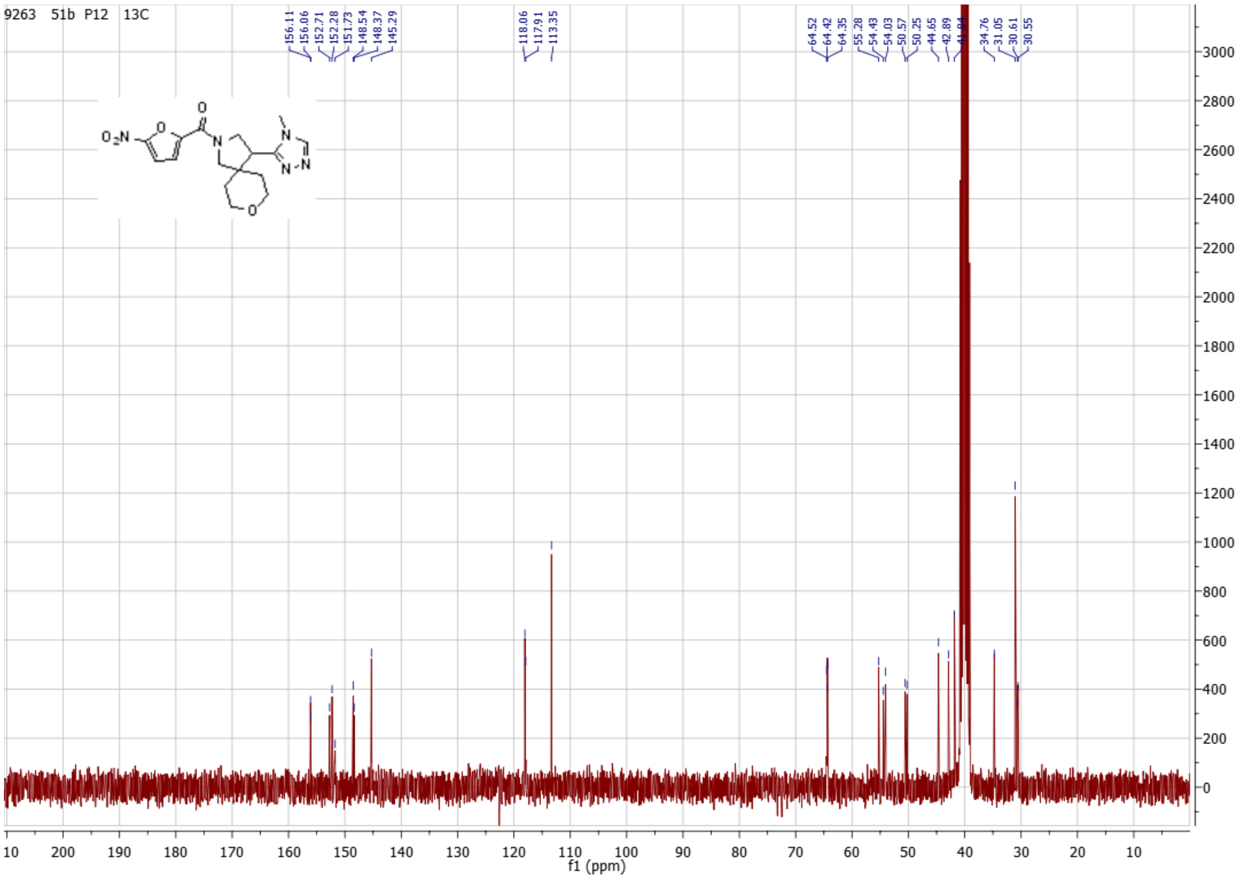

4-(cyclopropylmethyl)-3-{[1-(5-nitro-2-furoyl)azetidin-3-yl]methyl}-4H-1,2,4-triazole ***5a***

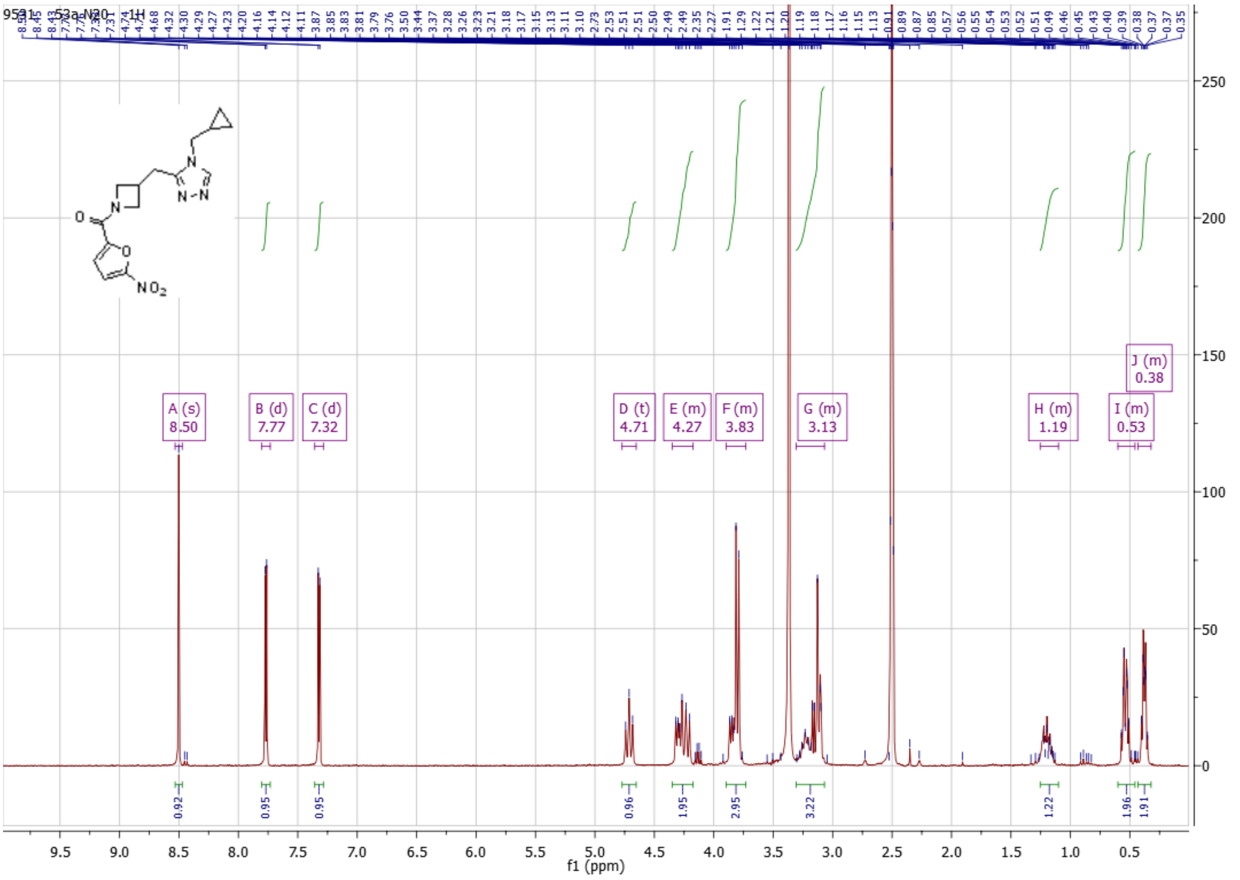

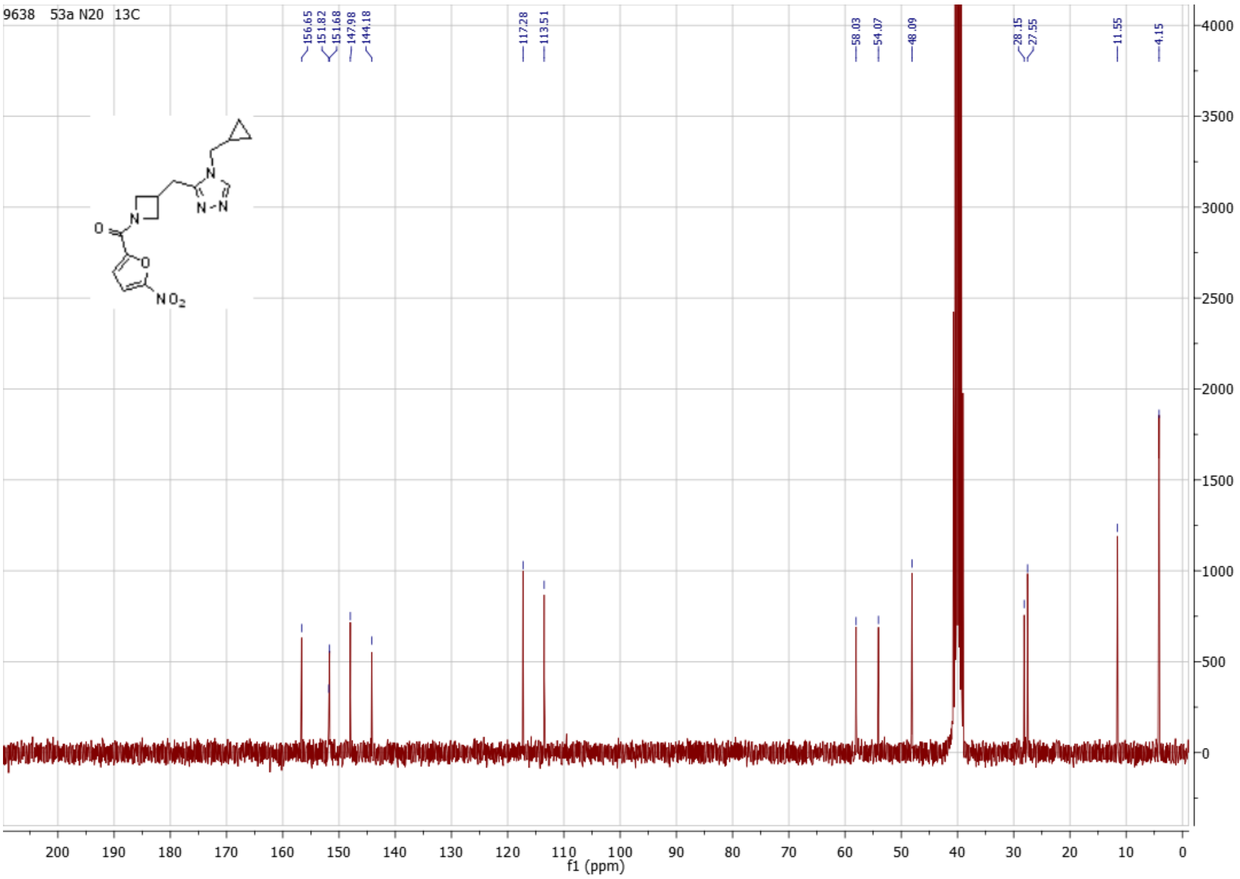

*4-isopropyl-3-{[1-(5-nitro-2-furoyl)azetidin-3-yl]methyl}-4H-1,2,4-triazole **5b***

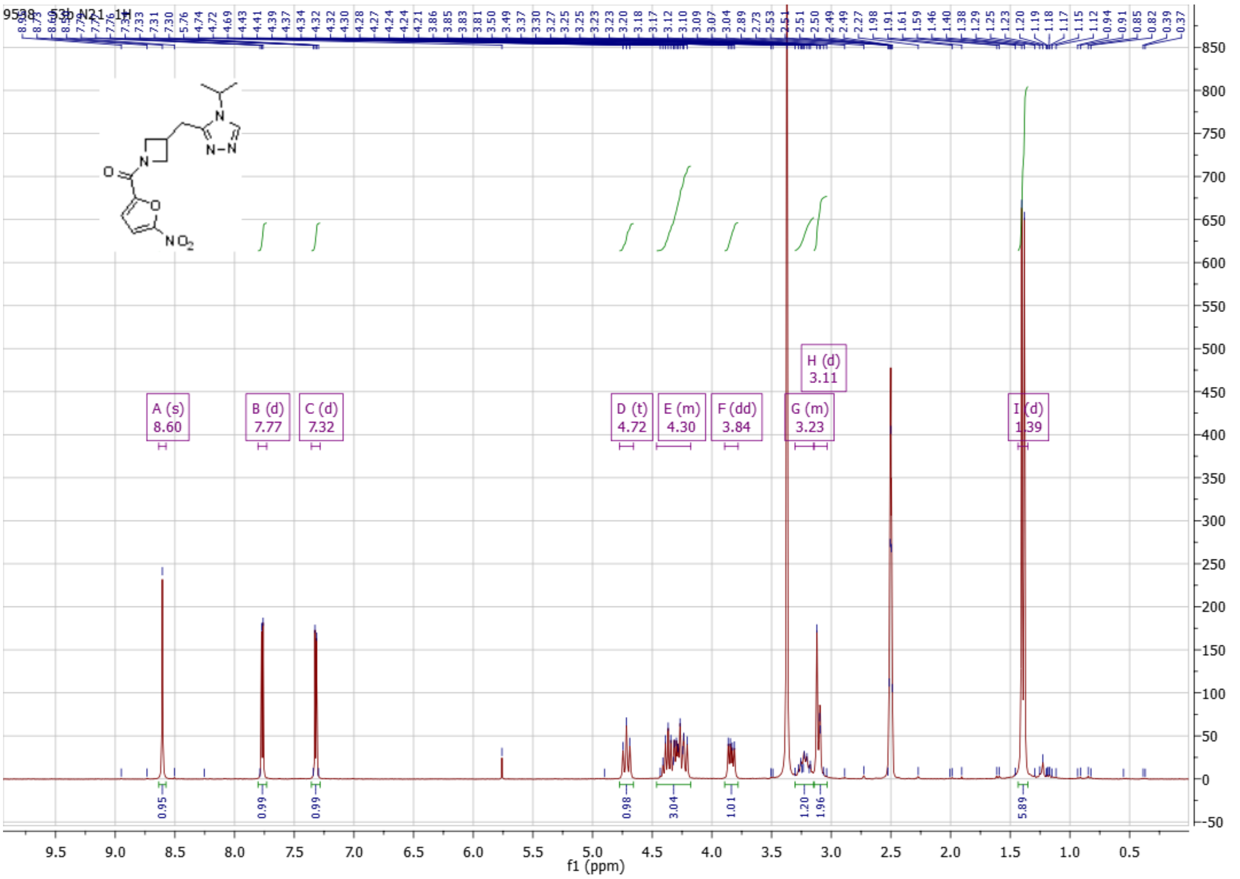

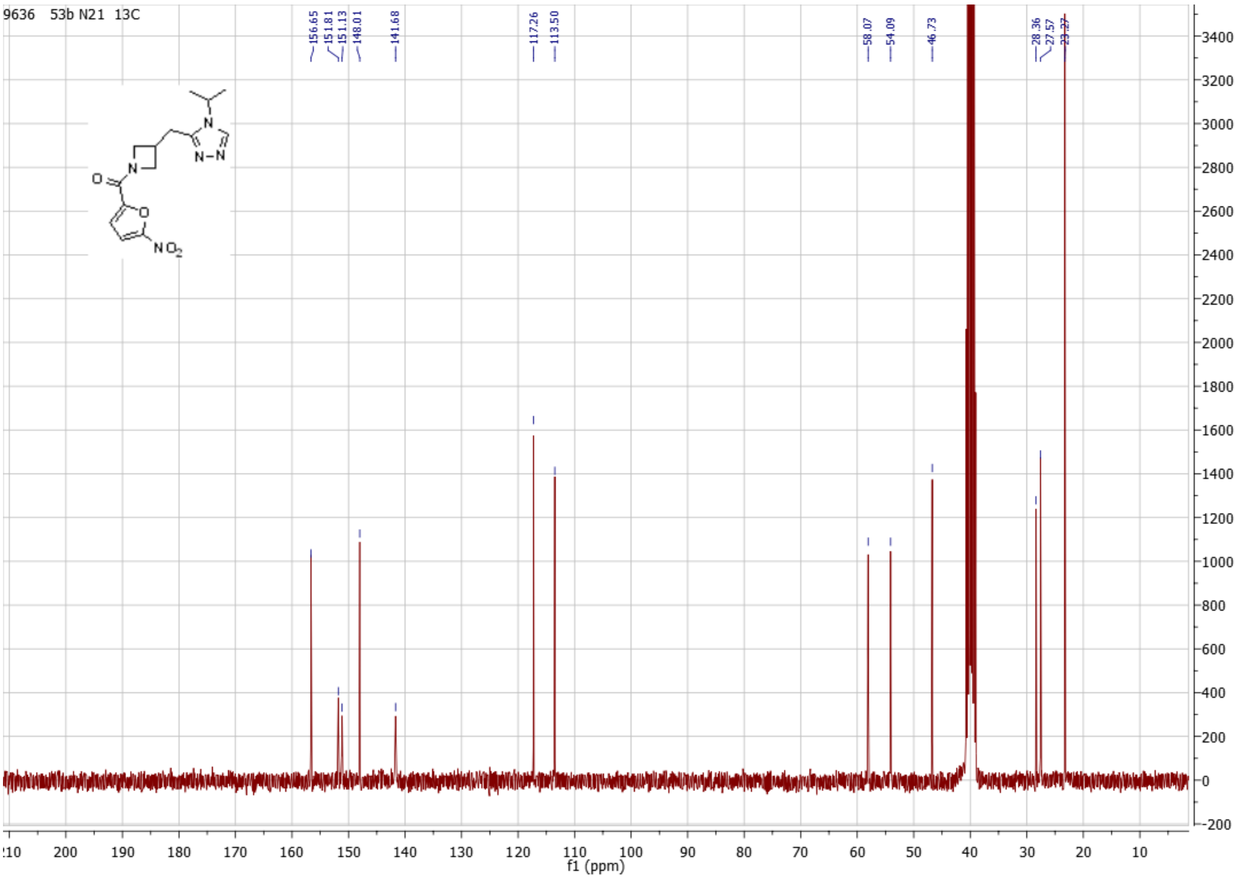

4-cyclopentyl-3-{[1-(5-nitro-2-furoyl)azetidin-3-yl]methyl}-4H-1,2,4-triazole ***5c***

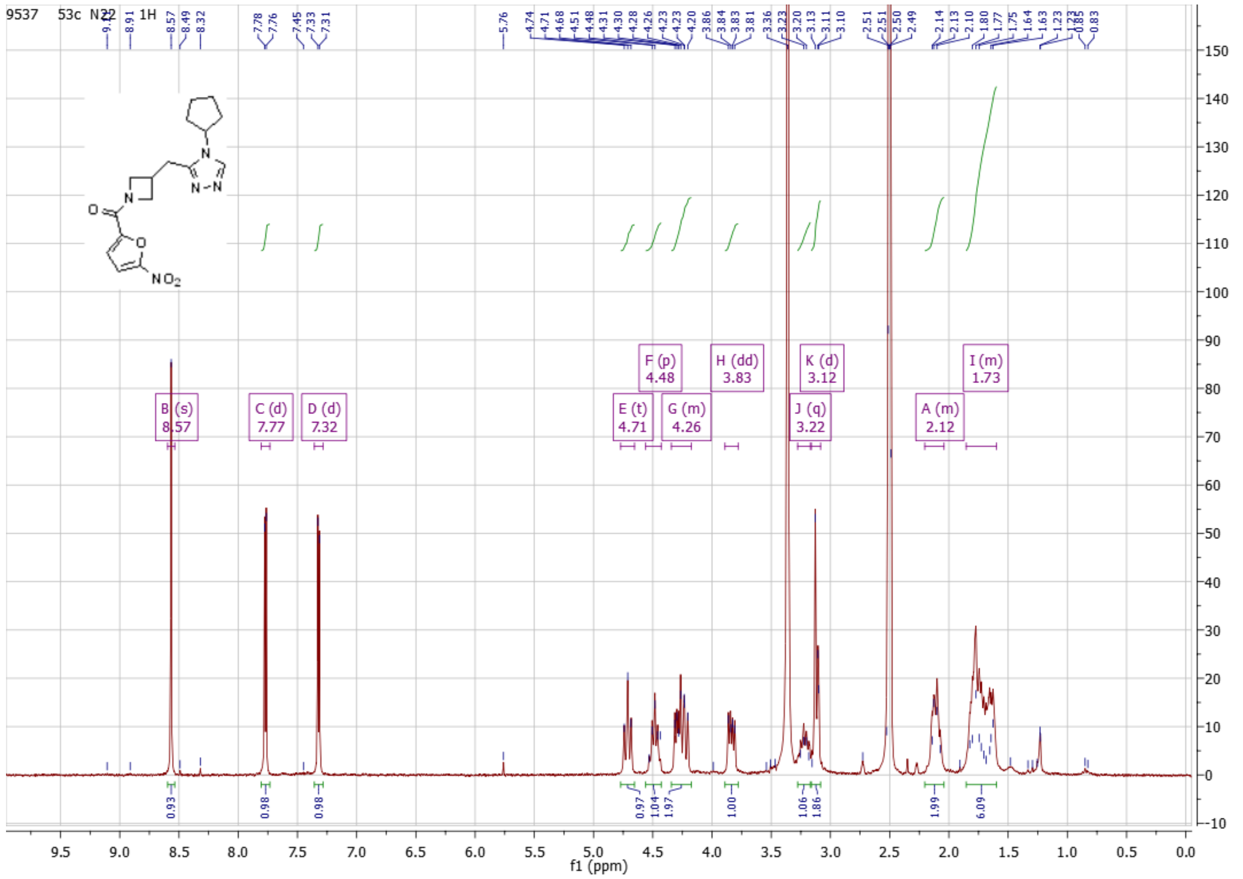

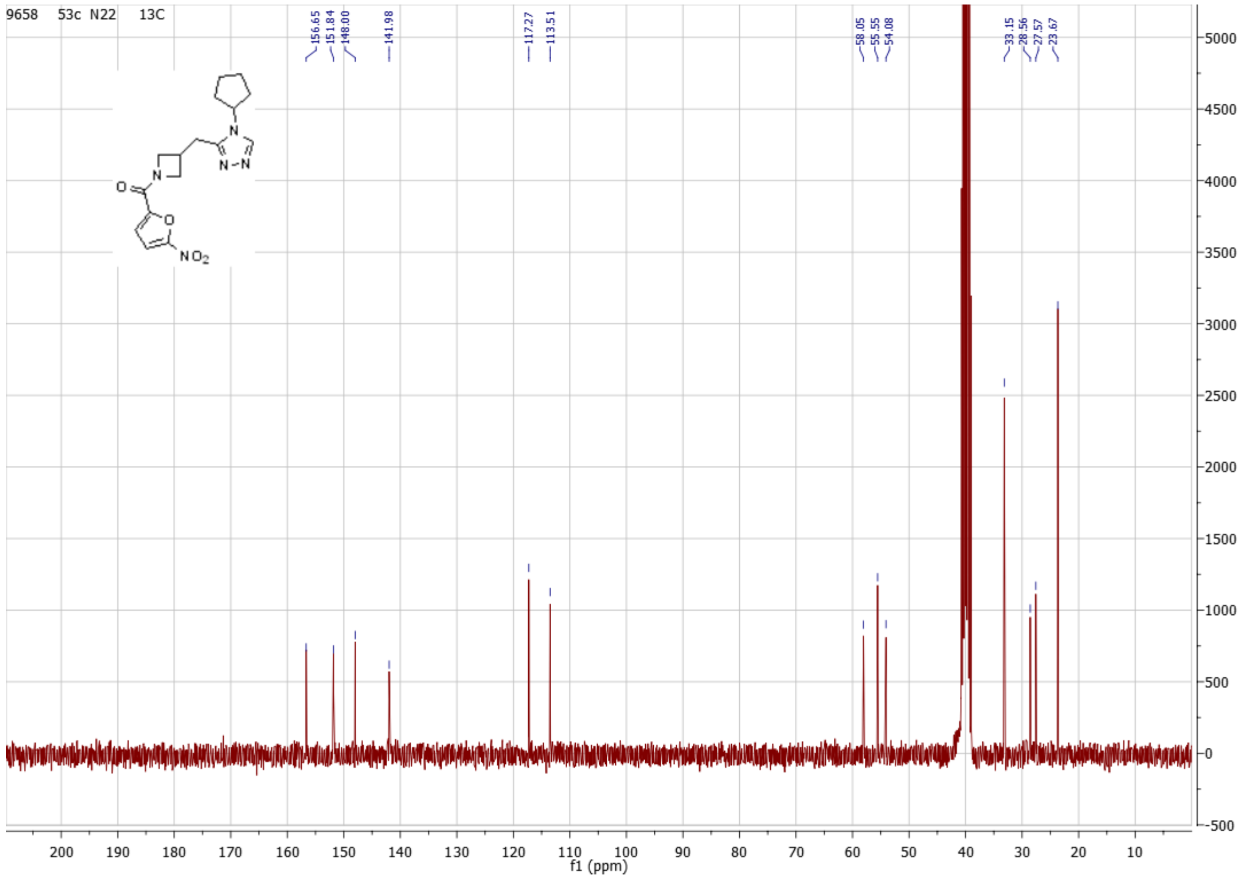

LCMS data files of target compounds

Compound	LCMS file number
1	5411
2a	5525
2b	5273
2c	5311
2d	5294
2e	5445
2f	5310
2g	5444
2h	5312
2i	5343
2j	5344
8	5685
3a	5513
3b	5705
3c	5697
3d	5706
3e	5719
4a	5192
4b	5212
4c	5208
4d	5200
5a	5483
5b	5487
5c	5484
